# Accepting your Body after Cancer (ABC), a group-based online intervention for women treated for breast cancer: study protocol for a feasibility randomised controlled trial

**DOI:** 10.1136/bmjopen-2024-097817

**Published:** 2025-01-22

**Authors:** Helena Lewis-Smith, Abigail Jones, Paul White, Sarah Byford, Patricia Fairbrother, Shelley Potter, Diana Harcourt

**Affiliations:** 1 University of the West of England, Bristol, UK; 2 King's College London, London, UK; 3 Independent Cancer Patients Voice, Bristol, UK; 4 University of Bristol, Bristol, UK

**Keywords:** Breast tumours, Psychosocial Intervention, Feasibility Studies

## Abstract

**Introduction:**

Breast cancer is the most common cancer among women worldwide. While increasing numbers of women are living beyond breast cancer, treatment-related body image concerns are common and associated with adverse consequences. Nonetheless, rigorously evaluated and effective body image interventions are lacking among this group. *Accepting your Body after Cancer (ABC*) has indicated promise in relation to inperson delivery. However, online delivery may increase accessibility and facilitate sustainability of the intervention. Therefore, we aim to establish the feasibility of conducting a fully powered randomised controlled trial to evaluate online delivery of *ABC*.

**Methods and analysis:**

120 women who have received treatment for breast cancer and are experiencing body image concerns will be randomised equally to either the *ABC* or standard care control group. All participants in both conditions will receive a body image booklet for people who have had cancer. *ABC* participants will also take part in a weekly seven-session, group-based cognitive behavioural therapy intervention, delivered online by a psychologist and cancer support specialist. Outcome measures will be completed at baseline and 9 weeks, 20 weeks and 32 weeks post baseline. Quantitative data on recruitment, retention, *ABC* attendance and questionnaire completion rates will be analysed using descriptive statistics. Qualitative data will also be collected to better understand the feasibility and acceptability of the research process and intervention, with data analysed using ‘codebook’ thematic analysis.

**Ethics and dissemination:**

The study has received ethical approval from the Newcastle North Tyneside Research Ethics Committee (ref: 24/NE/0092). The findings will be disseminated to academic and health professionals via a peer-reviewed publication and presentations at relevant conferences. Results will also be disseminated to participants, national cancer organisations and the general public via accessible reports, online presentations and different communication channels.

**Trial registration number:**

ClinicalTrials.gov NCT06412341; ISRCTN ISRCTN88199566; IRAS 327507; REC reference 24/NE/0092; funder reference NIHR205415.

**STRENGTHS AND LIMITATIONS OF THIS STUDY**

- The intervention is a scalable form of body image support for women following treatment for breast cancer, which is the point at which they often need the most psychosocial support but have the least contact with health professionals.
- Online, group-based delivery of the intervention has the potential to reduce costs, increase accessibility and facilitate sustainability of the intervention.
- Recruiting women who have finished treatment for breast cancer can be challenging, and thus the study will examine a number of different recruitment avenues, including via National Health Service hospitals, cancer support organisations, and social media.
- While not powered to assess the efficacy of the intervention, the sample size is appropriate to examine feasibility and acceptability via quantitative and qualitative data, which will inform a fully powered randomised controlled trial.

## Introduction

 Breast cancer is the most commonly diagnosed cancer among women worldwide, with approximately 2.3 million new cases recorded in 2022.^1^ However, advances in treatment have led to decreased mortality rates (12.6%), with increasing numbers of women living with and beyond breast cancer.^1^ Nonetheless, this large group of women are left with a wide range of appearance and functional changes to the body as a consequence of treatment, such as breast asymmetry, scarring, weight and skin changes, hair loss or thinning, menopause, pain and fatigue. These changes can have adverse impacts on body image,^2 3^ defined as perceptions, thoughts, feelings and behaviours relating to the body’s appearance, functions and capabilities.^4^

Systematic reviews identify body image concerns as a salient issue among women treated for breast cancer.^2 5 6^ This is supported by a recent report from ‘Breast Cancer Now’ (‘Breast Cancer Now’ is the largest breast cancer research and support charity in the UK), which found that 44% of breast cancer survivors were experiencing body image concerns.^7^ The scale of this issue is heightened in the UK, where over 1500 women who opted for a breast reconstruction were facing at least 2-year delays due to the National Health Service’s (NHS) suspension of non-urgent procedures during the COVID-19 pandemic.^7^,^9^ To add further concern, research suggests that health professionals may lack confidence in addressing body image concerns among women treated for breast cancer, with both parties often waiting for the other to initiate the discussion.^10^ Unsurprisingly, this can give the impression that health professionals fail to recognise the severity of impacts on body image and sexuality.^11 12^

Body image concerns are pervasive, with little improvement indicated 5 years following treatment.^13^ Further, the consequences of depression, anxiety, sexual and intimacy issues, poorer quality of life and shorter survival warrant attention,^14^,^17^ particularly since such psychosocial issues are more commonly reported by breast cancer survivors compared with women with no cancer history.^18^ This is costly for society as depression and anxiety among women treated for breast cancer can lead to greater healthcare use and costs, and economic losses.^19 20^ Collectively, this highlights the importance of targeting potent risk factors for adverse outcomes among breast cancer survivors, including body image concerns. However, despite the need for body image interventions for women treated for breast cancer, systematic reviews highlight the absence of rigorously evaluated interventions with lasting improvements.^21^,^23^ This informed the decision to develop *Accepting your Body after Cancer (ABC*), a body image intervention for women treated for breast cancer.

*ABC* was developed using a step-by-step approach advised by the Medical Research Council’s framework for developing and evaluating complex interventions.^24^ The first step involved conducting a systematic review of body image interventions for women in midlife, based on the premise that most women with breast cancer tend to be in midlife and beyond.^25^ This identified a rigorously evaluated and effective group-based, cognitive behavioural therapy (CBT) intervention for women in midlife, with large effects noted at 6 months follow-up.^26^ In order to explore its potential applicability to women treated for breast cancer, we explored whether the body image influences targeted in the intervention (eg, appearance comparisons, internalisation of appearance ideals) were relevant for this group, with findings providing confirmation.^27^ Based on this, we proceeded to adapt this midlife intervention for women treated for breast cancer by adding more relevant examples (eg, anxiety about wearing a wig when doing exercise) and addressing additional areas of concern (eg, intimacy). This included the input of both women treated for breast cancer and the health professionals who work with them (eg, psychologists, breast cancer nurse specialists), who provided feedback on the adapted intervention and suggested changes and new content. See Lewis-Smith for details relating to intervention adaptation.^28^

Following this, we assessed the acceptability and feasibility of inperson delivery of *ABC* to women who had been treated for breast cancer.^29^ Findings indicated low attrition, as demonstrated by 91% of the sample completing the intervention and 80% completing all assessment timepoints. Participant evaluations suggested that the intervention was acceptable, with 94.4% of the sample reporting benefits and advocating for UK-wide dissemination. Further, statistical analyses demonstrated preliminary efficacy in relation to most outcomes, including body image, self-esteem and quality of life. Once published, the study prompted global requests from health professionals for training in the delivery of the intervention. Following training from the first author in 2019, a Canadian hospital implemented the intervention. Impact data were collected from women who took part in the intervention, and this mirrored the promising findings from the acceptability study.^29^ Further, when intervention delivery was forced to shift online due to restrictions associated with the COVID-19 pandemic, impact data continued to suggest benefits, providing preliminary support for the intervention when delivered online.

While research and impact data indicate promise in relation to both inperson and online delivery of the body image intervention, the latter may help overcome barriers and increase geographical accessibility. This may also enable inclusion of women from diverse backgrounds and consequently help reduce health inequalities. Online delivery would also be less costly than inperson delivery and could facilitate sustainability of the intervention. Prior to conducting a full-scale randomised controlled trial (RCT) to establish the effectiveness of online delivery of *ABC,* a feasibility study is needed in order to inform appropriate study design parameters.

### Research objectives

We aim to assess the feasibility and acceptability of conducting an RCT to evaluate online delivery of *ABC*. Results will inform the design, management and future delivery of an evaluation to assess effectiveness and cost-effectiveness in a definitive RCT.

Specific objectives are to:

1. Establish appropriate, inclusive and acceptable methods of participant recruitment, retention and management procedures.
2. Establish the feasibility and acceptability of quantitative data collection, including determining appropriate primary and secondary outcome measures.
3. Adapt and test a measure of health and social care service use, to inform a future economic evaluation.
4. Establish intervention adherence and acceptability (of the online setting) among participants and facilitators.

## Methods and analysis

### Design

**Figure 1:**
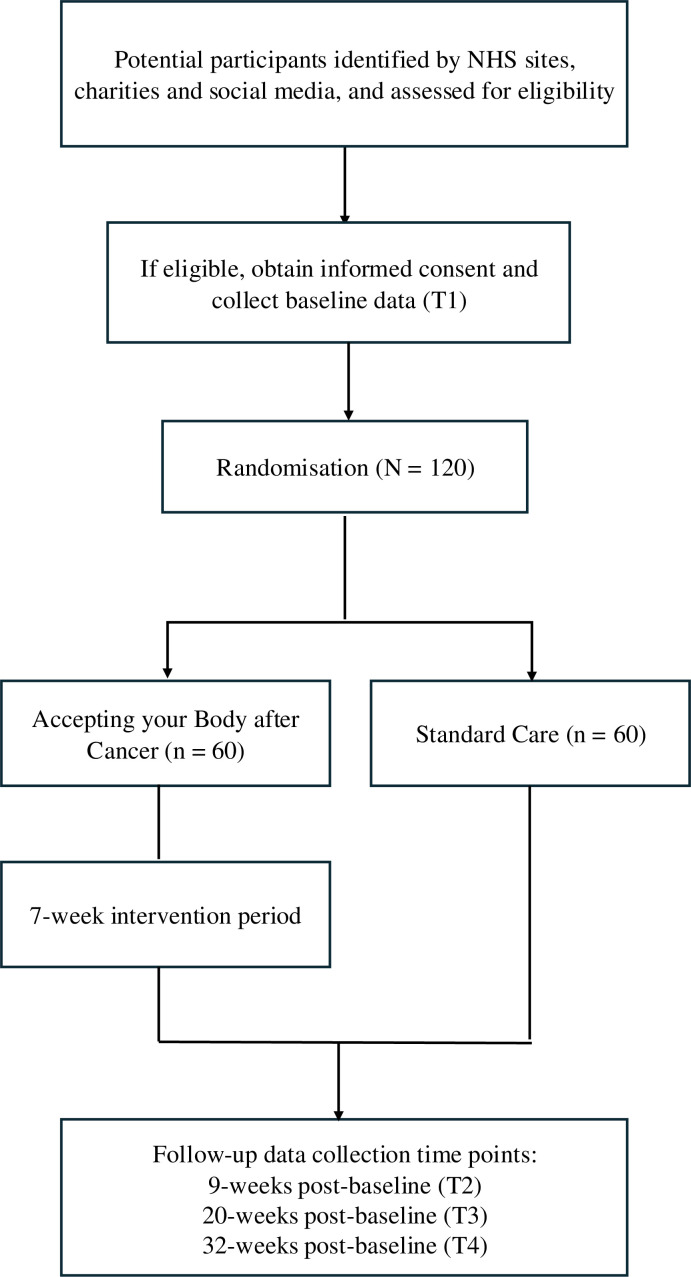
Study flow of participation. NHS, National Health Service.

This is a feasibility study to assess the feasibility and acceptability of the research process and of the *ABC* programme (online supplemental file 1). To do this, we will use a parallel, two-arm, RCT in which we will monitor participant recruitment and retention, and use qualitative interviews to provide qualitative feedback. Please see figure 1 for the participant pathway through the study.

### Eligibility criteria

To be eligible to participate in the study, individuals must fulfil the following inclusion criteria:

- Identify as a woman.
- 18+ years old.
- Finished active treatment for breast cancer (including chemotherapy, radiotherapy, targeted and immunotherapy) for breast cancer. There is no time limit on when they finished this treatment. Women on endocrine therapy are eligible to take part. Women with metastatic disease are eligible to take part if on endocrine therapy only.
- Completed primary oncological breast cancer surgery with breast-conserving surgery or mastectomy with or without immediate definitive breast reconstruction. Women awaiting delayed breast reconstruction, revision or contralateral symmetrisation surgery are eligible to take part, provided this surgery is not planned within the duration of the study.
- Recognises that they are experiencing body image distress as a result of treatment (regarding how the body looks and/or feels).
- Has the capacity to provide informed consent or supported informed consent (eg, with a family member/friend).
- Has sufficient understanding of English (as the intervention content and measures are currently only available in English).

Participants will be excluded from participation if they meet any of the following exclusion criteria:

- Still undergoing active treatment for breast cancer (eg, oncological breast surgery including those awaiting the second stage of planned expander/implant reconstruction, chemotherapy, targeted therapies, radiotherapy).
- Undergoing exploration for cancer recurrence.
- Has not received a diagnosis of breast cancer, for example, has had prophylactic treatment for a gene mutation (such as risk-reducing mastectomy).
- Has an eating disorder.
- Unable to provide informed consent.

### Proposed sample size

We will recruit n=120 (60 per arm), with participants individually randomised to either the intervention arm (*ABC* programme plus Macmillan body image booklet) or control arm (Macmillan body image booklet). The objective of this feasibility study is to provide estimates of parameters to inform a subsequent RCT to evaluate intervention effectiveness. Therefore, the study does not need to achieve enough power to detect significant differences, and a formal a priori power calculation is not needed. Rather, the sample size is based on the aim of having a fully powered follow-on substantive or definitive trial, and to help quantify the degree of missing data. Data will be used to estimate the upper one-sided 80% CI for the pooled variance of postintervention self-report outcome measures. For an 80% chance of not being underpowered at any level of power, and for any minimum clinically important difference, a sample of 60 per condition would ensure that the percentage error in estimated sample size for a definitive trial would be no more than 11% (ie, potentially overpowered, but degree of excess restricted to 11%). The estimated sample size for the proposed definitive study can be refined further by estimating the strength of the pre- post-correlation and using these estimates in estimating sample size for repeated measures Analysis of Covariance (ANCOVA)-styled analyses (controlling for commensurate baseline methods), thus resulting in a cost-effective substantive RCT without compromising the reliability of conclusions.

### Participant recruitment

We aim to recruit a diverse group of participants, so we will recruit participants through NHS Participant Identification Centres (PICs), a range of non-NHS organisations (eg, Breast Cancer Now, Maggie’s, Keeping Abreast, Flat Friends, OUTpatients and Black Women Rising) and social media. The PICs will provide study information packs to interested women who have expressed dissatisfaction with their body. The study information pack includes the information sheet and expression of interest form. PICs will also display study posters, which will be used to promote the study through non-NHS organisations and social media. The study posters will include contact details for the study team and signposts to the online information sheet and expression of interest form. Potential participants can contact the study team directly or by completing the online or paper expression of interest form. The study team will then discuss the study with participants over the telephone, ensuring that they understand what is involved in participation and confirming eligibility. Following successful screening, the participant will be sent the online consent form and baseline measures.

### Study setting

Participants recruited through NHS sites will either be approached in person at their standard nurse appointment or via email or post. All other study procedures, including recruitment through non-NHS organisations and social media, screening, consent, data collection and intervention delivery, will be conducted remotely. This remote approach offers a convenient, effective and flexible approach.

### Randomisation

Once participants have provided informed consent (online supplemental appendix 1) and completed baseline data collection (T1), they will be randomised to either the intervention or control arm using Sealed Envelope, a web-based randomisation system. Randomisation (at the individual level) will be independent and concealed using permuted block randomisation. Participants will be informed of the result of randomisation via email, along with the Macmillan body image booklet. Participants randomised to take part in the *ABC* programme will then be booked on to the next suitable group.

### The intervention: *ABC*

The *ABC* programme comprises seven 2-hour group sessions (with approximately 10 women per group) delivered online weekly via Microsoft Teams. Each *ABC* group will be delivered jointly by a cancer support specialist (eg, specialist nurse) and psychologist from a Maggie’s centre (Maggie’s is a charity that provides free expert care and support in centres across the UK and online). However, there will be a different pair of facilitators across each group, with some running more than one group (depending on their capacity).

The intervention aims to improve body image among women treated for breast cancer. Rooted in CBT, the *ABC* programme uses strategies to alter unhelpful thoughts, reduce anxiety and promote non-avoidant behaviours. The programme also explores sociocultural pressures for women, intimacy, physical activity, self-care, mindfulness and relaxation. The sessions are guided using PowerPoint slides and will include individual and group-based activities. Participants are asked to complete readings to prepare for each session and activities to help them to apply the techniques to their day-to-day lives. Table 1 provides an overview of *ABC* and its content.

**Table 1:** Accepting your Body after Cancer content overview

Session	Content
1	Introduction to body image Personal reflection on the impact of body image concerns Exploration of personal goals
2	Introduction to the cognitive behavioural therapy approach Physiological symptoms of anxiety Exploration of body image and self-esteem Relaxation training
3	Stopping negative body-related self-talk Developing alternative, balanced thoughts Planning a self-care activity schedule Relationship between body function and movement
4	Sociocultural pressures for women in midlife Internalisation of the youthful-thin ideal Body comparisons—experimental activity Body nurture with accepting self-talk
5	Exploration of relationships and intimacy Managing people’s reactions Cognitive restructuring process Physical activity and movement
6	Identifying core beliefs Modifying mistaken beliefs Engaging the senses—mindful eating Relaxation exercise
7	Positive body affirmations Reducing the chances of a setback Dealing with a setback Future plans

Prior to the start of the group, participants will be sent a guide concerning session expectations (eg, format, activities), clear instructions on how to use Microsoft Teams and all intervention materials. Any participants who do not have access to the internet (or have limited data use) will be provided with a data internet card.

Prior to the first *ABC* session, participants will be asked to read the pre-session intervention materials (ie, Session 1: Part 1) to help them start reflecting on their goals. In the session, there will be introductions and discussion of ground rules before the structured session content is delivered. All participants will be asked to have the second part of the intervention materials for the session (ie, Session 1: Part 2) in front of them to facilitate individual and group exercises. At the end, they will also be asked to complete two between-session activities at home (eg, mirror exposure exercise) before the next session. They will also be advised to read the pre-session intervention materials (ie, Session 2: Part 1) for the following week’s session. The same format then continues for all sessions.

### Training of facilitators

All facilitators will have experience of running support groups for those who have had cancer and experience of working specifically with women who have had breast cancer. Facilitators will receive 1 day of online training from the first author and will be provided with all the relevant materials. Facilitators will receive ongoing supervision from the first author and will provide feedback on the programme.

### Standard care

There is no current ‘treatment-as-usual’ in relation to body image support for women treated for breast cancer; however, Macmillan have produced a body image booklet for people who have had cancer which is currently freely available. This booklet provides a substantial amount of support and guidance relating to managing body image concerns. It explains the effects of cancer on body image and provides practical guidance (eg, make-up) and psychoeducational guidance (eg, managing others’ reactions), in addition to some CBT strategies. The Macmillan booklet is therefore a suitable source of information and support for participants in the control arm, who will be sent the booklet and encouraged to work through it gradually when informed of their allocation to the control arm.

### Intervention fidelity

Intervention fidelity will be assessed across different *ABC* groups and sessions. All *ABC* sessions will be recorded, with 50% then assessed by the research team for the perceived competency (percentage reflecting how well facilitators delivered the sessions) and intervention adherence (percentage reflecting the extent to which facilitators completed each section of the sessions).

### Data management

Qualtrics will be used to manage consent and data collection throughout the study. Data will be protected via a password and backed up automatically. Data will be exported to SPSS for data cleaning and analysis. We will regularly check data to ensure high data quality.

### Outcomes

Data will be collected at four timepoints: baseline (T1), 9 weeks post baseline (T2; immediate post intervention) 20 weeks post baseline (T3) and 32 weeks post baseline (T4). See table 2 for a detailed breakdown of the schedule of assessments.

**Table 2:** Schedule of assessments

	Baseline (T1)	9 weeks post baseline (T2)	20 weeks post baseline (T3)	32 weeks post baseline (T4)
Consent and general information				
Consent	X			
Demographics	X			
Cancer-related information	X			
Self-reported primary validated outcome measures				
Kessler Psychological Distress Scale K10	X	X	X	X
Body Appreciation Scale-2	X	X	X	X
Functional Assessment of Cancer Therapy—Breast Version 4: Breast Cancer Subscale	X	X	X	X
Self-reported secondary validated outcome measures				
Hopwood Body Image Scale	X	X	X	X
BREAST-Q: Sexual Well-Being Scale	X	X	X	X
Health economic measures				
Modified version of the Adult Service Use Schedule	X		X	X
EQ-5D-5L	X		X	X
Recovering Quality of Life-Utility Index-10	X		X	X
Work and Social Adjustment Scale	X		X	X
Acceptability assessments				
*ABC* Acceptability Rating Scales		X		
*ABC* Acceptability Open-Ended Questions		X		
Research Process Acceptability Ratings Scales				X
Research Process Acceptability Open-Ended Questions				X
Interviews	As appropriate			

ABCAccepting your Body after Cancer

#### Quantitative outcomes

We will collect the following quantitative data to assess the feasibility and acceptability of the research design and *ABC*:

- Rates of recruitment (and associated response rates), with attention to the method by which women were recruited and their demographic diversity.
- Proportion completing *ABC* (number of sessions attended and between-session activities completed).
- Proportion of control arm reading the Macmillan body image booklet (plus percentage of booklet read).
- Proportion completing outcome measures at each assessment (plus data completeness rates).
- Rating scales to explore the acceptability of *ABC* (eg, the group format, online nature, between-session activities)—to be completed by intervention participants at T2
- Rating scales to explore the acceptability of the research process generally (eg, information provided about the study, randomisation process, questionnaires)—to be completed by all participants at T4.

#### Qualitative outcomes

In addition to quantitative data, we will collect the following qualitative data to assess the feasibility and acceptability of the research design and *ABC*:

- Open-ended questions to explore the acceptability of *ABC* (eg, the group format, online nature, between-session activities)—to be completed by *ABC* participants at T2
- Open-ended questions to explore the acceptability of the research process generally (eg, information provided about the study, randomisation process, questionnaires)—to be completed by all participants at T4
- Interviews with a subset of participants (including those who withdraw) to explore their experiences in relation to recruitment, randomisation, the Macmillan body image booklet, communication from the research team throughout the study and completion of outcome measures. Participants from the intervention arm will also be asked about the acceptability of the *ABC* intervention.

#### Suitability of outcome measures

Data relating to self-report validated outcome measures will be collected to determine the suitability of these measures for use in a subsequent definitive RCT, rather than to draw conclusions about the relative efficacy of *ABC*.

Demographics (eg, age, ethnicity, relationship status) and details of cancer diagnosis and treatment (eg, stage of cancer, time since finishing active treatment, modes of treatment received) will be collected from participants at T1.

The following proposed primary outcome measures will be assessed at all four timepoints:

- Kessler Psychological Distress Scale (K10):^30^ This is a widely used 10-item global measure of psychological distress based on questions relating to anxiety and depressive symptoms that have been experienced in the previous 30 days. Responses are based on a 5-point Likert-type scale and are summed to generate a total score, with higher scores indicating greater psychological distress.
- Body Appreciation Scale-2 (BAS-2):^31^ This is a commonly used 10-item measure of body appreciation, which is a core facet of positive body image and refers to acceptance, favourable opinions and respect towards one’s body. Responses are based on a 5-point Likert-type scale and are averaged to generate an overall score, with higher scores indicating greater body appreciation.
- Functional Assessment of Cancer Therapy—Breast (FACT-B Version 4): Breast Cancer Subscale:^32^ The FACT-B is a frequently used measure of health-related quality of life for patients with breast cancer. The 10-item Breast Cancer Subscale will be used, whereby statements are considered in relation to the previous 7 days. Responses are based on a 5-point Likert-type scale and are averaged to generate an overall score, with higher scores indicating better quality of life.

The following proposed secondary outcome measures will be assessed at all four timepoints:

- Hopwood Body Image Scale:^33^ This is a widely used 10-item measure of body image in patients with cancer and measures affective, behavioural and cognitive elements of body image. Responses are based on a 4-point Likert-type scale and are summed to generate a total score, with higher scores indicating poorer body image.
- BREAST-Q: Sexual Well-Being Scale:^34^ The BREAST-Q is a frequently used outcome measure for use in cosmetic and reconstructive breast surgery and clinical practice. The six-item Sexual Well-Being Subscale will be used, whereby feelings in relation to sexual confidence and comfort during sex will be considered using a 5-point Likert-type scale. Responses are summed to generate a total score, with higher scores indicating greater sexual well-being.

The following proposed measures for economic evaluation will be assessed at T1, T3 and T4:

- Modified version of the Adult Service Use Schedule (AD-SUS):^35^ This measure takes an NHS and social services perspective and has been adapted to ensure coverage of services relevant to women treated for breast cancer. The measure, completed at T1, T3 and T4, asks participants about their use of different health and social services over the previous 3 months (at T1) or since last data completion point (at the T3 and T4 follow-ups).
- EQ-5D-5L:^36^ This is a widely used standardised measure of health status that assesses health-related quality of life across five dimensions, including mobility, self-care, usual activities, pain/discomfort and anxiety/depression. It also includes a visual analogue scale to assess the respondent’s overall current health.
- Recovering Quality of Life-Utility Index (ReQoL-10):^37 38^ While the use of the EQ-5D-5L is recommended by the National Institute for Health and Care Excellence, patient and public involvement and engagement (PPIE) advisors raised concerns that this measure may not adequately capture psychological well-being. Therefore, the 10-item ReQoL-10 will also be used due to its broader focus on psychological well-being, and the ReQoL Utility Index will be generated.
- Work and Social Adjustment Scale (WSAS):^39^ This five-item measure assesses the impact of one’s psychological well-being on their ability to function in terms of work, home management, social leisure, private leisure and personal or family relationships. PPIE advisors felt that this measure captured impacts on different domains of their life. The items are summed to produce a score indicating low, moderate or high work and social impairment.

### Data analysis

The feasibility and acceptability of the *ABC* programme and the research process will be quantitatively assessed through descriptive statistical analyses of monitoring data and self-report rating scales. We will report data in line with the Consolidated Standards of Reporting Trials 2010 Statement showing attrition rates and loss to follow-up. In addition, feasibility and acceptability will be qualitatively assessed using ‘codebook’ thematic analysis of open-ended questions and interviews, whereby text will be coded using a priori codes and themes relating to feasibility and acceptability.^40^

The self-report outcome measures (K10, BAS-2, FACT-B: Breast Cancer Subscale, Hopwood Body Image Scale and BREAST-Q: Sexual Well-Being Scale) are each well-established validated measures which will be scored according to the most appropriate guidelines. The design (pre- post- two-arm parallel RCT) and the selected measures are amenable to analysis using long-established statistical techniques. The analysis will proceed using the intention-to-treat analysis set with the statistician blinded to allocation. These analyses include

- Descriptive statistics (including mean and SD) by randomised arm at each timepoint (T1, T2, T3 and T4).
- Unadjusted statistical comparison between arms at each timepoint (T1, T2, T3 and T4) using the independent-samples *t*-test with 95% and 80% CIs and quantification of effect size using Cohen’s *d*. If statistical assumptions do not seem tenable, robust non-parametric bootstrap equivalent tests will be used.
- Adjusted statistical comparison between arms at each timepoint (T2, T3, T4) will be conducted controlling for commensurate baseline measures using ANCOVA, along with 95% and 80% covariate-adjusted CIs and quantification of effect size using partial η^2^. If statistical assumptions do not seem tenable, robust remedies will be considered, including a baseline by randomised arm interaction effect, or homoscedasticity-corrected robust standard errors (HC3) or the robust non-parametric bootstrap equivalent ANCOVA.

The draft *ABC* AD-SUS will undergo testing for the following:

- Acceptability: this will be assessed via the proportion of participants completing the measure, percentage of missing items and consideration of the clarity of any questions associated with missing data.
- Comprehensiveness: this will be assessed via open-ended questions included in the AD-SUS asking participants to report any ‘other’ services not reported elsewhere in the measure (missing items).
- Redundancy: this will be assessed through consideration of any service items included in the AD-SUS but not reported as being used by any participant (redundant items).

These results, plus feedback from participants, will inform a final version of the AD-SUS for a future definitive trial.

Acceptability of the EQ-5D-5L, ReQoL-10 and WSAS will be assessed using completion rates (proportion of participants completing each of the measures and percentage of missing items within each of the measures), plus feedback from participants. Service use, health-related quality of life and the WSAS will be summarised and reported descriptively.

### Patient and public involvement and engagement

The study has benefited from substantial PPIE. This has included a PPIE lead and coapplicant who had extensive involvement in the design of the study. In addition to the PPIE lead, there has been PPIE involvement throughout every stage of the development of *ABC* and its preliminary evaluation. Women treated for breast cancer, associated health professionals (eg, cancer nurse specialists, psychologists) and cancer support organisations (eg, Breast Cancer Now) contributed to the adaptation of the original intervention and its acceptability testing. A study public advisory group comprising a diverse group of women who had been treated for breast cancer have informed several aspects of the study including the research protocol, participant-facing research materials and the *ABC* programme materials. Along with the PPIE lead, this public advisory group will continue to advise the study team through the analysis and dissemination stages.

## Ethics and dissemination

The study received ethical approval by an NHS Research Ethics Committee (ref: 24/NE/0092), full Health Research Authority approval (IRAS: 327507) and University ethical approval (ref: CHSS.24.06.222) prior to the commencement of research activities. Prior to participation, participants will be provided with a participant information sheet which has been coproduced with the PPIE lead and public advisory group. Following receipt of the information sheet, participants will discuss the study over the phone or email with the study team. This discussion will answer any questions that the participant may have and confirm their eligibility for the study. The information sheet and these discussions will ensure that the participants are fully informed about the study and what participation involves. Once participants have made a decision to take part, they will provide informed consent prior to data collection and will have a unique participant ID to facilitate participant confidentiality and allow participants to withdraw their data if they so wish. Participants will be informed of their right to withdraw from the study through the information sheet and in discussions with the research team.

Results of this study will be disseminated to academic and medical audiences through a peer-reviewed publication, presentations at relevant conferences such as the British Psycho-Oncology Society Conference in 2025, and Appearance Matters 11 in 2026.

Results will also be disseminated to participants and national cancer organisations (eg, Breast Cancer Now, Macmillan, Maggie’s, Flat Friends, Keeping Abreast, Black Women Rising, OUTPatients) through a report and online presentations. The report will be designed to be engaging (eg, infographics, not text-heavy) and will be developed in collaboration with the PPIE lead and the public advisory group. There will be an accessible online presentation of the findings for participants who wish to attend, with a similar presentation scheduled for the national cancer organisations. Finally, the findings will be shared via the communication channels (eg, press release, social media, podcasts) of the national cancer organisation and the University of the West of England.

### Trial steering committee

Independent oversight and supervision of the study will be provided by the trial steering committee. The steering committee will meet twice a year to monitor the progress and conduct of the study, and where relevant provide clinical and professional advice relating to the study. The steering committee will comprise a professor in long-term conditions, lived experience representatives, a professor of psychosocial oncology, a clinical psychologist and service lead for a cancer support centre, an associate director of services for a national cancer organisation and a chief nursing officer for a national cancer organisation.

## supplementary material

10.1136/bmjopen-2024-097817 online supplemental file 1

10.1136/bmjopen-2024-097817 online supplemental file 2

## References

[1] Bray F, Laversanne M, Sung H (2024). Global cancer statistics 2022: GLOBOCAN estimates of incidence and mortality worldwide for 36 cancers in 185 countries. CA Cancer J Clin.

[2] Thakur M, Sharma R, Mishra AK (2022). Body image disturbances among breast cancer survivors: A narrative review of prevalence and correlates. Cancer Res Stat Treat.

[3] Rodrigues ECG, Neris RR, Nascimento LC (2023). Body image experience of women with breast cancer: A meta-synthesis. Scand J Caring Sci.

[4] Whitbourne SK, Skultety K, Cash TF, Pruzinsky T (2002). Body image: A handbook of theory, research, and clinical practice.

[5] Davis C, Tami P, Ramsay D (2020). Body image in older breast cancer survivors: A systematic review. Psychooncology.

[6] Paterson CL, Lengacher CA, Donovan KA (2016). Body Image in Younger Breast Cancer Survivors: A Systematic Review. Cancer Nurs.

[7] Breast Cancer Now (2022). 44% of women with breast cancer say it negatively impacted their body image.

[8] British Association of Aesthetic Plastic Surgeons (2020). Re-establishing breast reconstruction services.

[9] Association of Breast Surgery (2020). Statement for the association of breast surgery, 15th March 2020.

[10] Cohen M, Anderson RC, Jensik K (2012). Communication between breast cancer patients and their physicians about breast-related body image issues. Plast Surg Nurs.

[11] McWilliam CL, Brown JB, Stewart M (2000). Breast cancer patients’ experiences of patient-doctor communication: a working relationship. Patient Educ Couns.

[12] Rosman S (2004). Cancer and stigma: experience of patients with chemotherapy-induced alopecia. Patient Educ Couns.

[13] Falk Dahl CA, Reinertsen KV, Nesvold I-L (2010). A study of body image in long-term breast cancer survivors. *Cancer*.

[14] Cousson-Gélie F, Bruchon-Schweitzer M, Dilhuydy JM (2007). Do anxiety, body image, social support and coping strategies predict survival in breast cancer? A ten-year follow-up study. Psychosomatics.

[15] Begovic-Juhant A, Chmielewski A, Iwuagwu S (2012). Impact of body image on depression and quality of life among women with breast cancer. J Psychosoc Oncol.

[16] Moreira H, Canavarro MC (2010). A longitudinal study about the body image and psychosocial adjustment of breast cancer patients during the course of the disease. Eur J Oncol Nurs.

[17] Lam WWT, Li WWY, Bonanno GA (2012). Trajectories of body image and sexuality during the first year following diagnosis of breast cancer and their relationship to 6 years psychosocial outcomes. Breast Cancer Res Treat.

[18] Forbes H, Carreira H, Funston G (2024). Early, medium and long-term mental health in cancer survivors compared with cancer-free comparators: matched cohort study using linked UK electronic health records. *eClinicalMedicine*.

[19] Van Beek FE, Wijnhoven LMA, Holtmaat K (2021). Psychological problems among cancer patients in relation to healthcare and societal costs: A systematic review. Psychooncology.

[20] Mausbach BT, Decastro G, Schwab RB (2020). Healthcare use and costs in adult cancer patients with anxiety and depression. Depress Anxiety.

[21] Sebri V, Durosini I, Triberti S (2021). The Efficacy of Psychological Intervention on Body Image in Breast Cancer Patients and Survivors: A Systematic-Review and Meta-Analysis. Front Psychol.

[22] Morales-Sánchez L, Luque-Ribelles V, Gil-Olarte P (2021). Enhancing Self-Esteem and Body Image of Breast Cancer Women through Interventions: A Systematic Review. Int J Environ Res Public Health.

[23] Lewis-Smith H, Diedrichs PC, Rumsey N (2018). Efficacy of psychosocial and physical activity-based interventions to improve body image among women treated for breast cancer: A systematic review. Psychooncology.

[24] Skivington K, Matthews L, Simpson SA (2021). A new framework for developing and evaluating complex interventions: update of Medical Research Council guidance. BMJ.

[25] Lewis-Smith H, Diedrichs PC, Rumsey N (2016). A systematic review of interventions on body image and disordered eating outcomes among women in midlife. Int J Eat Disord.

[26] McLean SA, Paxton SJ, Wertheim EH (2011). A body image and disordered eating intervention for women in midlife: a randomized controlled trial. J Consult Clin Psychol.

[27] Lewis-Smith H, Diedrichs PC, Bond R (2020). Psychological and sociocultural influences on body image among midlife women with and without a history of breast cancer: Testing the Tripartite Influence Model of Body Image. Body Image.

[28] Lewis-Smith H (2017). Body Image in Midlife: Developing a Psychosocial Intervention for Women Who Have Received Treatment for Breast Cancer.

[29] Lewis-Smith H, Diedrichs PC, Harcourt D (2018). A pilot study of a body image intervention for breast cancer survivors. Body Image.

[30] Kessler RC, Andrews G, Colpe LJ (2002). Short screening scales to monitor population prevalences and trends in non-specific psychological distress. Psychol Med.

[31] Tylka TL, Wood-Barcalow NL (2015). The Body Appreciation Scale-2: item refinement and psychometric evaluation. Body Image.

[32] Brady MJ, Cella DF, Mo F (1997). Reliability and validity of the Functional Assessment of Cancer Therapy-Breast quality-of-life instrument. J Clin Oncol.

[33] Hopwood P, Fletcher I, Lee A (2001). A body image scale for use with cancer patients. Eur J Cancer.

[34] Pusic AL, Klassen AF, Scott AM (2009). Development of a New Patient-Reported Outcome Measure for Breast Surgery: The BREAST-Q. Plast Reconstr Surg (1946).

[35] Strauss C, Arbon A, Barkham M (2020). Low-Intensity Guided Help Through Mindfulness (LIGHTMIND): study protocol for a randomised controlled trial comparing supported mindfulness-based cognitive therapy self-help to supported cognitive behavioural therapy self-help for adults experiencing depression. Trials.

[36] Herdman M, Gudex C, Lloyd A (2011). Development and preliminary testing of the new five-level version of EQ-5D (EQ-5D-5L). Qual Life Res.

[37] Keetharuth AD, Brazier J, Connell J (2018). Recovering Quality of Life (ReQoL): a new generic self-reported outcome measure for use with people experiencing mental health difficulties. Br J Psychiatry.

[38] Keetharuth AD, Rowen D, Bjorner JB (2021). Estimating a Preference-Based Index for Mental Health From the Recovering Quality of Life Measure: Valuation of Recovering Quality of Life Utility Index. Value Health.

[39] Mundt JC, Marks IM, Shear MK (2002). The Work and Social Adjustment Scale: a simple measure of impairment in functioning. Br J Psychiatry.

[40] O’Cathain A, Hoddinott P, Lewin S (2015). Maximising the impact of qualitative research in feasibility studies for randomised controlled trials: guidance for researchers. Pilot Feasibility Stud.

