# Supplementary material for: Accepting your Body after Cancer (ABC), a group-based online intervention for women treated for breast cancer: study protocol for a feasibility randomised controlled trial
Source: BMJ Open. 2025 Jan 22;15(1):e097817. doi: 10.1136/bmjopen-2024-097817 (PMC11784239; doi:10.1136/bmjopen-2024-097817)
Supplement: online supplemental file 1 [file bmjopen-15-1-s001.docx]

**Full Study Title:**  **A feasibility study to inform a Randomised Controlled Trial to evaluate ‘Accepting your Body after Cancer’ (ABC), an online-delivered group-based cognitive behavioural therapy (CBT) body image intervention, for women who have received treatment for breast cancer**

**Short Study Title/Acronym:** ABC Feasibility Study

**Sponsor:** University of the West of England

**Protocol Version Number:** 3.0

**Protocol Date:** 24/10/2024

**RESEARCH REFERENCE NUMBERS**

| **IRAS ID:** | **327507** |
| --- | --- |
| **REC Reference Number:** | **24/NE/0092** |
| **ClinicalTrials.gov Number:** | **10473704** |
| **Funder reference:** | **NIHR205415** |

**Keywords: *Body image; Breast cancer; Feasibility; RCT***

# SIGNATURE PAGE

The undersigned confirm that the following protocol has been agreed and accepted and that the Chief Investigator agrees to conduct the trial in compliance with the approved protocol and will adhere to the principles outlined in the Medicines for Human Use (Clinical Trials) Regulations 2004 (SI 2004/1031), amended regulations (SI 2006/1928) and any subsequent amendments of the clinical trial regulations, GCP guidelines, the Sponsor’s (and any other relevant) SOPs, and other regulatory requirements as amended.

I agree to ensure that the confidential information contained in this document will not be used for any other purpose other than the evaluation or conduct of the clinical investigation without the prior written consent of the Sponsor

I also confirm that I will make the findings of the trial publicly available through publication or other dissemination tools without any unnecessary delay and that an honest accurate and transparent account of the trial will be given; and that any discrepancies and serious breaches of GCP from the trial as planned in this protocol will be explained.

| **For and on behalf of the Trial Sponsor:** | | |
| --- | --- | --- |
| 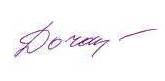Signature: |  | Date: 26/04/2024 |
| Print Name: Professor Olena Doran |  |  |
| Position: Dean Of Research and Enterprise, College of Health, Science and Society, University of the West of England | | |
| **Chief Investigator:** | | |
| Signature: 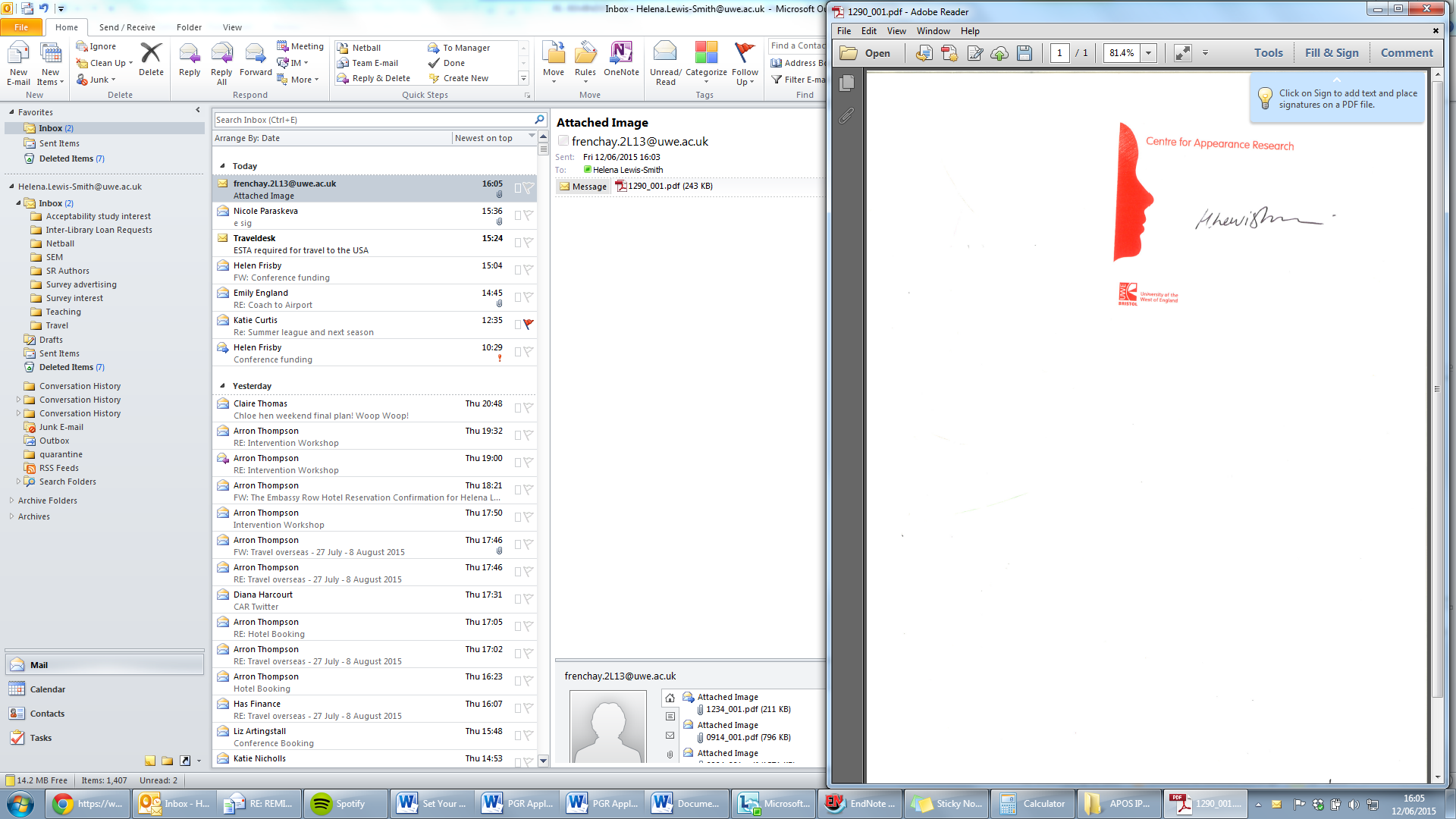 |  | Date:  26/04/2024 |
| Print Name: Helena Lewis-Smith |  |  |

# CONTACT LIST

| Chief Investigator | Dr Helena Lewis-Smith  Associate Professor of Psychology  University of the West of England, Coldharbour Lane, Bristol, BS16 1QY  Email: helena.lewis-smith@uwe.ac.uk  Tel: +4411732 81895 |
| --- | --- |
| Study Manager | Dr Abigail Jones  Research Fellow  University of the West of England, Coldharbour Lane, Bristol, BS16 1QY  Email: abbie4.jones@uwe.ac.uk |
| Sponsor | University of the West of England  Contact: Leigh Taylor  University of the West of England, Coldharbour Lane, Bristol, BS16 1QY  Email: leigh.taylor@uwe.ac.uk  Tel: 01173281170 |
| Funder(s) | National Institute for Health Research - Research for Patient Benefit (RfPB)  Grange House, 15 Church Street, Twickenham, TW1 3NL  Email: rfpb@nihr.ac.uk  Tel: 02088438057 |
| Statistician | Professor Paul White  University of the West of England, Coldharbour Lane, Bristol, BS16 1QY  Email: paul.white@uwe.ac.uk |
| Lead NHS R&D | North Bristol NHS Trust  Contact: Agnieszka Long-Batko  North Bristol NHS Trust, Research & Development, North Bristol NHS Trust, Southmead Hospital, Bristol, BS10 5NB  Email: agnieszka.long-batko@nbt.nhs.uk  Tel: 01174149930 |
| Protocol Development Group | Dr Helena Lewis-Smith  Associate Professor of Psychology  University of the West of England, Coldharbour Lane, Bristol, BS16 1QY  Email: helena.lewis-smith@uwe.ac.uk  Tel: +4411732 81895  Dr Abigail Jones  Research Fellow  University of the West of England, Coldharbour Lane, Bristol, BS16 1QY  Email: abbie4.jones@uwe.ac.uk  Professor Diana Harcourt  Professor of Appearance and Health Psychology, University of the West of England  Email: diana2.harcourt@uwe.ac.uk  Professor Paul White  Professor Applied Statistics, University of the West of England, Coldharbour Lane, Bristol, BS16 1QY  Email: paul.white@uwe.ac.uk  Professor Sarah Byford  Professor of Health Economics, Kings College London  Email: sarah.byford@kcl.ac.uk  Professor Shelley Potter  Professor of Surgical Oncology, University of Bristol  Email: shelley.potter@bristol.ac.uk  Patricia Fairbrother  PPI Lead, Independent Cancer Patients Voice  Email: [patriciafairbrother@gmail.com](mailto:patriciafairbrother@gmail.com) |

# i: LIST OF CONTENTS

| **INFORMATION** | **Page No.** |
| --- | --- |
| TITLE PAGE | 1 |
| RESEARCH REFERENCE NUMBERS | 2 |
| SIGNATURE PAGE | 3 |
| KEY TRIAL CONTACTS | 4 |
| i. LIST of CONTENTS | 6 |
| ii. LIST OF ABBREVIATIONS | 9 |
| iii. TRIAL SUMMARY | 11 |
| **SECTION** | |
| 1. BACKGROUND    1. The issue: Body image distress among breast cancer survivors    2. The intervention: Accepting your Body After Cancer    3. Rationale for the study | 13  13  13  14 |
| 1. AIMS AND OBJECTIVES | 14 |
| 1. STUDY DESIGN | 15 |
| 1. PARTICIPANT ELIGIBILITY CRITERIA    1. Inclusion criteria    2. Exclusion criteria | 16  16  16 |
| STUDY PROCEDURES AND MEASUREMENTS  - 1. Identification and recruitment of participants   2. Screening   3. Informed consent   4. Randomisation   5. Blinding   6. Data collection      1. Quantitative measures of feasibility and acceptability         1. Monitoring data         2. Self-report rating scales included in outcome assessments (see 5.6.3)      2. Qualitative measures of feasibility and acceptability         1. Open-ended questions included in outcome assessments (see 5.6.3)         2. Interviews      3. Self-report outcome measures         1. Demographics and cancer-related information         2. Primary outcome measures         3. Secondary outcome measures      4. Health economic measures   7. Gift Voucher | 16  16  18  18  18  19  19  19  19  19  20  20  23  23  24  24  24  25  26 |
| 1. INTERVENTION AND CONTROL    1. ABC Intervention    2. Intervention facilitators and fidelity    3. Control arm | 26  26  27  28 |
| 1. DATA ANALYSIS    1. Primary aims       1. Analysis of quantitative data          1. Monitoring data          2. Self-report rating scales       2. Analysis of qualitative data          1. Open-ended questions          2. Interviews    2. Analysis of self-report outcome measures    3. Heath economic evaluation | 28  28  28  28  29  29  29  29  29  30 |
| 1. DATA MANAGEMENT    1. Data collection    2. Access to data    3. Archiving | 30  30  31  31 |
| 1. ETHICAL AND REGULATORY CONSIDERATIONS    1. HRA approval    2. University REC approval    3. Amendments       1. Substantial amendments       2. Minor amendments    4. Good clinical practice    5. Protocol Violations and Serious Breaches    6. Financial and other competing interests    7. Indemnity    8. Care during and following the study    9. End of Study | 32  32  32  32  32  32  32  32  33  33  33  34 |
| 1. STUDY MANAGEMENT    1. Study management group    2. Study steering committee    3. Public and Patient Involvement       1. PPI co-applicant       2. PPI group    4. Publication and dissemination policy | 34  34  34  35  35  35  35 |
| 1. REFERENCES | 36 |
| 1. APPENDICES    1. ABC Patient Invitation General + Expression of Interest Form    2. ABC Main Study Participant Information Sheet    3. ABC Poster    4. ABC Patient Invitation Leeds + Expression of Interest Form    5. ABC Social Media Post    6. ABC Main Study Consent Form    7. ABC Interview Participant Information Sheet    8. ABC Interview Consent Form    9. ABC Programme Feedback (Rating Scales + Open-ended Questions)    10. ABC Research Process Feedback (Rating Scales + Open-ended Questions)    11. Interview Schedule: Did Not Consent    12. Interview Schedule: Dropped Out Post-Randomisation    13. Interview Schedule: Control Participants Dropped Out During Study    14. Interview Schedule: Intervention Participants Dropped Out During Study    15. Interview Schedule: Control Participants Finished Study    16. Interview Schedule: Intervention Participants Finished Study    17. Demographic and Breast Cancer Information    18. Kessler Psychological Distress Scale    19. Body Appreciation Scale-2    20. Functional Assessment of Cancer Therapy – Breast: Breast Cancer Subscale    21. Hopwood Body Image Scale    22. BREAST-Q: Sexual Well-Being Scale    23. Modified version of the Adult Service Use Schedule: T1    24. Modified version of the Adult Service Use Schedule: T3    25. Modified version of the Adult Service Use Schedule: T4    26. EQ-5D-5L    27. Recovering Quality of Life-Utility Index    28. Work and Social Adjustment Scale    29. Data Management Plan    30. Protocol Amendment History    31. Follow-up ABC Acceptability Questions    32. Additional Demographic Question | 39  41  43  50  51  53  54  56  61  62  67  71  72  73  74  75  76  78  86  87  88  89  90  91  96  101  106  108  109  110  116  118  119 |

ii: **ABBREVIATIONS**

ABC Accepting your Body after Cancer

AD-SUS Adult Service Use Schedule

ANCOVA Analysis of covariance

BAS-2 Body Appreciation Scale-2

BC Breast Cancer

BID Body Image Distress

CBT Cognitive Behavioural Therapy

CI Chief Investigator

CSS Cancer Support Specialist

CTIMP Clinical Trial of Investigational Medicinal Product

DIRUM Database of Instruments for Resource Use Measurement

EoIF Expression of Interest Form

FACT-B Functional Assessment of Cancer Therapy – Breast

GCP Good Clinical Practice

GDPR General Data Protection Regulation

GP General Practitioner

HRA Health Research Authority

ICF Informed Consent Form

K10 Kessler Psychological Distress Scale

NHS R&D National Health Service Research & Development

NICE National Institute for Health and Care Excellence

PIC Participant Identification Centre

PIS Participant Information Sheet

PPI Patient and Public Involvement

QALY Quality Adjusted Life Years

RCT Randomised Controlled Trial

REC Research Ethics Committee

ReQoL-10 Recovering Quality of Life-Utility Index

SIL Study Invite Letter

SOP Standard Operating Procedure

SMG Study Management Group

SSC Study Steering Committee

T1 Time 1 (Baseline)

T2 Time 2 (Post-Intervention)

T3 Time 3 (3-month follow-up)

T4 Time 4 (6-month follow-up)

UWE University of the West of England

WSAS Work and Social Adjustment Scale

# iii: TRIAL SUMMARY

# TITLE: A feasibility study to inform a Randomised Controlled Trial to evaluate ‘Accepting your Body after Cancer’ (ABC), an online-delivered group-based cognitive behavioural therapy (CBT) body image intervention, for women who have received treatment for breast cancer

**OBJECTIVES:** The primary objectives are to 1) establish appropriate, inclusive, and acceptable methods of participant recruitment, retention, and management procedures; 2) establish the feasibility and acceptability of quantitative data collection, including determining appropriate primary and secondary self-report validated outcome measures; and 3) adapt and test a measure of health and social care service use, to inform a future economic evaluation.

The secondary objective is to establish intervention adherence and acceptability of online delivery of the ABC programme among participants and facilitators.

**DESIGN:** A two-arm randomised feasibility study with an embedded qualitative component to further assess feasibility and acceptability.

**SAMPLE SIZE:** We will recruit 120 adult women who have received treatment for a diagnosis of breast cancer and are experiencing body image concerns. Participants will be randomised into intervention and control groups with 60 participants in each arm.

**INCLUSION/EXCLUSION CRITERA:**

Inclusion criteria:

- Identify as a woman.
- 18+ years old.
- Finished active treatment for breast cancer (including chemotherapy, radiotherapy, targeted and immunotherapy) for breast cancer. There is no time limit on when they finished this treatment. Women on endocrine therapy are eligible to take part. Women with metastatic disease are eligible to take part if on endocrine therapy only.
- Completed primary oncological breast cancer surgery with breast conserving surgery or mastectomy with or without immediate definitive breast reconstruction. Women awaiting delayed breast reconstruction, revision or contralateral symmetrisation surgery are eligible to take part provided this surgery is not planned within the duration of the study.
- Recognises that they are experiencing BID as a result of treatment (regarding how the body looks and/or feels).
- Has the capacity to provide informed consent or supported informed consent (e.g., with a family member/friend).
- Has sufficient understanding of English (as the intervention content and measures are currently only available in English).

Exclusion criteria:

- Still undergoing active treatment for breast cancer (e.g., oncological breast surgery including those awaiting the second stage of planned expander/implant reconstruction, chemotherapy, targeted therapies, radiotherapy).
- Undergoing exploration for cancer recurrence.
- Has not received a diagnosis of breast cancer e.g., has had prophylactic treatment for a gene mutation (such as risk-reducing mastectomy).
- Has an eating disorder.
- Unable to provide informed consent.

**INTERVENTION / MAIN STUDY PROCEDURES:** Following consent and completion of baseline self-report questionnaires, participants will be randomised to either the intervention or control arm. The control arm will receive the Macmillan Cancer Support psychoeducational body image booklet (available [here](https://cdn.macmillan.org.uk/dfsmedia/1a6f23537f7f4519bb0cf14c45b2a629/791-source/body-image-mac14192?_ga=2.64651436.2043917443.1713345847-94345358.1713345845&_gl=1*jfi80y*_ga*OTQzNDUzNTguMTcxMzM0NTg0NQ..*_ga_2J203BPENT*MTcxMzM0NTg0NS4xLjEuMTcxMzM0NTg4My4yNC4wLjA.*_fplc*a1lSaVlFa29OS3hDdXUyMzlEbVI3NCUyRm1wdzFDTnlXOGpWbkhKOEdxRXdJcUM3RWxncDZJWG5MVmoycVVtdVkyWUJvYjVJTVBIeTVnb1hZYjloalFycUVnaERXamRlb05SUzRJemgzYkF2OW50c3V5UWlnVDB6MHcwV1NWR0ElM0QlM0Q.)). The intervention arm will receive the Macmillan booklet and the ABC programme, a 7-session, CBT group-based programme delivered online. All participants will complete self-report validated outcome measures at weeks 1 (T1; baseline/pre-intervention), 9 (T2; immediate post-intervention), 20 (T3; three-month post intervention), and 32 (T4; six-month post intervention). Following drop-out or completion of all study procedures, a subset of participants will be invited to take part in interviews.

**OUTCOME MEASURES:** The primary outcome measures are recruitment, retention, and data completion rates, and participant feedback on the research process. The secondary outcome measures are participant and facilitator feedback on the ABC programme, along with responses to validated outcome questionnaires.

# BACKGROUND

# The issue: Body image distress among breast cancer survivors

Breast cancer (BC) is the most common cancer among women in the UK, with 55,500 diagnosed annually (Cancer Research UK, 2022). Fortunately, 85% live at least five years following their diagnosis (Cancer Research UK, 2022), however, they live with treatment-related consequences, including changes to the body, such as breast asymmetry, hair loss/thinning, and fatigue. These changes adversely impact body image (Helms, O'Hea, & Corso, 2008), defined as perceptions, thoughts, feelings, and behaviours, relating to the body’s appearance, functions, and capabilities (Cash & Pruzinsky, 2002).

Systematic reviews identify body image distress (BID) as a salient issue among women treated for BC (Paterson et al, 2016, Davis et al, 2020). This is supported by recent findings from ‘Breast Cancer Now’, who identified 44% of 1,007 BC survivors with body image distress (BID; Breast Cancer Now, 2022); highlighting the scale of the issue. Further, the COVID-19 pandemic has likely heightened BID among this group, as over 1,500 women were facing at least two-year breast reconstruction delays (British Association of Plastic, Reconstructive and Aesthetic Surgeons, 2020, Breast Cancer now, 2022), due to the NHS’ suspension of non-urgent procedures, including reconstruction (Association of Breast Surgery, 2020).

To add further concern, PPI advisors indicate that health professionals lack body image knowledge and/or appropriate signposting, as highlighted by the following quote: “My therapist focused on other aspects of my life and not the trauma that the cancer and treatment had brought on my body”. This is supported by research suggesting that both health professionals and patients will wait for the other to initiate a discussion regarding this topic (Cohen et al 2012). This leaves women feeling that health professionals fail to recognise the severity of impacts on body image and sexuality (McWilliam et al, 2000, Rosman, 2004).

BID is pervasive, with little improvement five years post-treatment (Falk Dahl, 2010). Further, its consequences of anxiety, depression, sexual/intimacy issues, poorer quality of life, and shorter survival (Cousson-Gelie et al.,2007, Begovic-Juhant et al, 2012, Moreira & Canavarro, 2010, Lam et al., 2012), warrant attention, as these psychosocial concerns are higher among UK-based BC survivors versus women with no cancer history (Carreira et al, 2021). This is costly for society, as depression and anxiety among women treated for BC lead to greater healthcare use and costs, and economic losses (Mausbach et al 2020, Van Beek et al 2021).

The above emphasises the need to target potent risk factors for adverse outcomes, including BID. However, we conducted a systematic review of body image interventions for women treated for BC, which revealed a gap in relation to interventions with lasting improvements (Lewis-Smith et al 2018). This highlighted the need to develop an effective body image intervention for this group, and thus informed the development of our intervention, ‘Accepting your Body after Cancer’.

- 1. **The intervention: Accepting your Body After Cancer**

Using the Medical Research Council’s framework (Skivington et al 2021), we employed a step-by-step approach to develop ‘Accepting your Body after Cancer (ABC)’. Following our first systematic review, we conducted a second systematic review of body image interventions for women in midlife, given that most women with BC tend to be in midlife and beyond (Lewis-Smith et al, 2016). This identified a rigorously evaluated and promising group-based CBT intervention for women in midlife, with the largest effects at longest follow-up. Therefore, we examined whether the BID risks targeted in the intervention (e.g., appearance comparisons) were relevant for women treated for BC and found this to be true (Lewis-Smith et al, 2020). Based on these findings, we worked alongside women treated for BC and health professionals to adapt this midlife intervention for women treated for BC (Lewis-Smith, 2017).

Subsequently, we evaluated the feasibility of in-person delivery of ABC among women treated for BC (Lewis-Smith et al 2018). Attrition was low, with 91% completing the intervention, and 80% completing all assessment timepoints. ABC was acceptable, with 94.4% experiencing benefits and advocating for UK-wide dissemination. There were also preliminary significant improvements on body image, self-esteem, avoidance of intimacy, and quality of life (with reductions in distress at follow-up).

Since this publication (Lewis-Smith et al 2018), there have been global requests (e.g., China, UK, Spain) for training to deliver ABC. The CI trained a Canadian cancer hospital, with ABC implemented since 2019. However, the pandemic shifted delivery to online. Nonetheless, impact data indicates that online delivery replicates the preliminary effects identified in our in-person feasibility study (Lewis-Smith et al 2018), suggesting that benefits remain when delivered online. This is supported by feedback: “I don't know how to put it in words, but this program made a difference for me. Something broke inside of me and I feel so much freer now”. Collectively, this provides preliminary support for ABC, when delivered in person and online.

- 1. **Rationale for the study**

Preliminary data indicates that ABC shows promise (Lewis-Smith et al 2018). Online delivery of ABC may overcome barriers, increase geographic accessibility and, and thus, facilitate inclusion of women from diverse backgrounds and reduce health inequalities (Gov.uk, 2022). Such online delivery would also be less costly than in person delivery and will facilitate sustainability of the intervention. Prior to a full-scale RCT to establish the effectiveness of online ABC delivery, a feasibility study is needed to ensure appropriate study design parameters.

# AIMS AND OBJECTIVES

We aim to assess the feasibility and acceptability of conducting an RCT to compare receiving ABC (an online-delivered, CBT, group-based, body image programme) alongside a psychoeducational body image booklet for women treated for breast cancer, with receiving the booklet only. Results will inform the design, management, and future delivery of an evaluation to assess effectiveness and cost-effectiveness in a definitive RCT.

Specific objectives are to:

- Establish appropriate, inclusive, and acceptable methods of participant recruitment, retention, and management procedures.
- Establish the feasibility and acceptability of quantitative data collection, including determining appropriate primary and secondary outcome measures.
- Adapt and test a measure of health and social care service use, to inform a future economic evaluation.
- Establish intervention adherence and acceptability (of online setting) among participants and ABC facilitators.

This study will examine uncertainties that need addressing before a definitive RCT:

- Recruitment processes and response rates: Responses from potential participants invited for randomisation and investigating reasons for declining.
- Intervention adherence and retention within the study: Percentage of participants completing each ABC session, reading the Macmillan booklet (in the control arm), and completing outcome measures at each assessment. This will additionally help calculate the sample size for the proposed follow-on RCT.
- Participants’ experience of randomisation to either the intervention or control arm and their experience of being in these arms.
- Participants’ responses and feedback to self-complete measures. This will determine suitability of the measures for an RCT.
- Adaptation and testing of a measure of health and social care service use: Assessment of comprehensiveness of the service use measure and acceptability of the measure to collect data for a health economic evaluation.

# STUDY DESIGN

The study will be a parallel, two-arm, RCT, with monitoring of recruitment and retention and an embedded qualitative component to assess feasibility and acceptability of the research process and ABC. The end of the study is defined as when all data is collected and finalised.

As this is a feasibility study, the objective is not to power the study enough to detect significant differences, but rather, to provide estimates of parameters to inform a subsequent RCT to evaluate intervention effectiveness. Therefore, a formal a-priori power calculation is not needed. The intention is to recruit N = 120 (60 per arm), with participants individually randomised to either the intervention arm (ABC + Macmillan body image booklet) or control arm (Macmillan body image booklet).

This sample size is based on aiming to have a cost-effective fully powered follow-on substantive or definitive trial and to help quantify the degree of missing data. Data will be used to estimate the upper one-sided 80% confidence interval for the pooled variance of postintervention self-report outcome measures. For an 80% chance of not being underpowered at any level of power, and for any minimum clinically important difference, a sample of 60 per condition would ensure that the percentage error in estimated sample size for a definitive trial would be no more than 11% (i.e., potentially overpowered, but degree of excess restricted to 11%). The estimated sample size for the proposed definitive study can be refined further by estimating the strength of the pre- post- correlation and using these estimates in estimating sample size for repeated measures ANCOVA-styled analyses (controlling for commensurate baseline methods), thus resulting in a cost-effective substantive RCT without compromising reliability of conclusions.

1. **PARTICIPANT ELIGIBILITY CRITERIA**

# Inclusion criteria

- Identify as a woman.
- 18+ years old.
- Finished active treatment for breast cancer (including chemotherapy, radiotherapy, targeted and immunotherapy) for breast cancer. There is no time limit on when they finished this treatment. Women on endocrine therapy are eligible to take part. Women with metastatic disease are eligible to take part if on endocrine therapy only.
- Completed primary oncological breast cancer surgery with breast conserving surgery or mastectomy with or without immediate definitive breast reconstruction. Women awaiting delayed breast reconstruction, revision or contralateral symmetrisation surgery are eligible to take part provided this surgery is not planned within the duration of the study.
- Recognises that they are experiencing BID as a result of treatment (regarding how the body looks and/or feels).
- Has the capacity to provide informed consent or supported informed consent (e.g., with a family member/friend).
- Has sufficient understanding of English (as the intervention content and measures are currently only available in English).
  1. **Exclusion criteria**
- Still undergoing active treatment for breast cancer (e.g., oncological breast surgery including those awaiting the second stage of planned expander/implant reconstruction, chemotherapy, targeted therapies, radiotherapy).
- Undergoing exploration for cancer recurrence.
- Has not received a diagnosis of breast cancer e.g., has had prophylactic treatment for a gene mutation (such as risk-reducing mastectomy).
- Has an eating disorder.
- Unable to provide informed consent.

# STUDY PROCEDURES AND MEASUREMENTS

# Identification and recruitment of participants

We plan to have a phased recruitment, prioritising five geographically diverse NHS Participant Identification Centres (PICS): Bristol, Leeds, Manchester, Nottingham, and Liverpool. The Bristol, Manchester, Nottingham, and Liverpool sites all offer follow-up appointments with women several months after they finish their active treatment for breast cancer. These NHS sites have informed us that it is extremely common for women to bring up feeling uncomfortable with their body at these appointments. Therefore, this opportunity will be used to recruit potentially eligible participants, whereby the nurse will tell them about the study and ask if they would be willing to be sent further information via their postal address (consent to contact). If women consent to being contacted, research nurses will email or post them a Study Invite Letter (SIL; Appendix 1), Participant Information Sheet (PIS; Appendix 2), Expression of Interest Form (EoIF; Appendix 1), and a pre-addressed, stamped envelope (post only). Individuals interested in taking part in the study will be asked to either email, telephone, or return the EoIF to the University-based research team (i.e., not the NHS breast cancer centre). The EoIF requests minimal personal information, depending on the individual’s preferred mode of contact. They will be asked to provide their name in addition to their email address, telephone number, or home address. The research team will then contact the interested individual with further information about the study. For all NHS sites, women who do not contact the research team within two weeks will be telephoned by the hospital research nurse about the study. However, they will not be contacted again after this. A poster (Appendix 3) about the study will also be displayed in the waiting rooms for all these sites, so that interested women can either scan the QR code or enter the URL which will take them to the PIS. They can also telephone or email the research team (details on the poster) who can send them the PIS and EoIF.

The Leeds site does not have such follow-up appointments. Therefore, the clinical care team will review the medical records of their patients and identify women who have recently finished active treatment. To encourage diversity of participants, they will try to identify women across different ages and ethnicity. They will initially contact the individuals to ask if they are interested in finding out more about the study. If they are, and consent to have their contact details passed onto the research nurse (at the hospital), they will send out the SIL (Appendix 4), PIS (Appendix 2), EoIF (Appendix 4), and a pre-addressed, stamped envelope to the individual. Similarly, individuals interested in taking part in the study will be asked to either email, telephone, or return the EoIF to the research team. The research team will then contact the interested individual with further information about the study. For individuals who choose not to take part, the EoIF includes a question asking them to provide a brief reason why they made this decision. This may help inform the design and planning of a future definitive RCT.

To encourage greater diversity of participants, we will additionally advertise the study via various UK cancer support organisations, including Breast Cancer Now, Maggie’s, Keeping Abreast, and Flat Friends. This will hopefully capture women who are years beyond treatment and are no longer having contact with their hospital yet are struggling with BID. To engage women from underserved groups, we will also advertise the study via OUTpatients (supporting LGBTQIA+ individuals who have had cancer) and Black Women Rising (supporting women of colour who have had cancer). These organisations will advertise the study through their respective communication channels and social media platforms using a social media image (Appendix 5) like that of the poster, with Maggie’s displaying the physical posters (Appendix 3) in their centres. Interested women can either scan the QR code or enter the weblink which will take them to the PIS (Appendix 2) They can also telephone or email the research team (details on the poster) who can send them the PIS and EoIF.

If the above recruitment avenues do not result in obtaining the sample of 120 women, we will use social media as an additional option for recruitment. First, we will advertise the study via the research team’s respective university and professional social media channels (e.g., the PI’s university has over 90k followers across platforms). This will include posting a social media image (Appendix 5) like that of the poster about the study. Interested women can either scan the QR code or enter the weblink which will take them to the PIS (Appendix 2). They can also telephone or email the research team (details in the image) who can send them the PIS. If this fails to recruit the number of women needed, the last option will involve asking UK-based social media content creators who have had breast cancer (i.e., women from the general public who share their experience of breast cancer with a large group of followers) to advertise the research (e.g., @the_boob_battle has 20k Instagram followers). This will include posting the social media image about the study. Interested women can either scan the QR code or enter the weblink which will take them to the PIS and EoIF. They can also telephone or email the research team (details in the image) who can send them the PIS and EoIF. Having used these social media approaches previously, we were able to engage younger women who have had breast cancer (e.g., 20s/30s). This would improve participant diversity in terms of age.

- 1. **Screening**

Irrespective of their recruitment avenue, once interested women have contacted the research team, screening will be completed by the research team over the telephone. During this conversation, special attention will be paid to the eligibility criteria and participants will be asked to confirm that they meet these. The researcher will also go through the PIS (Appendix 2), answer any questions that the potential participants have, and discuss whether the study is right for them. The researcher will have a list of support organisations to signpost the potential participant to if needed (these are also included on the PIS) and will recommend that highly distressed individuals speak to their GP or Breast Cancer Nurse (as appropriate).

- 1. **Informed consent**

Following screening, eligible women will be required to complete an informed consent form (ICF; Appendix 6). They will be given the option of completing this online via the secure survey software Qualtrics or via a paper version of the form. If they prefer the first option, they will be sent a weblink to the online ICF. Once completed, they will be emailed a signed copy of the ICF for their records. If they prefer the second option, they will be mailed the ICF and a pre-addressed, stamped envelope to return the signed ICF via the post.

Some of these participants will be purposefully sampled to take part in an interview to provide feedback about the research process or ABC. They will be required to read a separate PIS (Appendix 7) and complete a separate ICF (Appendix 8). Similarly, they will be given the option of completing the ICF online via the survey software Qualtrics or via a paper version of the form. If they prefer to complete the ICF online, they will be sent a weblink to the online ICF. Once completed, they will be emailed a signed copy of the ICF for their records. If they prefer to complete a paper version of the ICF, they will be mailed the ICF and a pre-addressed, stamped envelope to return the signed ICF via the post.

- 1. **Randomisation**

Once participants have provided informed consent, and completed baseline data collection (Week 1), they will be randomised to either the intervention or control arm using Sealed Envelope, a web-based randomisation system. Randomisation (at the individual level) will be independent and concealed, using permuted block randomisation. The system will send an email to the Study Manager and CI outlining allocated arms for each participant. The allocation will be made known to the participant via their preferred form of contact (post, email, or telephone).

- 1. **Blinding**

Participants will not be blinded to their condition due to the nature of the psychological intervention. The Study Manager and CI will not be blinded to treatment condition, due to an aim of the study being to collect qualitative feedback from participants about their experience of their respective condition. However, the Statistician and Health Economist will be blinded to treatment condition, to minimise any potential bias influencing data analyses.

- 1. **Data Collection**

We will collect a variety of different type of data exploring acceptability and feasibility. This will inform the design of a future definitive RCT and help refine intervention, recruitment, and retention procedures. Figure 1 provides an outline of participant flow in the study and Table 1 provides an outline of the different assessments and their associated timepoints.

- - 1. **Quantitative measures of feasibility and acceptability**

We will collect the following quantitative data to assess feasibility and acceptability of the research design and ABC:

- - - 1. **Monitoring data**
- Rates of recruitment (and associated response rates), with attention to the method by which women were recruited and their demographic diversity.
- Rates of retention following randomisation:
  - Proportion completing ABC (number of sessions attended and between-session activities completed)
  - Proportion of control arm reading the Macmillan body image booklet (plus percentage of booklet read)
  - Proportion completing outcome measures at each assessment (plus data completeness rates; outcome measures described in 5.6.3).
    - 1. **Self-report rating scales included in outcome assessments (see 5.6.3)**
- Rating scales to explore acceptability of ABC (e.g., the group format, online nature, between-session activities; Appendix 9) - to be completed by ABC participants in the immediate post-intervention assessment, i.e., Week 9.
- A single rating scale examining how helpful participants found the ABC programme will also be included in the three- and six-month follow up assessments (see Appendix 31). This will allow monitoring of how participants’ perceptions of the programme and its impacts may develop over time.
- Rating scales to explore acceptability of the research process generally (e.g., information provided about the study, randomisation process, questionnaires; Appendix 10) – to be completed by all participants in the six-month post-intervention assessment i.e., Week 32.
  - 1. **Qualitative measures of feasibility and acceptability**

In addition to the above, we will collect the following qualitative data to assess feasibility and acceptability of the research design and ABC:

- - - 1. **Open-ended questions included in outcome assessments (see 5.6.3)**
- Open-ended questions to explore acceptability of ABC (e.g., the group format, online nature, between-session activities; Appendix 9) - to be completed by ABC participants in the immediate post-intervention assessment, i.e., Week 9.
- A single open-ended question examining how helpful participants found the ABC programme will also be included in the three- and six-month follow up assessments for (see Appendix 31). This will allow monitoring of how participants’ perceptions of the programme and its impacts may develop over time.
- Open-ended questions to explore acceptability of the research process generally (e.g., information provided about the study, randomisation process, questionnaires; Appendix 10) - to be completed by all participants in the six-month post-intervention assessment i.e., Week 32.

Figure 1: Participant flow through the study


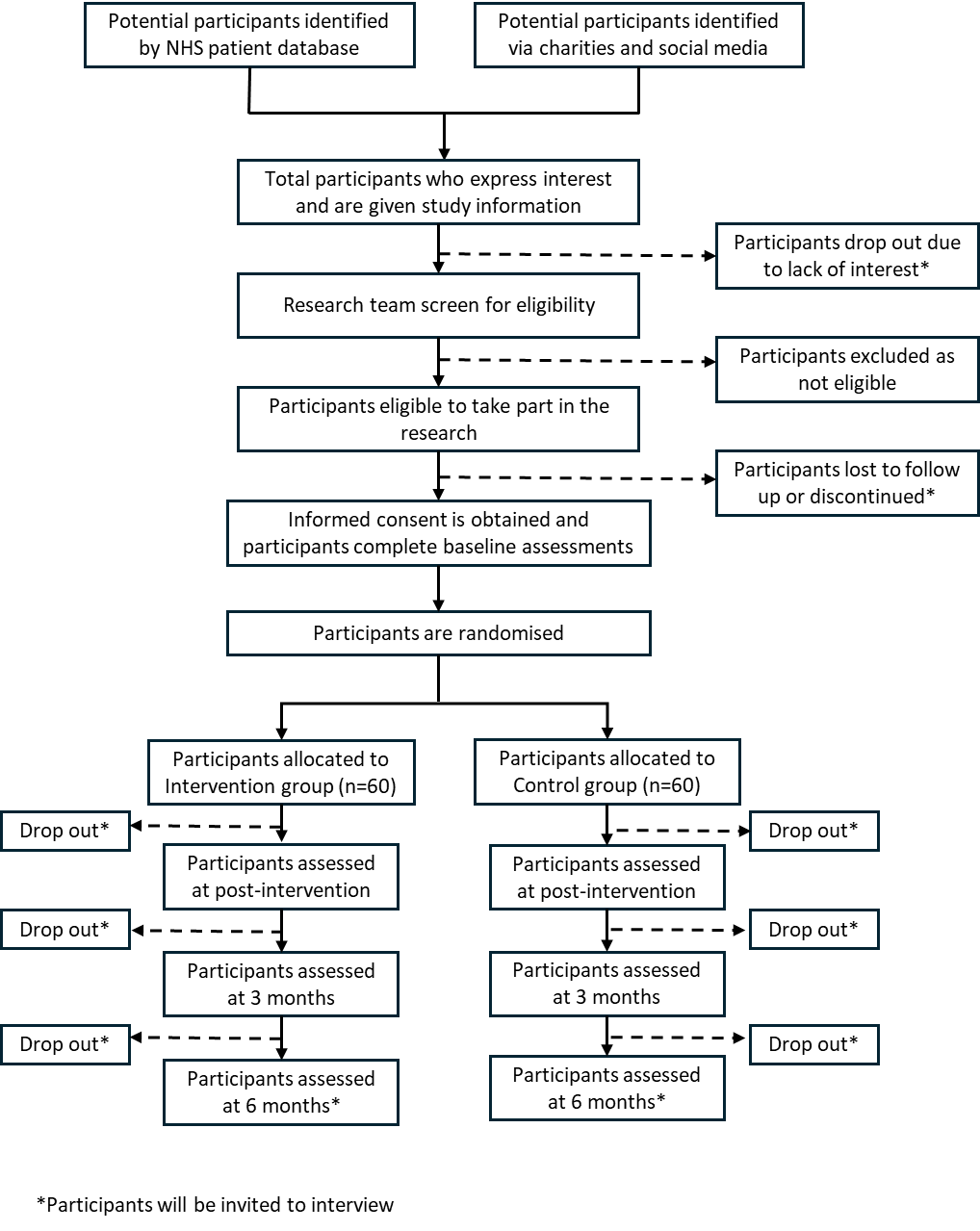


Table 1: Schedule of Assessments

|  | **Baseline**  **(T1)** | **Immediate post-intervention**  **(T2)** | **3-month post-intervention (T3)** | **6-month post-intervention (T4)** |
| --- | --- | --- | --- | --- |
|  | **Week 1** | **Week 9** | **Week 20** | **Week 32** |
| **Consent and general information** |  |  |  |  |
| *Consent* | X |  |  |  |
| *Demographics* | X |  |  |  |
| *Additional Demographic Question* |  |  | X |  |
| *Cancer-related information* | X |  |  |  |
| **Self-reported primary validated outcome measures** |  |  |  |  |
| *Kessler Psychological Distress Scale (K10)* | X | X | X | X |
| *Body Appreciation Scale-2 (BAS-2)* | X | X | X | X |
| *Functional Assessment of Cancer Therapy – Breast (FACT-B Version 4): Breast Cancer Subscale* | X | X | X | X |
| **Self-reported secondary validated outcome measures** |  |  |  |  |
| *Hopwood Body Image Scale* | X | X | X | X |
| *BREAST-Q: Sexual Well-Being Scale* | X | X | X | X |
| **Health economic measures** |  |  |  |  |
| *Modified version of the Adult Service Use Schedule (AD-SUS)* | X |  | X | X |
| *EQ-5D-5L* | X |  | X | X |
| *Recovering Quality of Life-Utility Index (ReQoL-10)* | X |  | X | X |
| *Work and Social Adjustment Scale (WSAS)* | X |  | X | X |
| **Acceptability assessments** |  |  |  |  |
| *ABC Acceptability Rating Scales* |  | X |  |  |
| *ABC Acceptability Open-Ended Questions* |  | X |  |  |
| *Follow-up ABC Acceptability questions* |  |  | X | X |
| *Research Process Acceptability Ratings Scales* |  |  |  | X |
| *Research Process Acceptability Open-Ended Questions* |  |  |  | X |
| **Interviews** | As appropriate | | | |

- - - 1. **Interviews**

Purposive sampling will be used to recruit a subset of 16-20 study participants to participate in semi-structured interviews. This approach will be used to explore the widest range of perspectives possible, ensuring that we have qualitative data from participants of different ages, ethnicities, sexual orientations, geographical locations, and socio-economic backgrounds.

These interviews will explore the experiences of participants from both conditions in relation to recruitment, randomisation, the Macmillan body image booklet, communication from the research team throughout the study, and completion of outcome measures. Participants from the intervention arm will also be asked about the acceptability of the ABC intervention.

We will target participants at different stages of the study and who may have had different experiences, which will enable us to identify barriers and solutions to participation and retention. This will include women who considered taking part at the beginning (i.e., they read the PIS) but decided not to proceed (Appendix 11), participants who dropped out immediately following randomisation (Appendix 12), participants who were randomised to the control arm but dropped out before the last questionnaire (see Appendix 13), participants who were randomised to the intervention arm but dropped out before the last questionnaire (see Appendix 14), participants who were randomised to the control arm and completed the whole study (i.e., completed the final assessment; Appendix 15), and participants who were randomised to the intervention arm and completed the whole study (i.e., completed the final assessment; see Appendix 16). We will endeavour to have an approximately equal number of participants reflecting each circumstance.

These interviews will occur across 12 months, with the sampling, data collection, and analysis happening concurrently and iteratively. All ABC facilitators will also be interviewed following intervention delivery to explore their experience of the programme, as well as their perceived barriers to participant retention, and solutions, if necessary.

- - 1. **Self-report validated outcome measures**

Data relating to self-report validated outcome measures will be collected to determine the suitability of these measures for use in a subsequent definitive RCT, rather than to draw conclusions about the relative efficacy of ABC.

Participants will be asked to complete self-report outcome assessments at four timepoints:

- T1: Pre-intervention (i.e., baseline; Week 1)
- T2: Immediate post-intervention (within one week of finishing the last ABC session; Week 9)
- T3: Three-month post-intervention (Week 20)
- T4: Six-month post-intervention (Week 32).

However, the specific data that will be collected may differ at each timepoint. These will be clarified in the following sections.

As per the participant preferences, this assessment will be completed either online (via Qualtrics) or by paper (the participant will be sent the questionnaire and a pre-paid and addressed envelope to return).

- - - 1. **Demographics and cancer-related information**

We will collect contextual information about women participating in the study at pre-intervention (i.e., baseline; Week 1).

This will include information about their demographics (e.g., age, ethnicity, relationship status), as well as their cancer diagnosis and treatment (e.g., stage of cancer, time since finishing active treatment, modes of treatment received; Appendix 17).

Additional demographics questions will be included in the three-month follow up questionnaire to ensure that all relevant information is collected (see Appendix 32).

- - - 1. **Primary validated outcome measures**

The proposed primary outcome measures undergoing consideration for a subsequent RCT will be assessed at all four timepoints, and are as follows:

- Kessler Psychological Distress Scale (K10; Kessler et al., 2002; Appendix 18)

The K10 is a measure of non-specific psychological distress. Participants are asked to rate their emotional states on a 5-point scale over 10 items.

- Body Appreciation Scale-2 (BAS-2; Tylka & Wood-Barcalow, 2015; Appendix 19)

The BAS-2 is a measure of a person’s acceptance and/or favourable opinions towards their body. Participants are asked to rate each of the 13 items on a 5-point scale.

- Functional Assessment of Cancer Therapy – Breast (FACT-B Version 4): Breast Cancer Subscale (Brady et al., 1997; Appendix 20)

The FACT-B is a measure of health-related quality of life for breast cancer patients and includes five subscales. We will be using the Breast Cancer Subscale, whereby participants rate each of the 10 items on a 5-point scale.

- - - 1. **Secondary validated outcome measures**

The proposed secondary outcome measures undergoing consideration for a subsequent RCT will be assessed at all four timepoints, and are as follows:

- Hopwood Body Image Scale (Hopwood et al., 2001; Appendix 21)

A 10-item scale measuring body image in cancer patients. The scale includes measurement of affective, behavioural, and cognitive elements of body image. Participants rate each item on a 4-point scale.

- BREAST-Q: Sexual Well-Being Scale (Pusic et al., 2009; Appendix 22)

The BREAST-Q is a measure of quality of life and patient satisfaction comprising six subscales, of which we will use only the sexual well-being subscale. This six-item subscale asks participants to rate their feelings related to their sexual attractiveness, sexual confidence, and comfort during sex on 5-point scale

- - 1. **Health economic measures**

Measures for the economic evaluation include a measure of service use for the estimation of costs and two preference-based, generic measures of health-related quality of life as potential candidates for the estimation of quality adjusted life years (QALYs) suitable for cost-utility analysis in a definitive RCT. In addition, we have included a measure of functional impairment that may be suitable for cost-effectiveness analysis. The measures will all be completed by participants at baseline (T1; Week 1), three-month post-intervention (T3; Week 20), and six-month post-intervention (T4; Week 32) assessment points:

- Modified version of the Adult Service Use Schedule (AD-SUS; see Appendix 23 for T1 measure, Appendix 24 for T3 measure, Appendix 25 for T4 measure)

We have adapted a version of the AD-SUS that has been successfully used in depression-focused studies (e.g., Strauss et al, 2020, Richards et al 2016). The measure takes an NHS and social services perspective and has been adapted to ensure coverage of services relevant to women treated for breast cancer through a literature review (existing economic evaluations focused on this population), review of cancer-focused service use measures in the Database of Instruments for Resource Use Measurement (DIRUM; https://www.dirum.org/) and discussions with the team. At baseline, the ABC AD-SUS covers the last 3 months and at follow-up, the measure covers the period since the last assessment. We will test and further adapt the ABC AD-SUS to support the estimation of the cost of services used by participants in a future definitive RCT.

- EQ-5D-5L (Herdman et al., 2011; Appendix 26)

The EQ-5D-5L is a standardised measure of health status, that assesses the respondent’s health-related quality of life across five dimensions, including mobility, self-care, usual activities, pain/discomfort, and anxiety/depression. It also includes a visual analogue scale to assess the respondent’s overall current health.

- Recovering Quality of Life-Utility Index (ReQoL-10; Keetharuth et al., 2018, 2021; Appendix 27)

Whilst the EQ-5D-5L is preferred by NICE, PPI advisors raised concerns that this measure may not adequately capture the psychological aspects of BID. Thus, the ReQoL has been added for comparison, as it has a broader focus on psychological wellbeing, having been developed for application to mental health populations. The 10-item version of the ReQoL will be used, from which the ReQoL Utility Index (ReQoL-UI) can be generated.

- Work and Social Adjustment Scale (WSAS; Mundt et al., 2002; Appendix 28)

In addition, the WSAS has been added for comparison, as it assesses the impact of a person’s psychological wellbeing on their ability to function in terms of work, home management, social leisure, private leisure and personal or family relationships. The PPI group felt that this measure was relevant in terms of impacts of their BID on different domains of their life. The 5-item measure is summed to produce a score indicating whether low, moderate, or high work and social impairment.

- 1. **Gift Voucher**

Once participants have completed the final assessment (T4, Week 32) , they will be emailed a shopping gift voucher of £15 to thank them for taking part.

# INTERVENTION AND CONTROL

- 1. **ABC intervention**

ABC comprises seven 2-hour group sessions (with approximately 8 women per group) delivered online via Microsoft Teams and across 7 consecutive weeks. The intervention aims to improve body image among women treated for BC. Rooted in CBT, ABC uses strategies to alter unhelpful thoughts, reduce anxiety, and promote non-avoidant behaviours. Other topics are also explored, including sociocultural pressures for women, intimacy, physical activity, self-care, mindfulness, and relaxation. The sessions will be guided using PowerPoint slides, which will include text, images, and videos. Each session will include individual and group-based activities, and participants will be asked to complete between-session readings and activities. Table 2 provides an overview of ABC and its content.

Upon randomisation to the intervention arm, participants will be sent the Macmillan body image booklet (described in 6.3) and informed of the next available date to begin ABC. To prepare participants, they will be sent (via the post or email, as per the participant’s preference) a guide concerning session expectations (e.g., format, activities), in addition to clear instructions on how to use Microsoft Teams. Further, they will be sent all intervention materials. Any participants who do not have access to the internet (or have limited data use) will be provided with a data internet card.

A week prior to the first ABC session, participants will be asked to read the pre-session intervention materials (i.e., Session 1: Part 1) to help them start reflecting on their goals. In the session, there will be introductions and discussion of ground rules before the structured session content is delivered. All participants will be asked to have the second part of the intervention materials for the session (i.e., Session 1: Part 2) in front of them, to facilitate individual and group exercises. At the end, they will also be asked to complete two between-session activities at home (e.g., mirror exposure exercise) before the next session. They will also be advised to read the pre-session intervention materials (i.e., Session 2: Part 1) for the following week’s session. The same format will continue for all sessions (approximately 21 pages/session). This format (including intervention materials, between-session activities) has received positive feedback from study participants and PPI advisors.

Table 2: ABC content overview

| **Session** | **Content** |
| --- | --- |
| 1 | - Introduction to body image - Personal reflection upon the impact of body image concerns - Exploration of personal goals |
| 2 | - Introduction to the CBT approach - Physiological symptoms of anxiety - Exploration of body image and self-esteem - Relaxation training |
| 3 | - Stopping negative body-related self-talk - Developing alternative, balanced thoughts - Planning a self-care activity schedule - Relationship between body function and movement |
| 4 | - Sociocultural pressures for women in midlife - Internalisation of the youthful-thin ideal - Body comparisons – experimental activity - Body nurture with accepting self-talk |
| 5 | - Exploration of relationships and intimacy - Managing people’s reactions - Cognitive restructuring process - Physical activity and movement |
| 6 | - Identifying core beliefs - Modifying mistaken beliefs - Engaging the senses – mindful eating - Relaxation exercise |
| 7 | - Positive body affirmations - Reducing the chances of a setback - Dealing with a setback - Future plans |

- 1. **Intervention facilitators and fidelity**

Based on 60 women being in the intervention arm, and approximately 8 women per group, there will be approximately 6-8 ABC groups, running in both parallel and sequentially. Each group will be delivered jointly by two Cancer Support Specialist (CSS) (e.g. a Psychologist a nurse) from a Maggie’s centre. However, there will be a different pair of facilitators across each group, with some running only one group and others running up to three (depending on the capacity).

Working for a cancer support service, facilitators already have knowledge and skills to support people treated for breast cancer. Their expertise also complements one another, as the psychologist has undergone psychotherapeutic training (and thus can confidently deliver the CBT elements), whilst the CSS understands the treatment experience and impacts (e.g., on body functionality, intimacy), and thus can deliver the other intervention elements. All facilitators will receive two days of online training from the PI, who has considerable experience in training facilitators to deliver body image interventions (including ABC). They will be provided with materials and encouraged to make notes during the training to help support them during deliver of the ABC sessions.

Intervention fidelity will be assessed across different ABC groups and sessions. All ABC sessions will be recorded, and a selection (50%) will be assessed by the research team based on perceived competency (percentage reflecting how well facilitators delivered the sessions) and intervention adherence (percentage reflecting the extent to which facilitators completed each section of the sessions).

- 1. **Control arm**

Discussions with PPI advisors indicated that some form of body image-specific support should be provided for the control arm. Macmillan’s freely available psychoeducational body image booklet was considered appropriate, given that the thorough 77-page booklet provides a substantial amount of support and guidance relating to managing body image concerns. It explains the effects of cancer on body image, and provides practical guidance (e.g., make-up) and psychoeducational guidance (e.g., managing others’ reactions), in addition to some CBT strategies. Further, given that there is no current ‘treatment-as-usual’ in relation to body image support for women treated for breast cancer, this booklet may constitute the first time that these women have received support specifically relating to body image, and may even reduce distress. Upon randomisation to the control arm, participants will be sent the Macmillan body image booklet and encouraged to work through it gradually. The booklet can be accessed via the following webpage: <https://cdn.macmillan.org.uk/dfsmedia/1a6f23537f7f4519bb0cf14c45b2a629/791-source/body-image-mac14192>

1. **DATA ANALYSIS**
   1. **Primary aims**

The primary aims of the feasibility study will be delivered by conducting descriptive analyses of monitoring data (see 5.6.1.1) and self-report rating scales (see 5.6.1.2), as well as qualitative analyses of open-ended questions (see 5.6.2.1) and interviews (see 5.6.2.2). This will help indicate feasibility and acceptability of the whole research process and the intervention, which will help inform a definitive RCT.

- - 1. **Analysis of quantitative data**
       1. **Monitoring data**

Descriptive analyses (i.e., percentages and confidence intervals) will be conducted on numbers relating to recruitment (including numbers of eligible women who decide not to proceed after reading the PIS) and retention following randomisation, including numbers who complete ABC (i.e., number of sessions attended and between-session activities completed), who read the Macmillan body image booklet (in the control arm; including percentage of booklet read), and who complete self-report outcome measures at each assessment timepoint (including data completeness rates). We will assess the extent of missing data, withdrawals and loss-to-follow-up.

We will report data in line with the Consolidated Standards of Reporting Trials (CONSORT) 2010 Statement showing attrition rates and loss to follow-up.

- - - 1. **Self-report rating scales**

Descriptive analyses (i.e., means, standard deviations, percentages) will be conducted on the ratings scales related to acceptability of ABC (e.g., the group format, online nature, between-session activities; Appendix 9), as well as those related to acceptability of the research process generally (e.g., information provided about the study, randomisation process, questionnaires; Appendix 10).

- - 1. **Analysis of qualitative data**
       1. **Open-ended questions**

The responses to the open-ended questions asking for feedback on ABC (e.g., the group format, online nature, between-session activities; Appendix 9) and the research process generally (e.g., information provided about the study, randomisation process, questionnaires’ Appendix 10) will be analysed in NVivo using thematic analysis (Braun & Clarke 2006). The analytic process of ‘codebook’ thematic analysis will be used (Braun & Clarke 2006, 2022), whereby text will be coded using a priori codes and themes relating to feasibility and acceptability (informed by O’Cathain et al 2015).

- - - 1. **Interviews**

Interviews will be digitally recorded, transcribed verbatim, and analysed in NVivo using thematic analysis (Braun & Clarke 2006). The analytic process of ‘codebook’ thematic analysis will be used (Braun & Clarke 2006, 2022), whereby transcripts will be coded using a priori codes and themes relating to feasibility and acceptability (informed by O’Cathain et al 2015).

- 1. **Analysis of self-report outcome measures**

The self-report outcome measures (K10, BAS-2, FACT-B: Breast Cancer Subscale, Hopwood Body Image Scale, and BREAST-Q: Sexual Well-Being Scale) are each well-established validated measures which will be scored according to the most appropriate guidelines. The design (pre- post- two arm parallel randomised controlled trial) and the selected measures are amenable to analysis using long-established statistical techniques. The analysis will proceed using the intention-to-treat analysis set with the statistician blinded to allocation. These analyses include:

- Descriptive statistics (including mean and standard deviation) by randomised arm at each time point (T1, T2, T3, and T4).
- Unadjusted statistical comparison between arms at each timepoint (T1, T2, T3, and T4) using the independent samples *t*-test with 95% and 80% confidence intervals and quantification of effect size using Cohen’s *d*. If statistical assumptions do not seem tenable, robust non-parametric bootstrap equivalent tests will be used.
- Adjusted statistical comparison between arms at each timepoint (T2, T3, T4) will be conducted controlling for commensurate baseline measures using ANCOVA (Analysis of Covariance) along with 95% and 80% covariate adjusted confidence intervals and quantification of effect size using partial eta -squared. If statistical assumptions do not seem tenable, robust remedies will be considered, including a baseline by randomised arm interaction effect, or homoscedasticity corrected robust standard errors (HC3) or the robust non-parametric bootstrap equivalent ANCOVA.
  1. **Heath economic evaluation**

The draft ABC AD-SUS (Appendices 23-25) will undergo testing for the following:

- Acceptability: This will be assessed via the proportion of participants completing the measure, percentage of missing items, and consideration of the clarity of any questions associated with missing data.
- Comprehensiveness: This will be assessed via open-ended questions included in the AD-SUS asking participants to report any ‘other’ services not reported elsewhere in the measure (missing items).
- Redundancy: This will be assessed through consideration of any service items included in the AD-SUS but not reported as being used by any participant (redundant items).

These results, plus feedback from participants, will inform a final version of the AD-SUS for a future definitive trial.

Acceptability of the EQ-5D-5L (Appendix 26), ReQoL-10 (Appendix 27), and WSAS (Appendix 28) will be assessed using completion rates (proportion of participants completing each of the measures and percentage of missing items within each of the measures), plus feedback from participants. Service use, health-related quality of life and the WSAS will be summarised and reported descriptively.

# DATA MANAGEMENT

See Appendix 29 for the complete data management plan.

## **Data collection**

All data will be handled and stored securely and in compliance with the Data Protection Act and GDPR.

Personal information relating to contact details will be collected when participants provide informed consent to participate in the full study. Participants will be identified by a unique participant ID, and files linking participant ID to their contact details will be held on the University of the West of England OneDrive for Business, which is compliant with GDPR, ISO27001 and Cyber Essentials.

## Participants will complete several self-report quantitative and qualitative measures at different assessment time points throughout the study (see 5.6 and Table 1). This data will be collected online via Qualtrics (whereby participants are sent the appropriate weblink), a secure online survey platform. Qualtrics uses Transport Layer Security (TLS) encryption (also known as HTTPS) for all transmitted data. This data will be downloaded into SPSS (a statistical software package) and NVivo (a qualitative data analysis software package) with files held on OneDrive for Business.

## If participants complete these measures by paper, they will be sent these in the post with a stamped pre-addressed envelope to return to the research team. Once returned, these will be stored in a locked filing cabinet in a locked office at the University of the West of England. Once the data is inputted directly into SPSS and NVivo files on OneDrive for Business, the paper versions will be securely destroyed.

Anonymised data related to ABC attendance and engagement, as well as recordings of the sessions, will be collected by programme facilitators. This data will be shared with the research team via OneDrive for Business, which will then be copied over onto the University of the West of England OneDrive for Business.

Participants taking part in the recorded interviews will be given a pseudonym, and thus will not be identifiable. The recordings via Teams will be saved into a file on the University of the West of England OneDrive for Business, and once transcribed, they will be deleted.

Other quantitative data collected throughout the trial (e.g., withdrawal rates) will be collected by the research team and saved in a file on OneDrive for Business.

## **Access to Data**

Personal information relating to participant contact details, and files linking participant ID to their contact details, will only be accessed by the Study Manager and CI.

All other data files will only be accessed by the research team, with any sharing of data files between the team completed via password-protected university OneDrive files.

Direct access will be granted to authorised representatives from the Sponsor, host institution and the regulatory authorities to permit trial-related monitoring, audits, and inspections- in line with participant consent.

- 1. Archiving

All study documentation will be archived for a minimum of 10 years following the end of the study at the University of the West of England.

# ETHICAL AND REGULATORY CONSIDERATIONS

# HRA Approval

Prior to the enrolment of participants, the Health Research Authority (HRA) and NHS Research Ethics Committee (REC) must provide written approval of the conduct of the study, the protocol, all participant-facing materials, study advertisements, and participant compensation. (IRAS: 327507, REC ref: 24/NE/0092, HRA approval date: 05/06/2024)

- 1. **University REC Approval**

Following HRA approvals, the study will also be approved by the appropriate REC within the University of the West of England. (Approval date: 20/06/2024, Project Number: CHSS.24.06.222)

- 1. **Amendments**
     1. **Substantial Amendments**

As outlined in the HRA Non-CTIMP Standard Conditions, a substantial amendment is any amendment to the terms of the application for ethical review, or to the protocol or other supporting documentation approved by the REC that is likely to affect to a significant degree:

(a) the safety or physical or mental integrity of the research participants

(b) the scientific value of the research

(c) the conduct or management of the research, including its ongoing legality and feasibility.

Any substantial amendment will be submitted for approval through IRAS. Once the substantial amendment has been approved by NHS REC, the CI will inform the University REC of the amendment. The substantial amendment will not be implemented until approval is granted by both NHS and University REC.

Updated versions of the protocol and any other documents will be recorded in the version control table in Appendix 30.

- - 1. **Minor Amendments**

Minor amendments do not require NHS REC approval. Such amendments will be submitted to the University of the West of England REC for approval prior to being implemented.

- 1. **Good Clinical Practice**

The study will be conducted in accordance with the principles of Good Clinical Practice (GCP). Members of the study team who have contact with participants will complete the full GCP course prior to the start of the study and complete a refresher every two years as needed.

- 1. **Protocol Violations and Serious Breaches**

All deviations or violations from the protocol will be reported to the Chief Investigator who will report such incidents to the trial management and steering committees. The trial management and steering committees will assess whether amendments or other action (e.g. further training for research staff) is needed to address such protocol deviations or violations.

In accordance with the HRA Non-CTIMP Standard Conditions, a serious breach is defined as a breach of the protocol or, of the principles of Good Clinical Practice which is likely to affect to a significant degree the safety or physical or mental integrity of the research participants, or the scientific value of the research. The Sponsor will be notified within 24 hours of identifying a likely Serious Breach. If a decision is made that the incident constitutes a Serious Breach, the incident and agreed action plan will be reported to the NHS REC.

- 1. Financial and other competing interests

The Chief Investigator, study management group members, steering committee members, and other individuals involved in the study have no financial or competing interests regarding the conduct, results, or outcomes of this study.

- 1. Insurance indemnity

The conduct of this study is covered by the insurance arrangements of the University of the West of England, which includes Employers Liability Insurance and Public Liability insurance.

- 1. **Care during and following the study**

Control participants will be given the 77-page Macmillan body image booklet, which provides a substantial amount of support and guidance relating to managing body image concerns. Further, given that there is no current ‘treatment-as-usual’ in relation to body image support for women treated for breast cancer, this booklet may constitute the first time that these women have received support specifically relating to body image, and may even reduce distress.

Intervention participants may experience distress during the sessions, given the sensitive nature of the content relating to body image and its impacts (e.g., on relationships). However, any distress will be managed by the ABC facilitators, who have a great deal of experience in supporting women treated for breast cancer.

Completing the self-report outcome measures may evoke distress for participants, due to the sensitive nature of the items (e.g., relating to body image and sexual wellbeing). However, this risk is outlined in the PIS (Appendix 2), which also includes signposting to various cancer support organisations and general mental health support organisations. Participants will also be reminded that they can stop completing the questionnaires at any point or skip any questions. Further, PPI advisors reviewed, reduced, and refined the number of provisional outcome measures. They also felt that the measures and comprising items were appropriate and reflected the impact of their body image concerns. Therefore, whilst these items might evoke distress among participants, this is outweighed by the potential for the research to indicate evidence that ABC improves body image distress and associated impacts.

A subsample of participants will be interviewed at different points throughout the study, to explore their experience and feedback on all aspects relating to the research process (e.g., the recruitment process, randomisation). Whilst these interviews (Appendices 11-16) are not focused on the participants’ own body image, there is still the risk of distress. However, this risk is outlined in the PIS (Appendix 2), which also includes signposting to various cancer support organisations and general mental health support organisations. Participants will also be reminded that they do not have to answer any questions they do not feel comfortable answering and that they can stop the interview at any time. A distress protocol will be followed, whereby the interview will be paused and subsequently terminated if required.

For participants in the intervention arm, the final session of ABC covers managing future setbacks relating to body image, thereby equipping participants with self-management tools they can use once the study ends. All participants (including intervention and control) will be provided with the Macmillan body image booklet and will be signposted to appropriate support at all data collection timepoints.

- 1. **End of Study**

For safety reporting and regulatory purposes, the end of the study will be when all data collection is complete, and data is finalised (e.g. transcripts complete). The REC will be informed about the end of the study within 90 days of the final follow-up visit taking place.

1. **STUDY MANAGEMENT**
   1. **Study Management Group**

The Study Management Group (SMG) comprises Dr Helena Lewis-Smith (CI), Dr Abigail Jones (Study Manager), Professor Diana Harcourt, Patricia Fairbrother, Professor Paul White, Professor Sarah Byford, and Professor Shelley Potter. The Study Management Group will meet monthly to discuss the progress and management of the project, whilst Dr Helena Lewis-Smith and Dr Abigail Jones will be responsible for the day-to-day management of the study.

- 1. **Study Steering Committee**

The Study Steering Committee (SSC) comprises the SMG and the following seven independent members:

- Professor Fiona Cramp (Independent Chair)
- Julie Wolfarth and Sian Hallewell (Breast Cancer Survivors)
- Dr Charlotte George (Clinical Psychologist)
- Professor Claire Foster (Psycho-Oncology Researcher)
- Maxine McCoy (Associate Director of Services and Service Improvement – Breast Cancer Now) and
- Claire Taylor (Chief Nursing Officer – Macmillan Cancer Support)

The SSC will meet up to three times a year and are responsible for the overall oversight of the project. This includes ensuring that the study is conducted to rigorous ethical and scientific standards, that the protocol is followed, and is on schedule.

- 1. **Public and Patient Involvement (PPI)**
     1. **PPI co-applicant**

The study has a PPI lead and co-applicant (Patricia Fairbrother). She has been extensively involved in the development and refinement of the study proposal. In the study, she will be responsible for co-chairing the PPI group and will attend all SMG meetings.

- - 1. **PPI group**

There has been PPI involvement throughout every stage of the development of ABC and its preliminary evaluation. Women treated for breast cancer, associated health professionals (e.g., cancer nurse specialists, psychologists) and cancer support organisations (e.g., Breast Cancer Now) contributed to the adaptation of the original intervention and its acceptability testing.

Prior to the grant submission, a PPI group of women treated for BC from diverse backgrounds (regarding age, sexual orientation, ethnicity, geographic location, socio-economic status, stage of treatment/recovery) informed several aspects of research design, such as the participant eligibility criteria, the control arm, the self-report validated outcome measures, and data collection formats.

A study PPI group has been assembled, comprising women treated for BC of different ages, ethnicities, sexual orientations, geographical locations, and socio-economic backgrounds, plus representatives from cancer organisations, including those supporting underserved groups (e.g., Black Women of Colour for people of colour). The Six UK Standards for Public Involvement are being followed. For example, the group meet online to facilitate accessibility, they have already contributed to decision making, and they are being reimbursed for each meeting. Training and support will be delivered to the PPI lead and PPI group to develop their research knowledge skills (e.g., via online training, conference attendance; NIHR resources for public involvement).

The study PPI group will meet online up to three times a year and have already advised on research protocols and participant-facing materials (e.g., PIS, ICF). Throughout the project, their input will be involved in the following activities: advising on strategies to maximise recruitment, engagement and retention; advising on culturally and ethnically relevant issues as necessary; helping conduct interviews, help interpret research findings; advising on research reports; selecting specific outlets for dissemination; designing and producing summary reports for key parties, including research participants; and designing and delivering presentations to national cancer organisations or at academic conferences.

The SSC also includes two women with lived experience of BC cancer and two representatives from national cancer support organisations (see 10.2).

### Publication and dissemination policy

As described in 10.3.2, we will work with the PPI group to construct a dissemination plan that will be widely accessible to a broad range of users. As mentioned in 8.1 and 8.2, all participants will be assigned a unique participant ID, and interview participants will be assigned a pseudonym. All publications will ensure that no identifiable information will be included to ensure confidentiality.

1. **REFERENCES**

Association of Breast Surgery. (2020) Statement for the Association of Breast Surgery, 15th March 2020.

Begovic-Juhant, A., et al., (2012). Impact of body image on depression and quality of life among women with breast cancer. Journal of psychosocial oncology. 30(4): p. 446-460.

Brady, M.J., et al., (1997). Reliability and validity of the Functional Assessment of Cancer Therapy-Breast quality-of-life instrument. Journal of clinical oncology, 1997. 15(3): p. 974-986.

Braun, V. and Clarke, V. (2006). Using thematic analysis in psychology. Qualitative research in psychology, 2006. 3(2): p. 77-101.

Braun, V. and Clarke, V. (2022) Conceptual and design thinking for thematic analysis. Qualitative Psychology. 9(1): p. 3.

Breast Cancer Now. (2022). Over 1,500 breast cancer patients facing “many months, possibly years” without reconstruction, amid delays to surgery following pandemic.

Breast Cancer Now. (2022). 44% of women with breast cancer say it negatively impacted their body image.

British Association of Plastic, Reconstructive and Aesthetic Surgeons. (2020). Re-establishing breast reconstruction services.

Cancer Research UK. (2022). Breast cancer statistics. Available from: https://www.cancerresearchuk.org/health-professional/cancer-statistics/statistics-by-cancer-type/breast-cancer#heading-Three.

Carreira, H., et al., (2021). Associations between breast cancer survivorship and adverse mental health outcomes: A matched population-based cohort study in the United Kingdom. PLoS medicine. 18(1): p. e1003504.

Cash, T.F. and Pruzinsky, T. (2002). Body image: a handbook of theory, research, and clinical practice. New York: Guilford Publications.

Cohen, M., et al. (2012). Communication between breast cancer patients and their physicians about breast-related body image issues. Plastic surgical nursing: official journal of the American Society of Plastic and Reconstructive Surgical Nurses. 32(3): p. 101.

Cousson-Gelie, F., et al. (2007). Do anxiety, body image, social support and coping strategies predict survival in breast cancer? A ten-year follow-up study. Psychosomatics. 48(3): p. 211-216.

Davis, C., et al. (2020). Body image in older breast cancer survivors: A systematic review. Psycho‐Oncology. 29(5): p. 823-832.

Falk Dahl, C.A., et al. (2010). A study of body image in long‐term breast cancer survivors. Cancer, 2010. 116(15): p. 3549-3557.

Gov.uk (2022) Levelling Up the United Kingdom. [Levelling Up the United Kingdom - GOV.UK (www.gov.uk)](https://www.gov.uk/government/publications/levelling-up-the-united-kingdom)

Helms, R.L., et al. (2008). Body image issues in women with breast cancer. Psychology, Health and medicine. 13(3): p. 313-325.

Herdman, M., et al. (2011). Development and preliminary testing of the new five-level version of EQ-5D (EQ-5D-5L). Quality of life research. 20(10): p. 1727-1736.

Hopwood, P., et al. (2001). A body image scale for use with cancer patients. European Journal of Cancer. 37(2): p. 189-197.

Keetharuth, A.D., et al. (2018). Recovering Quality of Life (ReQoL): a new generic self-reported outcome measure for use with people experiencing mental health difficulties. The British Journal of Psychiatry. 212(1): p. 42-49.

Keetharuth A.D., et al. (2021). Estimating a Preference-Based Index for Mental Health From the Recovering Quality of Life Measure: Valuation of Recovering Quality of Life Utility Index. Value Health. 24(2):281-290. doi: 10.1016/j.jval.2020.10.012.

Kessler, R.C., et al. (2002). Short screening scales to monitor population prevalences and trends in non-specific psychological distress. Psychological medicine. 32(6): p. 959-976.

Lam, W.W., et al (2012). Trajectories of body image and sexuality during the first year following diagnosis of breast cancer and their relationship to 6 years psychosocial outcomes. Breast Cancer Research and Treatment. 131(3): p. 957-967.

Lewis-Smith, H. (2017). Body image in midlife: Developing a psychosocial intervention for women who have received treatment for breast cancer. University of the West of England.

Lewis-Smith, H., et al. (2018). A pilot study of a body image intervention for breast cancer survivors. Body image. 27: p. 21-31.

Lewis‐Smith, H., et al. (2016). A systematic review of interventions on body image and disordered eating outcomes among women in midlife. International Journal of Eating Disorders. 49(1): p. 5-18.

Lewis‐Smith, H., et al. (2018). Efficacy of psychosocial and physical activity‐based interventions to improve body image among women treated for breast cancer: A systematic review. Psycho‐Oncology. 27(12): p. 2687-2699.

Lewis-Smith, H., et al. (2020). Psychological and sociocultural influences on body image among midlife women with and without a history of breast cancer: Testing the Tripartite Influence Model of Body Image. Body Image. 35: p. 114-125.

Mausbach, B.T., et al. (2020). Healthcare use and costs in adult cancer patients with anxiety and depression. Depression and anxiety. 37(9): p. 908-915.

McWilliam, C.L., et al. (2000). Breast cancer patients’ experiences of patient–doctor communication: a working relationship. Patient education and counselling. 39(2): p. 191-204.

Moreira, H. and Canavarro, M.C. (2010). A longitudinal study about the body image and psychosocial adjustment of breast cancer patients during the course of the disease. European Journal of Oncology Nursing. 14(4): p. 263-270.

Mundt, J.C., et al. (2002). The Work and Social Adjustment Scale: a simple measure of impairment in functioning. The British Journal of Psychiatry. 180(5): p. 461-464.

O’Cathain, A., et al. (2015). Maximising the impact of qualitative research in feasibility studies for randomised controlled trials: guidance for researchers. Pilot and Feasibility Studies. 1(1): p. 1.

Paterson, C., et al. (2016). Body image in younger breast cancer survivors: a systematic review. Cancer nursing. 39(1): p. E39.

Pusic, A.L., et al. (2009). Development of a new patient-reported outcome measure for breast surgery: the BREAST-Q. Plastic and reconstructive surgery. 124(2): p. 345-353.

Richards, D.A., et al. (2016). Cost and Outcome of Behavioural Activation versus Cognitive Behavioural Therapy for Depression (COBRA): a randomised, controlled, non-inferiority trial. The Lancet. 388(10047): p. 871-880.

Rosman, S. (2004). Cancer and stigma: experience of patients with chemotherapy-induced alopecia. Patient education and counselling. 52(3): p. 333-339.

Skivington, K., et al. (2021). A new framework for developing and evaluating complex interventions: update of Medical Research Council guidance. bmj. 374.

Strauss, C., et al. (2020). Low-Intensity Guided Help Through Mindfulness (LIGHTMIND): study protocol for a randomised controlled trial comparing supported mindfulness-based cognitive therapy self-help to supported cognitive behavioural therapy self-help for adults experiencing depression. Trials. 21(1): p. 1-10.

Tylka, T.L. and Wood-Barcalow, N.L. (2015). The Body Appreciation Scale-2: item refinement and psychometric evaluation. Body image. 12: p. 53-67.

Van Beek, F.E., et al. (2021). Psychological problems among cancer patients in relation to healthcare and societal costs: A systematic review. Psycho‐Oncology. 30(11): p. 1801-1835.

### APPENDICES

The below table includes a list of study documents and patient-facing materials and documents their versions and associated dates.

| **Appendix No.** | **Document Title** | **Version No.** | **Date** |
| --- | --- | --- | --- |
| 1 | ABC Patient Invitation General + Expression of Interest Form | 1 | 18/04/2024 |
| 2 | ABC Main Study Participant Information Sheet | 2 | 23/05/2024 |
| 3 | ABC Poster | 1 | 18/04/2024 |
| 4 | ABC Patient Invitation Leeds + Expression of Interest Form | 1 | 18/04/2024 |
| 5 | ABC Social Media Post | 1 | 18/04/2024 |
| 6 | ABC Main Study Consent Form | 1 | 18/04/2024 |
| 7 | ABC Interview Participant Information Sheet | 2 | 23/05/2024 |
| 8 | ABC Interview Consent Form | 1 | 18/04/2024 |
| 9 | ABC Programme Feedback (Rating Scales + Open-ended Questions) | 1 | 18/04/2024 |
| 10 | ABC Research Process Feedback (Rating Scales + Open-ended Questions) | 1 | 18/04/2024 |
| 11 | Interview Schedule: Did Not Consent | 1 | 18/04/2024 |
| 12 | Interview Schedule: Dropped Out Post-Randomisation | 1 | 18/04/2024 |
| 13 | Interview Schedule: Control Participants Dropped Out During Study | 1 | 18/04/2024 |
| 14 | Interview Schedule: Intervention Participants Dropped Out During Study | 1 | 18/04/2024 |
| 15 | Interview Schedule: Control Participants Finished Study | 1 | 18/04/2024 |
| 16 | Interview Schedule: Intervention Participants Finished Study | 1 | 18/04/2024 |
| 17 | Demographic and Breast Cancer Information | 1 | 18/04/2024 |
| 18 | Kessler Psychological Distress Scale | n/a | |
| 19 | Body Appreciation Scale-2 | n/a | |
| 20 | Functional Assessment of Cancer Therapy – Breast: Breast Cancer Subscale | n/a | |
| 21 | Hopwood Body Image Scale | n/a | |
| 22 | BREAST-Q: Sexual Well-Being Scale | n/a | |
| 23 | Modified version of the Adult Service Use Schedule: T1 | 1 | 18/04/2024 |
| 24 | Modified version of the Adult Service Use Schedule: T3 | 1 | 18/04/2024 |
| 25 | Modified version of the Adult Service Use Schedule: T4 | 1 | 18/04/2024 |
| 26 | EQ-5D-5L | n/a | |
| 27 | Recovering Quality of Life-Utility Index | n/a | |
| 28 | Work and Social Adjustment Scale | n/a | |
| 29 | Data Management Plan | 1 | 16/04/2024 |
| 30 | Protocol Amendment History | n/a | |
| 31 | Follow-up ABC Acceptability questions | n/a | |
| 32 | Additional Demographics Question | n/a | |

**12.1 Appendix 1: ABC Patient Invitation General + Expression of Interest Form**


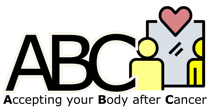


ADD NHS TRUST LOGO HERE

ADD NHS TRUST PI’s DETAILS HERE

**Dr Helena Lewis-Smith**

Associate Professor of Psychology

Centre for Appearance Research

College of Health, Science, and Society

University of the West of England

Date: xxxxxx

Dear [PATIENT NAME],

**Accepting your Body after Cancer (ABC): A feasibility study**

The [NHS TRUST) is participating in a national research study, which is aiming to improve how women feel about their body following treatment for breast cancer. We are writing to you to invite you to take part in this research study. Please read the following information and the enclosed Participant Information Sheet, to decide if you may be interested in taking part.

Following treatment for breast cancer, many women can experience difficulty with how their body looks and feels different. This can lead to low mood, anxiety, and negatively affect many other aspects of daily life. There is currently very little support available to help women feel more comfortable with their body following breast cancer treatment.

Researchers at the University of the West of England, Kings College London, and the University of Bristol, are working together to find out whether a group-based programme that is delivered online might be helpful for women. The first stage is to conduct a small ‘feasibility’ study. This will help us understand whether we should do a larger study and how best it should be done to meet women’s needs. It is hoped that this research will eventually lead to the availability of a body image programme that could benefit thousands of women treated for breast cancer.

We are contacting you because you are someone who has expressed a degree of difficulty with how your body or appearance has changed because of breast cancer. You may therefore be interested in taking part in this research study.

**We have enclosed a Participant Information Sheet that explains a bit more about the study and what taking part would involve. If you are interested in potentially taking part, please return the enclosed reply slip in the enclosed pre-paid envelope.**

If we have not heard from you after 2 weeks, we may telephone you. However, you will not be contacted again after this.

If you have any questions about the study, please call 0117 3281895 or e-mail [Helena.lewis-smith@uwe.ac.uk](mailto:Helena.lewis-smith@uwe.ac.uk).

Thank you for taking the time to consider this invitation.

Yours sincerely,

[NHS TRUST PI’S NAME]

**REPLY SLIP**

Please complete the reply slip and return in the enclosed pre-paid envelope to the following address:

Dr Helena Lewis-Smith,

School of Social Sciences,
College of Health, Science, and Society,
University of the West of England,
Frenchay Campus,

Bristol, BS16 1QY

Alternatively, you can indicate your interest via email or telephone, as follows:

Email: [Helena.lewis-smith@uwe.ac.uk](mailto:Helena.lewis-smith@uwe.ac.uk)

Telephone: 0117 3281895

Name: ………………………………………………………………………………………...……………………………........

**Would you like to find out more about the study?** (please tick as appropriate):

**□** YES  **□** NO

**If you selected ‘NO’, would you mind briefly sharing why this is, for our own records?** (please provide a brief explanation)

…………………………………………………………………………………………………………………………………………

…………………………………………………………………………………………………………………………………………

**Which mode(s) of contact would you prefer?** (please tick and fill in the relevant details):

**□** Post - Postal Address: ……………………………………………………………………………………………………

…………………………………………………………………………………………………………………………………………

…………………………………………………………………………………………………………………………………………

**□** Email – Email address: …………………………………………………………………………….......................

**□** Telephone – Telephone number: ………………………………………………....................................

**12.2 Appendix 2: ABC Main Study Participant Information Sheet**

**
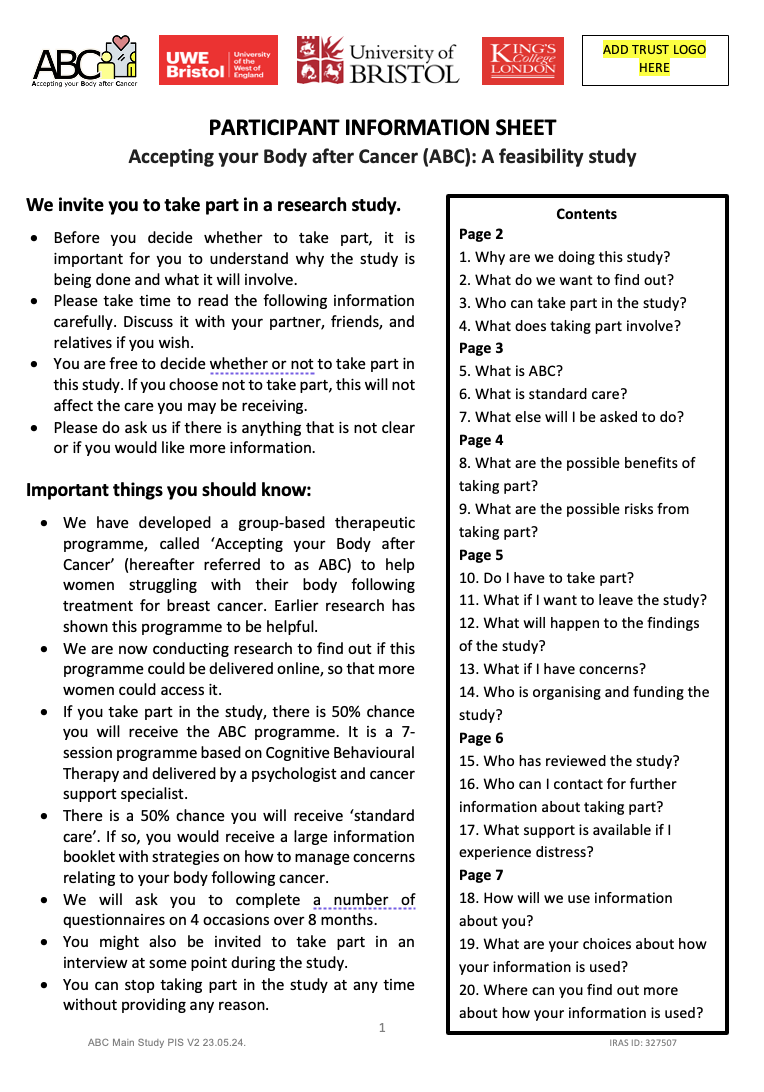
**

**
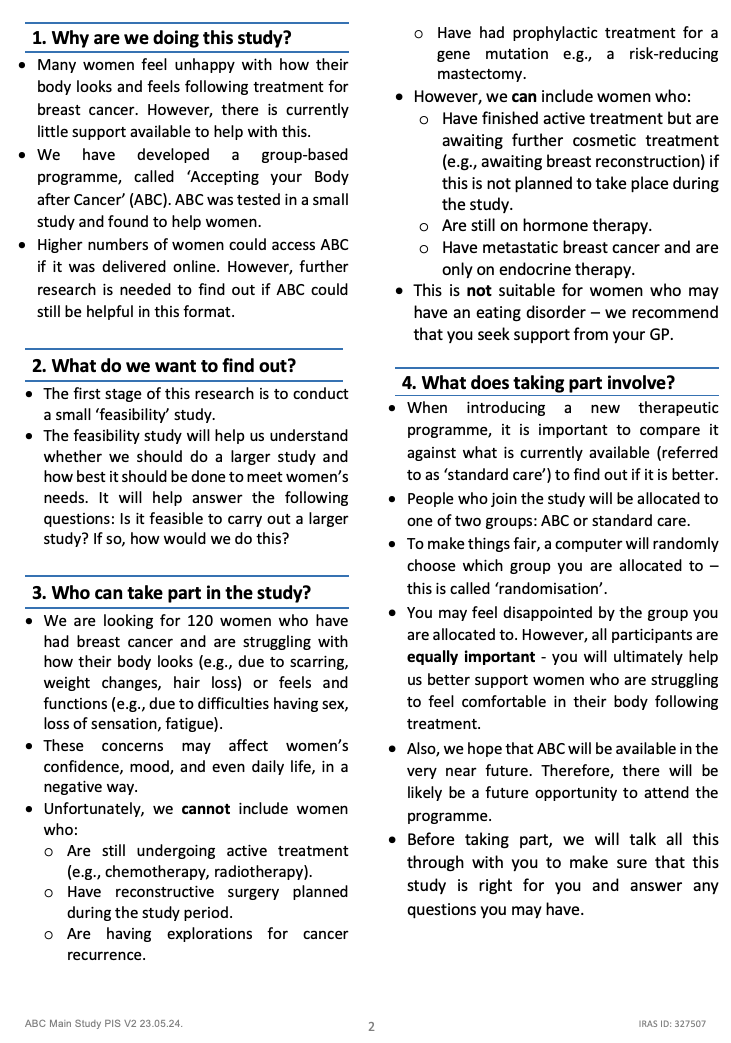
**

**
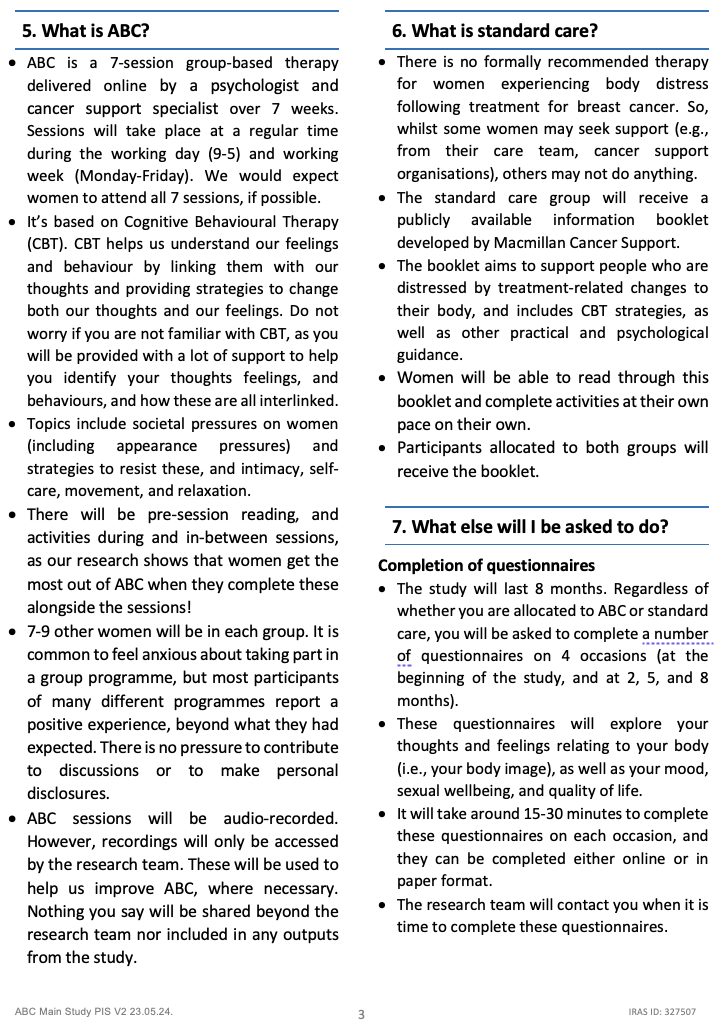
**

**
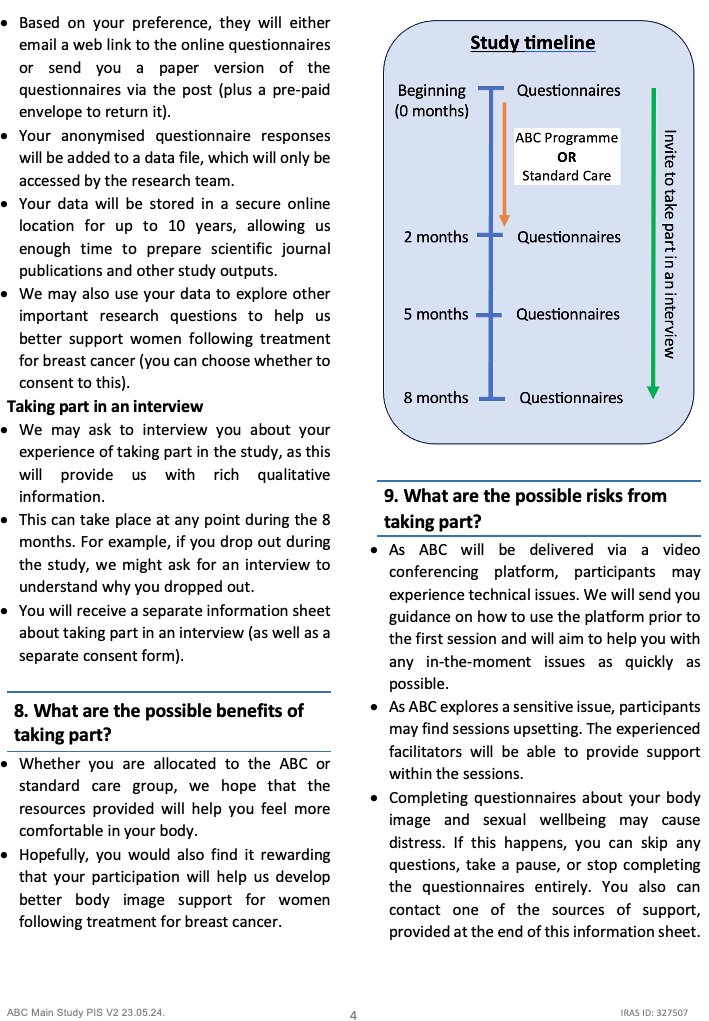
**

**
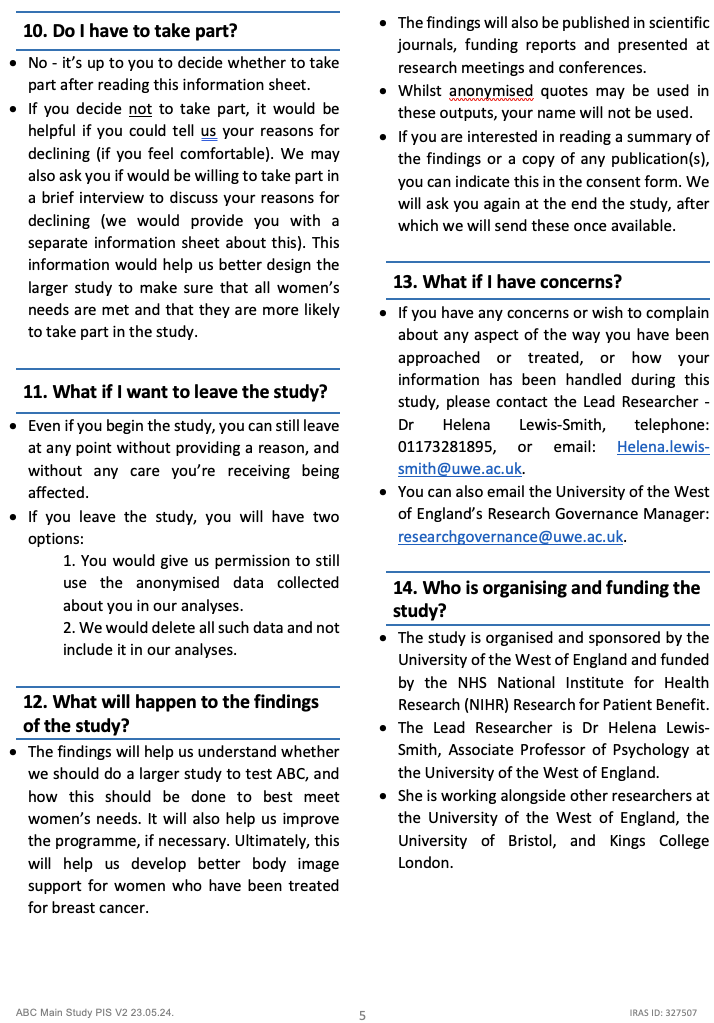
**


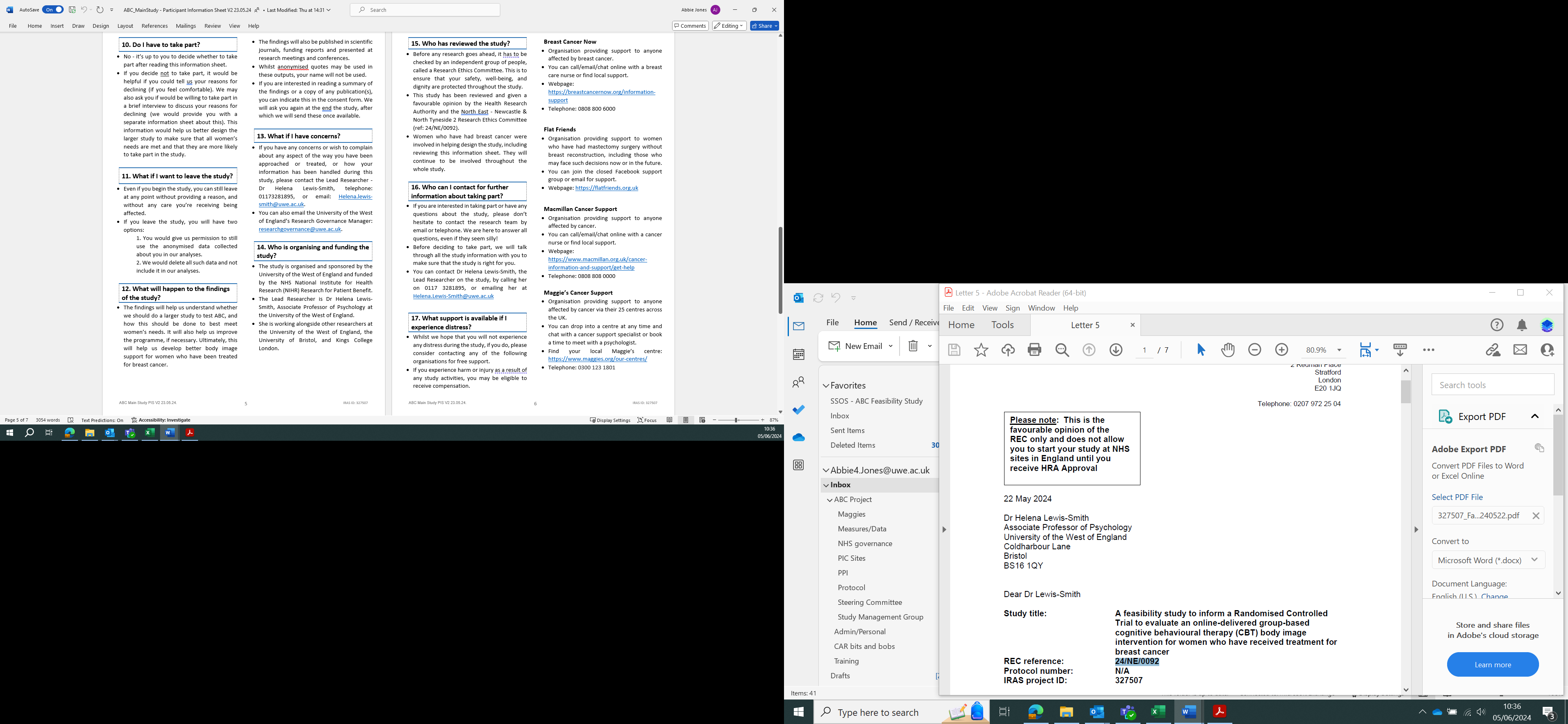


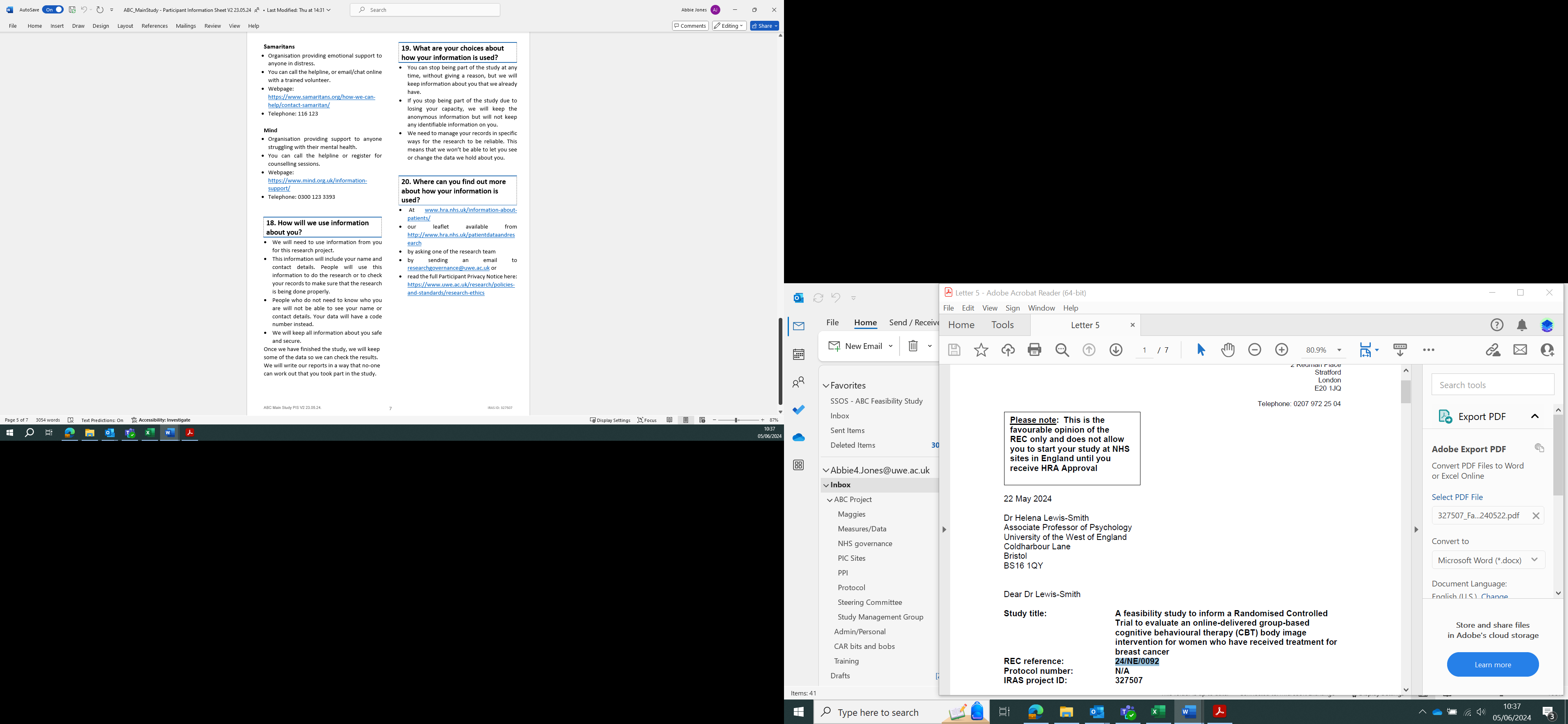


**12.3 Appendix 3: ABC Poster**

**12.4 Appendix 4: ABC Patient Invitation Leeds + Expression of Interest Form**


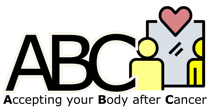


**Dr Sue Hartup**

Nurse Research Fellow, Breast Oncology

St James’s University Hospital, Leeds

**Dr Helena Lewis-Smith**

Associate Professor of Psychology

Centre for Appearance Research

College of Health, Science, and Society

University of the West of England

Date: xxxxxx

Dear [PATIENT NAME],

**Accepting your Body after Cancer (ABC): A feasibility study**

The Leeds Teaching Hospitals NHS Trust is participating in a national research study, which is aiming to improve how women feel about their body following treatment for breast cancer. We are writing to you to invite you to take part in this research study. Please read the following information and the enclosed Participant Information Sheet, to decide if you may be interested in taking part.

Following treatment for breast cancer, many women can experience difficulty with how their body looks and feels different. This can lead to low mood, anxiety, and negatively affect many other aspects of daily life. There is currently very little support available to help women feel more comfortable with their body following breast cancer treatment.

Researchers at the University of the West of England, Kings College London, and the University of Bristol, are working together to find out whether a group-based programme that is delivered online might be helpful for women. The first stage is to conduct a small ‘feasibility’ study. This will help us understand whether we should do a larger study and how best it should be done to meet women’s needs. It is hoped that this research will eventually lead to the availability of a body image programme that could benefit thousands of women treated for breast cancer.

We are contacting you because you are someone who has been diagnosed and treated for breast cancer. You may therefore be eligible to take part in this research study.

**We have enclosed a Participant Information Sheet that explains a bit more about the study and what taking part would involve. If you are interested in potentially taking part, please return the enclosed reply slip in the enclosed pre-paid envelope.**

If we have not heard from you after 2 weeks, we may telephone you. However, you will not be contacted again after this.

If you have any questions about the study, please call 0117 3281895 or e-mail [Helena.lewis-smith@uwe.ac.uk](mailto:Helena.lewis-smith@uwe.ac.uk).

Thank you for taking the time to consider this invitation.

Yours sincerely,

**Sue Hartup**

**Oncology Research Team**

**REPLY SLIP**

Please complete the reply slip and return in the enclosed pre-paid envelope to the following address:

Dr Helena Lewis-Smith,

School of Social Sciences,
College of Health, Science, and Society,
University of the West of England,
Frenchay Campus,

Bristol, BS16 1QY

Alternatively, you can indicate your interest via email or telephone, as follows:

Email: [Helena.lewis-smith@uwe.ac.uk](mailto:Helena.lewis-smith@uwe.ac.uk)

Telephone: 0117 3281895

Name: …………………………………………………………………………………………...……………………………...............

**Would you like to find out more about the study?** (please tick as appropriate):

**□** YES  **□** NO

**If you selected ‘NO’, would you mind briefly sharing why this is, for our own records?** (please provide a brief explanation)

………………………………………………………………………………………………………………………………………………

………………………………………………………………………………………………………………………………………………

………………………………………………………………………………………………………………………………………………

**Which mode(s) of contact would you prefer?** (please tick and fill in the relevant details):

**□** Post - Postal Address: ………………………………………………………………………………………………………..

………………………………………………………………………………………………………………………………………………

………………………………………………………………………………………………………………………………………………

**□** Email – Email address: …………………………………………………………………………….......................…...

**□** Telephone – Telephone number: ………………………………………………....................................……

**12.5 Appendix 5: ABC Social Media Post**

**
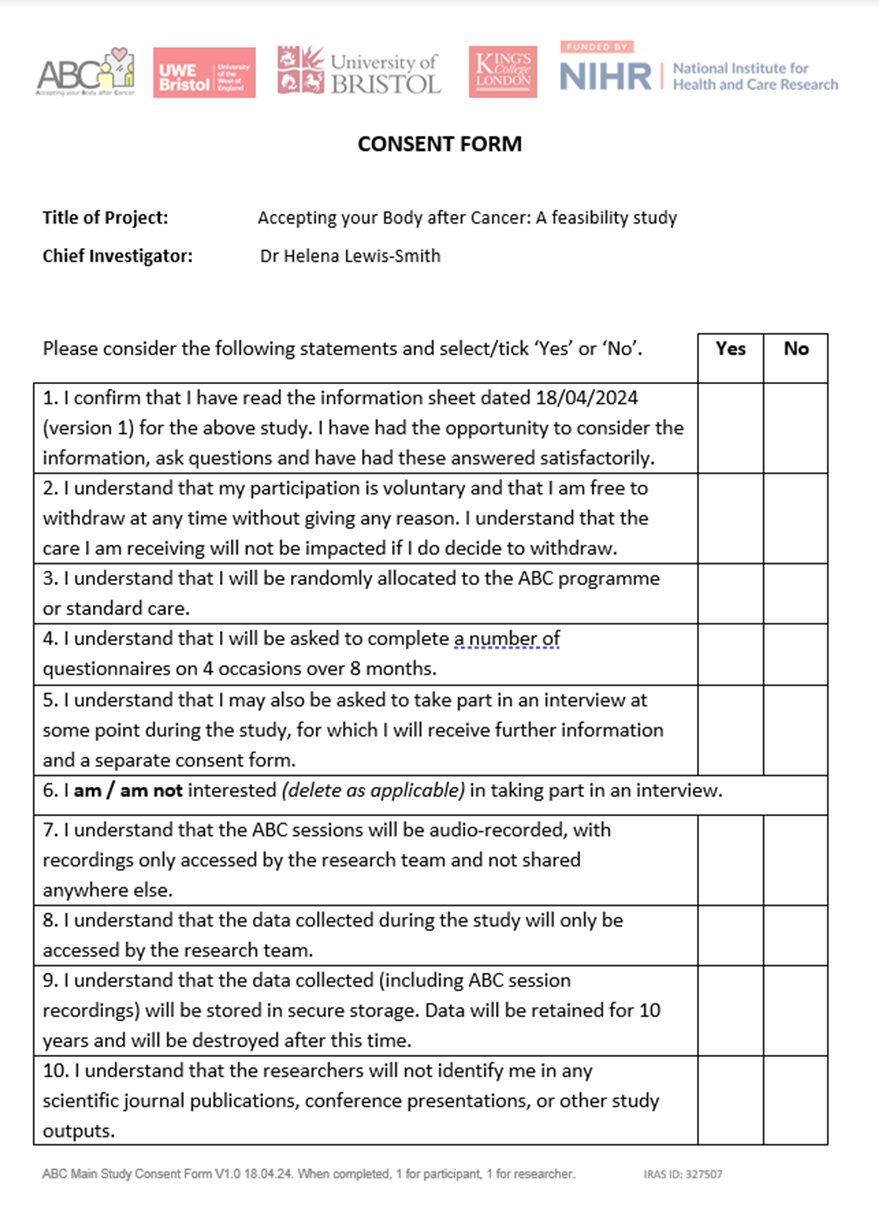
12.6 Appendix 6: ABC Main Study Consent Form**

**
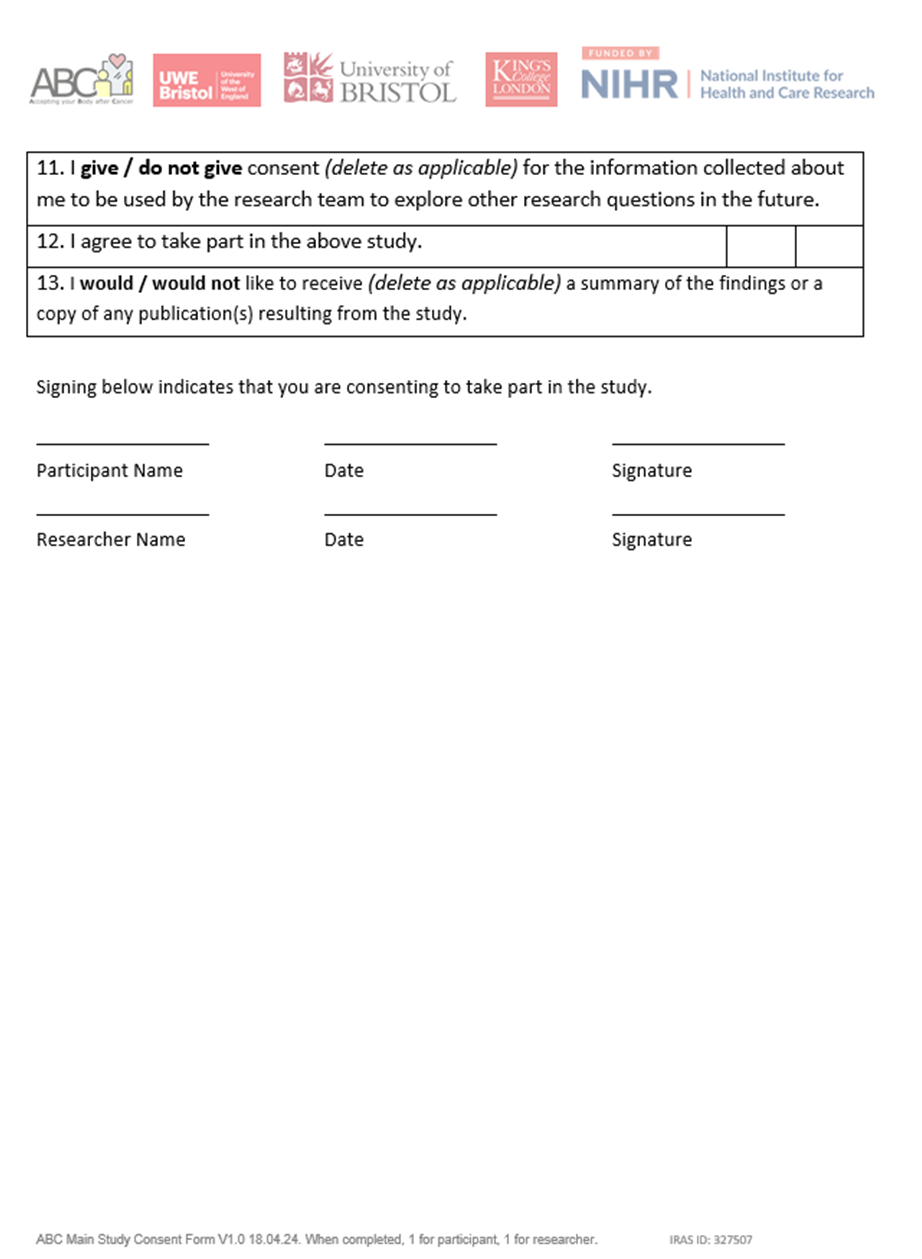
**

**12.7 Appendix 7: ABC Interview Participant Information Sheet**


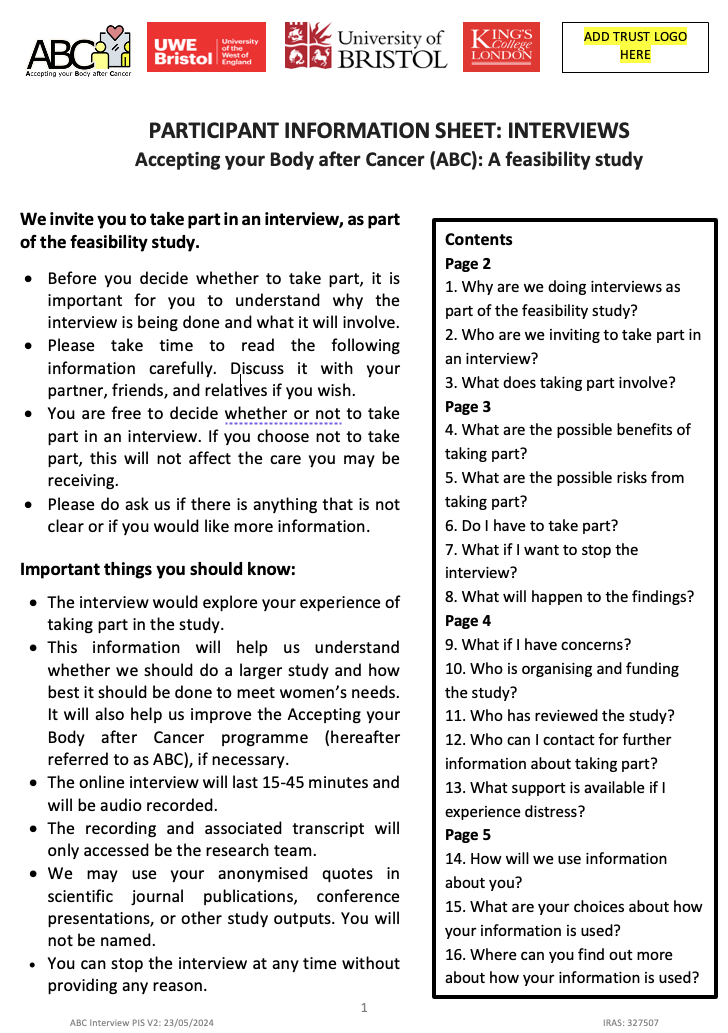


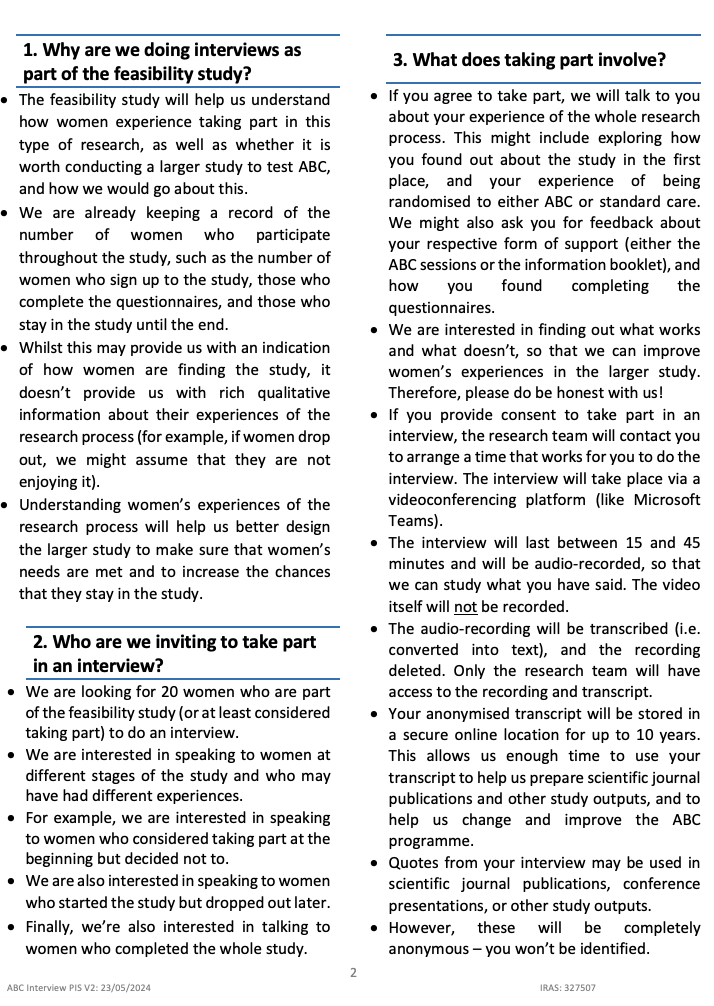


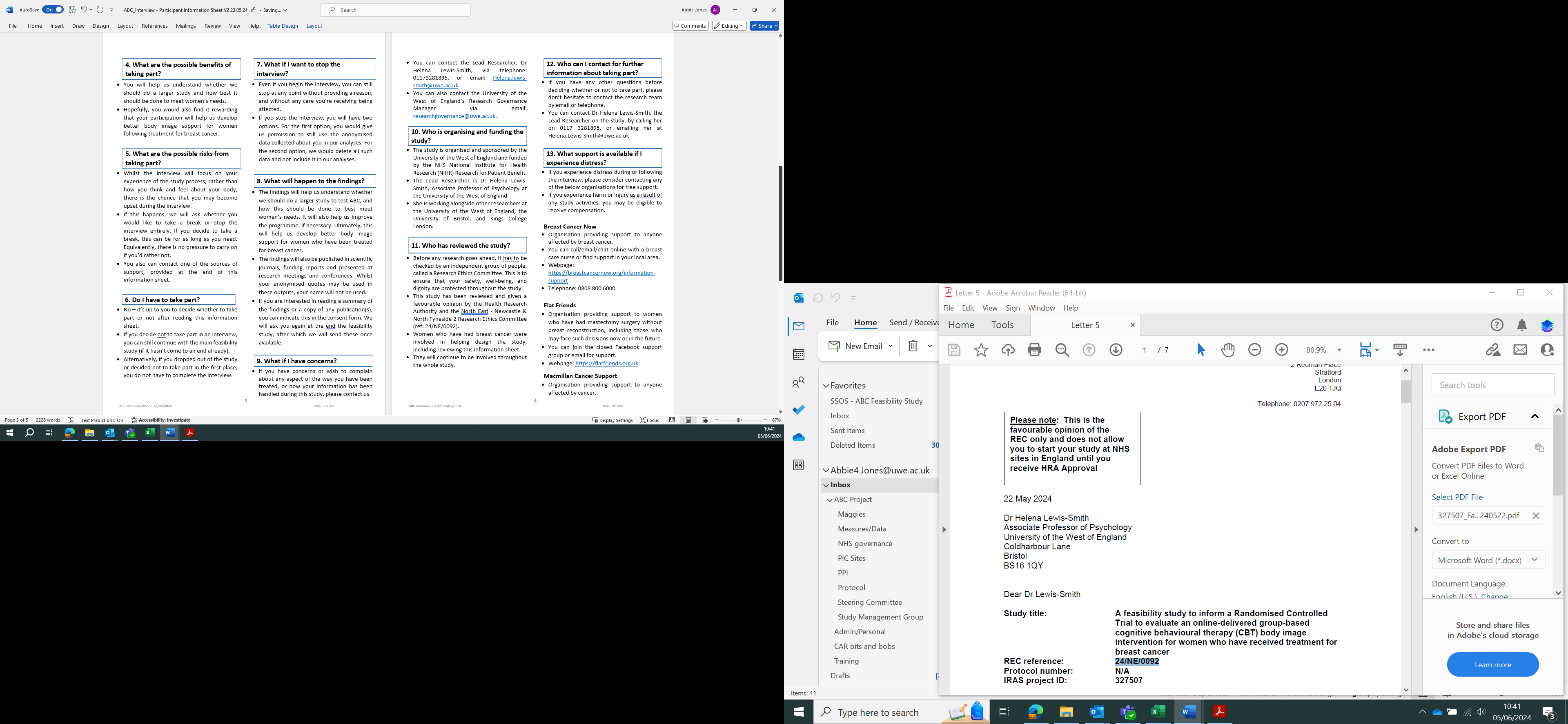


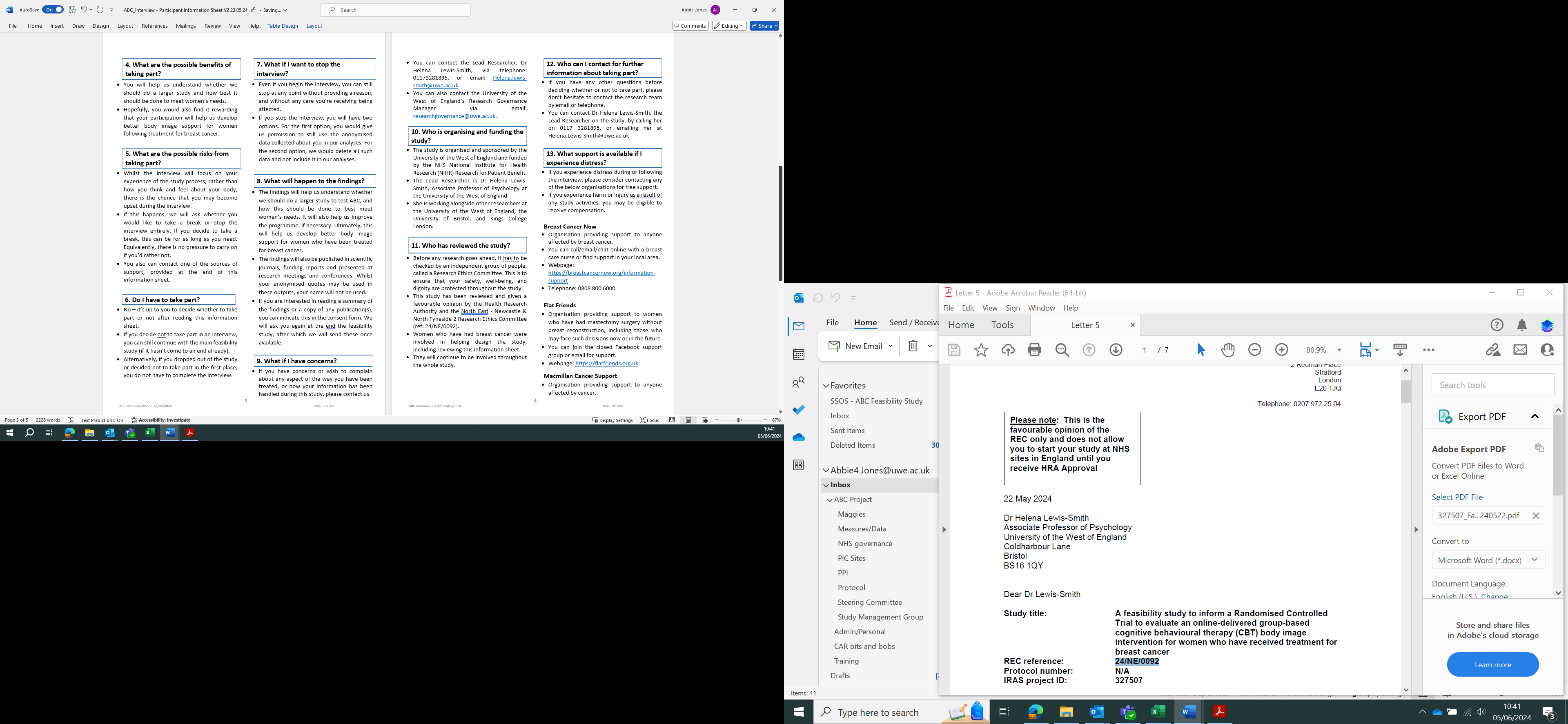


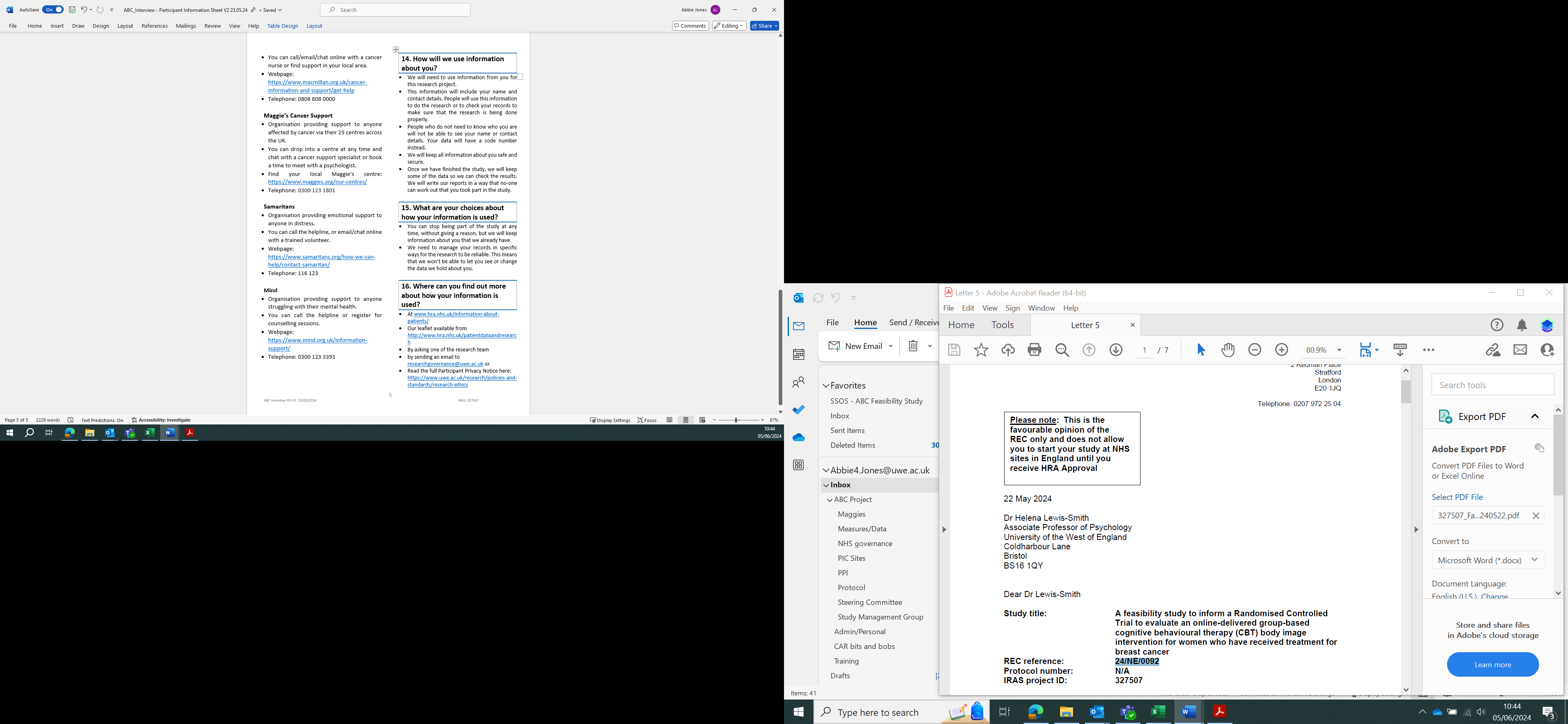


**12.8** **Appendix 8: ABC Interview Consent Form**

**INTERVIEW CONSENT FORM**

**Title of Project:** Accepting your Body after Cancer: A feasibility study

**Chief Investigator:** Dr Helena Lewis-Smith

| Please consider the following statements and select/tick ‘Yes’ or ‘No’. | **Yes** | **No** |
| --- | --- | --- |
| 1. I confirm that I have read the information sheet dated 18/04/2024 (version 1) for the above study. I have had the opportunity to consider the information, ask questions and have had these answered satisfactorily. |  |  |
| 2. I understand that my participation is voluntary and that I am free to withdraw at any time without giving any reason. I understand that the care I am receiving will not be impacted if I do decide to withdraw. |  |  |
| 3. I agree to the interview being audio-recorded. |  |  |
| 4. I understand that the audio-recorded interview and associated transcript will only be accessed by the research team. |  |  |
| 5. I understand that the audio-recorded interview and transcript will be stored in secure storage. The interview transcript will be retained for 10 years and will be destroyed after this time, whilst the audio-recorded interview will be deleted once it has been transcribed. |  |  |
| 6. I agree to anonymised quotes from my interview being used in scientific journal publications, conference presentations, or other study outputs. I understand that I will not be named in any outputs. |  |  |
| 7. I agree to take part in the interview. |  |  |
| 8. I **would / would not** like to receive *(delete as applicable)* a summary of the findings or a copy of any publication(s) resulting from the study. | | |

Signing below indicates that you are consenting to take part in the interview.

Participant Name Date Signature

Researcher Name Date Signature

**12.9 Appendix 9: ABC Programme Feedback (Rating Scales + Open-ended Questions)**

**ABC Feedback**

Now that you have finished attending ‘Accepting your Body after Cancer’, we would appreciate your feedback on the programme.

This feedback is very valuable, as it will help us improve the programme if necessary. Therefore, please be as honest as possible. Don't forget - your responses are anonymous!

First of all, how **helpful** did you find the programme overall?

|  | Not at all helpful  1 | 2 | 3 | 4 | Very helpful  5 |
| --- | --- | --- | --- | --- | --- |
| Please select one option: |  |  |  |  |  |

Would you like to tell us more about this?

How **helpful** did you find the CBT approach and strategies in the programme?

|  | Not at all helpful  1 | 2 | 3 | 4 | Very helpful  5 |
| --- | --- | --- | --- | --- | --- |
| Please select one option: |  |  |  |  |  |

Would you like to tell us more about this?

What **topics and/or exercises** covered in the programme did you find the **most** helpful?

What **topics and/or exercises** covered in the programme did you find the **least** helpful (if any)?

Were there any **topics** that you felt were **missed** in the programme?

You are now going to be asked some questions about the format and content of ABC.

What did you think about the **length** **of the overall programme** (7 sessions)?

|  | Too short  1 | The right length  2 | Too long  3 |
| --- | --- | --- | --- |
| Please select one option: |  |  |  |

What did you think about the **length** **of each** **session**?

|  | Too short  1 | The right length  2 | Too long  3 |
| --- | --- | --- | --- |
| Please select one option: |  |  |  |

What did you think about the **frequency** **of each** **session (weekly)**?

|  | Not often enough  1 | The right frequency  2 | Too often  3 |
| --- | --- | --- | --- |
| Please select one option: |  |  |  |

How many of the **7 sessions** were you able to attend?

|  | 1 | 2 | 3 | 4 | 5 | 6 | 7 |
| --- | --- | --- | --- | --- | --- | --- | --- |
| Please select one option: |  |  |  |  |  |  |  |

How did you feel about the timing of the sessions (i.e., taking place on a weekday and between 9 and 5)?

How did you find the **amount** **of** **content** in each session?

|  | Too little content  1 | The right amount of content  2 | Too much content  3 |
| --- | --- | --- | --- |
| Please select one option: |  |  |  |

How easy did you find the **content to understand?**

|  | Not easy at all  1 | Sometimes easy  2 | Very easy  3 |
| --- | --- | --- | --- |
| Please select one option: |  |  |  |

How did you find the **number** **of** **between-session activities** you were given?

|  | Too few activities  1 | The right number of activities  2 | Too many activities  3 |
| --- | --- | --- | --- |
| Please select one option: |  |  |  |

Did you find the **between-session activities helpful**?

|  | Not helpful at all  1 | Sometimes helpful  2 | Very helpful  3 |
| --- | --- | --- | --- |
| Please select one option: |  |  |  |

How many women were in your group overall? (not including the two facilitators)?

What did you think about this **group size (i.e., the number of women in the group)?**

|  | Too small  1 | The right size  2 | Too big  3 |
| --- | --- | --- | --- |
| Please select one option: |  |  |  |

How helpful was it attending the sessions with **others who have had breast cancer**?

|  | Not helpful at all  1 | Sometimes helpful  2 | Very helpful  3 |
| --- | --- | --- | --- |
| Please select one option: |  |  |  |

How helpful were the **group facilitators (i.e., the duo who ran the sessions?)**

|  | Not helpful at all  1 | Sometimes helpful  2 | Very helpful  3 |
| --- | --- | --- | --- |
| Please select one option: |  |  |  |

Did you find the programme being **run online an issue?** (e.g., technical issues, challenges communication with others?)

|  | Wasn’t an issue at all  1 | Was sometimes an issue  2 | Was a large issue throughout  3 |
| --- | --- | --- | --- |
| Please select one option: |  |  |  |

If one of the people **delivering the programme was a man**, how comfortable did you feel throughout the sessions?

|  | Not at all comfortable  1 | 2 | 3 | 4 | Very comfortable  5 |
| --- | --- | --- | --- | --- | --- |
| Please select one option: |  |  |  |  |  |

If two women delivered the programme, would having had a **man (as a psychologist or cancer support specialist) stopped you** from attending the programme?

|  | Would have stopped me  1 | 2 | 3 | 4 | Would not have stopped me  5 |
| --- | --- | --- | --- | --- | --- |
| Please select one option: |  |  |  |  |  |

Would you like to expand on or explain any of the above answers?

Is there any other feedback you have on the programme, or anything we could do better?

**12.10 Appendix 10: ABC Research Process Feedback (Rating Scales + Open-ended Questions)**

**Research Process Feedback**

As you come to the end of the research study, we would appreciate your feedback on the whole research process.

This feedback is very valuable, as it will help us plan the larger study to best need women’s needs. Therefore, please be as honest as possible. Don't forget - your responses are anonymous!

Thinking back to the very beginning, was the **information provided about the** **study** clear? (i.e., were you able to understand what taking part would involve?)

|  | Not at all clear  1 | 2 | 3 | 4 | Very clear  5 |
| --- | --- | --- | --- | --- | --- |
| Please select one option: |  |  |  |  |  |

Was the **contact and** **communication** throughout the study appropriate? (e.g., were instructions clear? Did you feel comfortable?)

|  | Not at all appropriate  1 | 2 | 3 | 4 | Very appropriate  5 |
| --- | --- | --- | --- | --- | --- |
| Please select one option: |  |  |  |  |  |

Did you find the **questions in the** **questionnaires** **relevant?** (e.g., did the thoughts, feelings, and behaviors asked about reflect how body image concerns may have impacted you?)

|  | Not relevant at all  1 | Sometimes relevant  2 | Very relevant  3 |
| --- | --- | --- | --- |
| Please select one option: |  |  |  |

Did you find completing the **questionnaires tiring?**

|  | Not at all tiring  1 | 2 | 3 | 4 | Very tiring  5 |
| --- | --- | --- | --- | --- | --- |
| Please select one option: |  |  |  |  |  |

Would you like to explain or expand on any of the above answers?

___________________________________________________________________________________________________________________________________________________________________________________________________________________________________________________

Were you **already** **familiar** with the Macmillan ‘Body image and cancer’ booklet you were given before this study?

|  | Yes | No |
| --- | --- | --- |
| Please select one option: |  |  |

**How much** of this booklet did you read during this study?

|  | None of it | Less than a quarter of it | Up to half of it | Up to three quarters of it | The whole booklet |
| --- | --- | --- | --- | --- | --- |
| Please select one option: |  |  |  |  |  |

If you read **any** of the booklet, how **helpful** did you find it?

|  | Not at all helpful  1 | 2 | 3 | 4 | Very helpful  5 |
| --- | --- | --- | --- | --- | --- |
| Please select one option: |  |  |  |  |  |

If you did **not read** any of the booklet, could you please tell us why?

________________________________________________________________________________________________________________________________________________

Did you seek any **other support** for your body image during the study?

|  | Yes | No |
| --- | --- | --- |
| Please select one option: |  |  |

If you **did** seek other support, could you please tell us what this was?

________________________________________________________________________________________________________________________________________________

How helpful did you find this support?

|  | Not at all helpful  1 | 2 | 3 | 4 | Very helpful  5 |
| --- | --- | --- | --- | --- | --- |
| Please select one option: |  |  |  |  |  |

If you had been randomly allocated to the **other condition** (either the ABC programme or control group), do you think you would have continued with the study?

|  | Yes | Maybe | No |
| --- | --- | --- | --- |
| Please select one option: |  |  |  |

Could you please tell us why you selected this?

________________________________________________________________________________________________________________________________________________

How have you found the overall experience of **taking part in this study?**

|  | A very negative experience  1 | 2 | 3 | 4 | A very positive experience  5 |
| --- | --- | --- | --- | --- | --- |
| Please select one option: |  |  |  |  |  |

Do you have any other feedback on the whole study, or suggestions for improvement?

____________________________________________________________________________________________________________________________________________________________________________________________________________________________________________________________________________________________________________________________________

_________________________________________________________________________________

_________________________________________________________________________________

**12.11 Appendix 11: Interview Schedule: Did Not Consent**

For individuals who read the participant information sheet but decided not to take part in the study.

- First of all, could you please tell me how you found out about the study?
  - Which NHS trust/charity/social media platform was that?
- How did you find the participant information sheet?
  - Was it clear/unclear?
  - Were you able to understand what taking part would involve?
- How did you find the contact and communication from the research team?
  - What information did they provide you with?
  - Did you feel comfortable talking with them?
  - Did they ask you whether you had any questions?
- Would you feel comfortable telling me why you decided not to take part in the study?
  - *Explore whether due to randomisation, group or online format, time commitment, having to complete questionnaires, not enough information, didn’t feel like they needed body image support, inadequate contact and communication from the research team, lack of remuneration, etc.*
- What could we have done differently to make you more likely to consider taking part?
  - *Explore whether suggested changes relate to randomisation, group or online format, time commitment, questionnaires, information provided, contact and communication from the research team, remuneration, etc.*
- Do you have anything else to add, or any questions?

**12.12 Appendix 12: Interview Schedule: Dropped Out Post-Randomisation**

For participants who dropped out of the study immediately following randomisation.

- First of all, could you please tell me how you found out about the study?
  - Which NHS trust/charity/social media platform was that?
- How did you find the participant information sheet?
  - Was it clear/unclear?
  - Were you able to understand what taking part would involve?
  - Were you able to understand that you would be randomised to one of two groups?
- How did you find the contact and communication from the research team?
  - What information did they provide you with?
  - Did you feel comfortable talking with them?
  - Did they ask you whether you had any questions?
- Would you feel comfortable telling me why you decided not to continue with the study?
  - *Explore whether due to disappointment with randomisation to specific group, the period having to wait to attend ABC, time commitment, having to complete questionnaires, not enough information, didn’t feel like they needed body image support, inadequate contact and communication from the research team, insufficient remuneration, etc.*
- What could we have done differently to make you more likely to continue with the study?
  - *Explore whether suggested changes relate to randomisation, group or online format, time commitment, questionnaires, information provided, inadequate contact and communication from the research team, remuneration, etc.*
- Do you have anything else to add, or any questions?

**12.13 Appendix 13: Interview Schedule: Control Participants Dropped Out During Study**

For participants who were randomised to the control group and dropped out before the last questionnaire.

- First of all, could you please tell me how you found out about the study?
  - Which NHS trust/charity/social media platform was that?
- How did you find the participant information sheet?
  - Was it clear/unclear?
  - Were you able to understand what taking part would involve?
  - Were you able to understand that you would be randomised to one of two groups?
- How did you find the contact and communication from the research team?
  - What information did they provide you with?
  - Did you feel comfortable talking with them?
  - Did they ask you whether you had any questions?
- Would you feel comfortable telling me why you decided not to continue until the end of the study?
  - *Explore whether due to disappointment with randomisation to control group, insufficient support from the Macmillan booklet, sought separate psychological support, having to complete multiple questionnaires, questionnaire irrelevant/upsetting, inadequate contact and communication from the research team, insufficient remuneration, etc.*
- What could we have done differently to make you more likely to continue with the study?
  - *Explore whether suggested changes relate to randomisation, materials/content provided to the control group, alternative of waitlist control, time commitment, questionnaires, information provided, contact and communication from the research team, remuneration, etc.*
- What else could we do to help encourage women to take part in a future study and to stay until the end?
- Do you have anything else to add, or any questions?

**12.14 Appendix 14: Interview Schedule: Intervention Participants Dropped Out During Study**

For participants who were randomised to the intervention group and dropped out before the last questionnaire or stopped attending the sessions.

- First of all, could you please tell me how you found out about the study?
  - Which NHS trust/charity/social media platform was that?
- How did you find the participant information sheet?
  - Was it clear/unclear?
  - Were you able to understand what taking part would involve?
  - Were you able to understand that you would be randomised to one of two groups?
- How did you find the contact and communication from the research team?
  - What information did they provide you with?
  - Did you feel comfortable talking with them?
  - Did they ask you whether you had any questions?
- Would you feel comfortable telling me why you decided not to continue until the end of the study?
  - *Explore whether due to insufficient support from ABC, time commitment of ABC, group setting or dynamics of ABC, technical issues accessing ABC, sought separate psychological support, having to complete multiple questionnaires, questionnaire irrelevant/upsetting, inadequate contact and communication from the research team, insufficient remuneration, etc.*
- What could we have done differently to make you more likely to continue with the study?
  - *Explore whether suggested changes relate to ABC (e.g., materials, format, number of sessions, between-session activities), questionnaires, information provided, contact and communication from the research team, remuneration, etc.*
- What else could we do to help encourage women to take part in a future study and to stay until the end?
- Do you have anything else to add, or any questions?

**12.15 Appendix 15: Interview Schedule: Control Participants Finished Study**

For participants who were randomised to the control group and completed the whole study (i.e., completed the last questionnaire).

- First of all, how have you found taking part in this study?
- Did you feel that the information provided about the study was clear?
  - Were you able to understand what taking part would involve?
- How did you find the contact and communication from the research team throughout the study?
  - Was each stage of the research (e.g., each questionnaire) clear?
  - Did you feel comfortable corresponding with them?
- How did you feel about the 50% chance that you would be able to attend the body image programme (i.e., being randomised)?
  - How did you feel when you were told that you would not be attending the body image programme?
  - What made you decide to continue with the study, despite not being allocated to the body image programme?
- What did you think of the Macmillan booklet that you were given?
  - Were you aware of this booklet?
  - Did you read the whole Macmillan booklet?
  - Did you find it helpful/unhelpful? Why?
- Did you access any other form of support in relation to your body image?
  - What was this?
  - Was it helpful/unhelpful? Why?
- How did you find completing the questionnaires throughout the study? *(share the questionnaire to remind them of the questions)*
  - What did you think of the format by which you completed these (either paper or online). How could this be improved?
  - How did you find the time it took to complete each questionnaire?
  - How did you find the instructions for completing the different questions in the questionnaire?
  - Did you feel that the questions that you were asked were relevant?
  - Were there other impacts of your body image concerns that were not explored or asked about in the questionnaires?
- What else could we do to help encourage women to take part in a future study and to stay until the end?
- Do you have anything else to add, or any questions?

**12.16 Appendix 16: Interview Schedule: Intervention Participants Finished Study**

For participants who were randomised to the intervention group and completed the whole study (i.e., completed the last questionnaire).

- First of all, how have you found taking part in this study?
- Did you feel that the information provided about the study was clear?
  - Were you able to understand what taking part would involve?
- How did you find the contact and communication from the research team throughout the study?
  - Was each stage of the research (e.g., each questionnaire) clear?
  - Did you feel comfortable corresponding with them?
- How did you feel about the 50% chance that you would be able to attend the body image programme (i.e., being randomised)?
  - How did you feel when you were told that you would be attending the body image programme?
  - Had you not been allocated to the programme, and instead, to the other group, do you think you would have continued with the study? Why/why not?
- How long did you have to wait until you were offered a place on the programme?
  - How did you find the period waiting to attend the programme?
  - How did you cope in the interim?
- What did you think of the ABC programme?
  - Did you find the programme helpful/unhelpful? How? Why?
  - How did you find the actual sessions, including the slides and content covered? Were they clear?
  - What did you think of the activities you were asked to complete during the sessions? Individual v group?
  - What did you think of the reading and activities you were asked to complete in-between the weekly sessions? Too much?
  - What did you think of the cognitive behaviour therapy approach that was used?
  - Were there any topics you particularly enjoyed or found helpful?
  - Were there any topics you found irrelevant or unhelpful?
  - Were there any topics that you felt we missed in the programme?
  - What did you think of the length of each session, and of the overall programme?
  - What did you think of the group size? How did you find the group dynamics?
  - How did you find the psychologist and cancer support specialist delivering the sessions?
  - How did you find the online format? Did you have any difficulty joining the sessions? Did you have any difficulty communicating with others?
  - How could we improve the programme?
  - Do you think the programme should be made available to other women who have gone through breast cancer?
- What did you think of the Macmillan booklet you were also given?
  - Were you aware of this booklet?
  - Did you read the whole Macmillan booklet?
  - Did you find it helpful/unhelpful? Why?
- Did you access any other form of support in relation to your body image?
  - What was this?
  - Was it helpful/unhelpful? Why?
- How did you find completing the questionnaires throughout the study? *(share the questionnaire to remind them of the questions)*
  - What did you think of the format by which you completed these (either paper or online). How could this be improved?
  - How did you find the time it took to complete each questionnaire?
  - How did you find the instructions for completing the different questions in the questionnaire?
  - Did you feel that the questions that you were asked were relevant?
  - Were there other impacts of your body image concerns that were not explored or asked about in the questionnaires?
- What else could we do to help encourage women to take part in a future study and to stay until the end?
- Do you have anything else to add, or any questions?

**12.17 Appendix 17: Demographic and Breast Cancer Information**

**Unique Participant Code**

Your Unique Participant Code will allow us to match your questionnaire responses over time. It will also allow us to identify your data, should you wish for it to be deleted upon choosing to stop taking part in the study.

To create your Unique Participant Code, please enter the **date (day of the month) you were born,** followed by the **first three letters of your mother's maiden name**, and finally, the **last three digits of your mobile phone number**.

For example: If you were born on the 25th of January, your mother's maiden name is Jones, and the last three numbers of your mobile phone number are 705, you would enter **25JON705**

Please enter your code in the box below:

**To help us describe the types of people participating in this study, we would like to gather some information about you. Please remember that all of your answers are anonymous.**

**Recruitment Avenue**

To help us understand how people found out about the study, can you please tell us which of the following applies to you (please select one option below)

- I was sent a letter or email from the NHS trust that treated me
  - Which NHS trust was this? ___________________
- I saw a poster in the waiting room of the NHS trust that treated me
  - Which NHS trust was this? ___________________
- I saw the study advertised via a cancer support organisation
  - Which organisation was this? (please select one option below)
    - Maggie’s
    - Flat Friends
    - Keeping Abreast
    - Breast Cancer Now
    - Live Through This
    - Other (please state) ____________________
      - How was the study advertised? (please select one option below)
        - Via a poster on the wall
        - Via an online forum
        - Via their social media
        - Via email
- I saw the study advertised on social media (**not** by the cancer support organisation)
  - Which social media platform was this? (please select one option below)
    - Instagram
    - Twitter
    - Facebook
    - Other (please state) ____________________
  - Who was the study advertised by? (please select one option below)
    - UWE Bristol
    - Centre for Appearance Research
    - Someone who has had breast cancer themselves and has a large following on social media
    - Other (please state) ____________________
- Other (please state) ____________________

**Demographic Questions**

**To help us describe the types of people participating in this study, we would like to gather some information about you. Please remember that your answers are anonymous.**

How old are you?

______ Years

What region do you live in?

- North East
- North West
- Yorkshire & the Humber
- East Midlands
- West Midlands
- East of England
- London
- South East
- South West
- Wales
- Scotland
- Northern Ireland
- Republic of Ireland

What city/town do you live in?

______

How would you describe your gender identity?

- Woman
- Trans Woman
- Man
- Trans man
- Non-binary
- Other (please state) ____________________

How would you describe your ethnic group?

- Asian/Asian British
- Black/African/Caribbean/Black British
- Mixed/multiple ethnic group
- White
- Other (please state) ____________________

How would you describe your relationship status?

- Divorced
- In a relationship
- Married
- Separated
- Single
- Widowed
- Other (please state) ____________________

Do you have any children?

- Yes
  - How old are they? (open text)
- No

What is your employment status? (select more than one if applicable)

- Home maker
- On prolonged sick leave
- Retired
- Student
- Working full time
- Working part-time
- Unemployed
- Other (please specify) ____________________

What is the highest level of education you have completed?

- GCSE/O-Level or equivalent
- A Level or equivalent
- Higher education certificate or diploma
- Undergraduate degree
- Master’s degree
- PhD or equivalent
- No qualifications
- Other ____________________

What is your menopausal status?

- Premenopausal (experiencing regular periods)
- Peri-menopausal (experiencing period irregularity)
- Menopausal Bleeding (no periods in the past 12 months
- Postmenopausal (no periods for **over** 12 months)
- Medical menopause (menopause caused by treatment)

**Breast cancer-related questions**

**We are now going to ask you some questions about your breast cancer diagnosis and treatment.**

How long ago did you receive your most recent diagnosis of breast cancer?

______ Years

______ Months

What type of breast cancer were you diagnosed with?

- Ductal Carcinoma In Situ (DCIS; also referred to as non-invasive, intraductal)
- Invasive Ductal Carcinoma (IDC)
- Invasive Lobular Carcinoma (ILC)
- Triple Negative Breast Cancer
- Inflammatory Breast Cancer
- Metastatic Breast Cancer
- Other (please specify)
- Not sure

What stage of breast cancer were you diagnosed with?

- 0 - Early form
- 1 or I - Localised
- 2 or II - Early locally advanced
- 3 or III - Late locally advanced
- 4 or IV - Metastasised
- Not sure

How long ago did you finish your primary treatment (first surgery, chemotherapy, or radiotherapy) for breast cancer?

______ Years

______ Months

Are you awaiting further cosmetic procedures? (e.g., breast reconstruction, corrective breast reconstruction, replacing tissue expander with an implant)

- Yes (please specify procedure)
- No

What type of surgical treatment did you receive? (If you underwent mastectomy and reconstruction in 2 separate procedures, please only select "Mastectomy with delayed breast reconstruction". If you are currently awaiting reconstruction, please select “without reconstruction”, as we want to capture your current stage).

- Lumpectomy or wide local excision
- Unilateral mastectomy without breast reconstruction
- Unilateral mastectomy with immediate breast reconstruction
- Unilateral mastectomy with delayed breast reconstruction
- Bilateral mastectomy without breast reconstruction
- Bilateral mastectomy with immediate breast reconstruction
- Bilateral mastectomy with delayed breast reconstruction
- No surgical treatment

If you selected "breast reconstruction”, what type did you have?

- Reconstruction using breast implants
- Reconstruction using your own tissue (flap reconstruction)
- Reconstruction using both breast implants and your own tissue

If you selected “without breast reconstruction”, are you considering having breast reconstruction in the future?

- Yes – I’m currently on the waiting list to have breast reconstruction
- Yes – I’m still thinking and considering whether to have breast reconstruction
- No – I do not wish to have breast reconstruction
- Not sure at this stage

If you selected “without breast reconstruction”, do you wear a breast prosthesis?

- Yes, always
- Yes, sometimes
- No, never

Did you receive chemotherapy?

- Yes
- No

Did you receive radiotherapy?

- Yes
- No

What type of hormonal therapy did you receive?

- Tamoxifen
- Aromatase Inhibitor (e.g. Anastrozole, Exemestane, Letrozole)
- Other (please specify) ____________________
- Not sure
- None

Have you received any other form of treatment that has not already been stated?

- Yes (please state)
- No

Have you received, or are receiving, any form of support (e.g. therapy) to address your body image concerns?

- Yes
- No

If YES, please specify what kind of support you have received or are receiving.

If YES, how helpful was that support?

|  | Not at all helpful  1 | 2 | 3 | 4 | Very helpful  5 |
| --- | --- | --- | --- | --- | --- |
| Please select one option: |  |  |  |  |  |

**12.18 Appendix 18: Kessler Psychological Distress Scale**

| **Distress**  **Kessler Psychological Distress Scale (K10; Kessler et al., 2002)** |
| --- |
| **These questions concern how you have been feeling over the past 30 days. Tick a box below each question that best represents how you have been.**  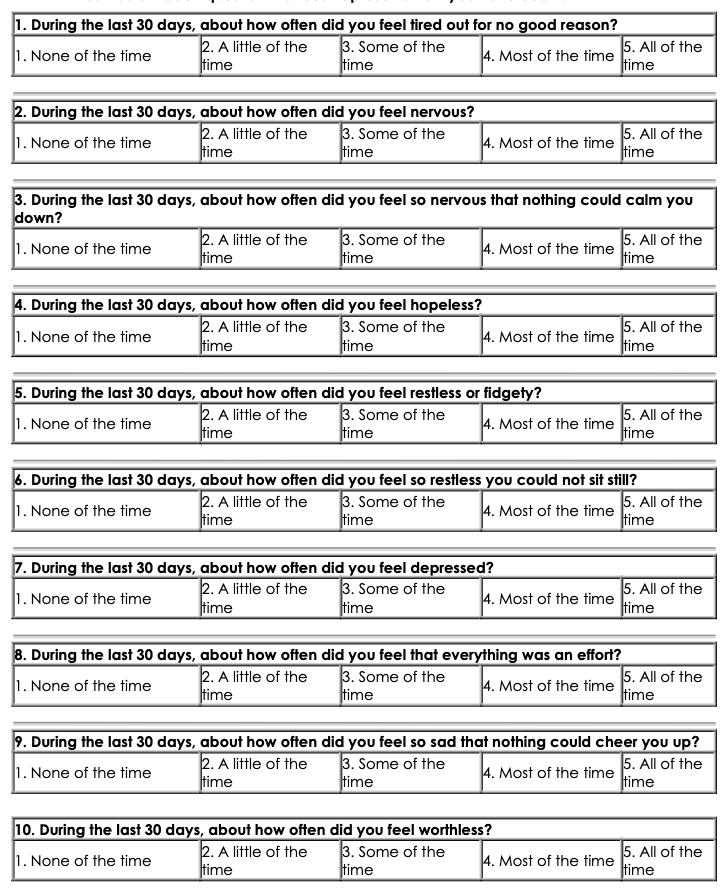 |

**12.19 Appendix 19: Body Appreciation Scale-2**

| **Body Appreciation**  **Body Appreciation Scale-2 (BAS-2; Tylka & Wood-Barcalow, 2015)** |
| --- |
| **Please indicate whether the question is true about you never, seldom, sometimes, often, or always.**  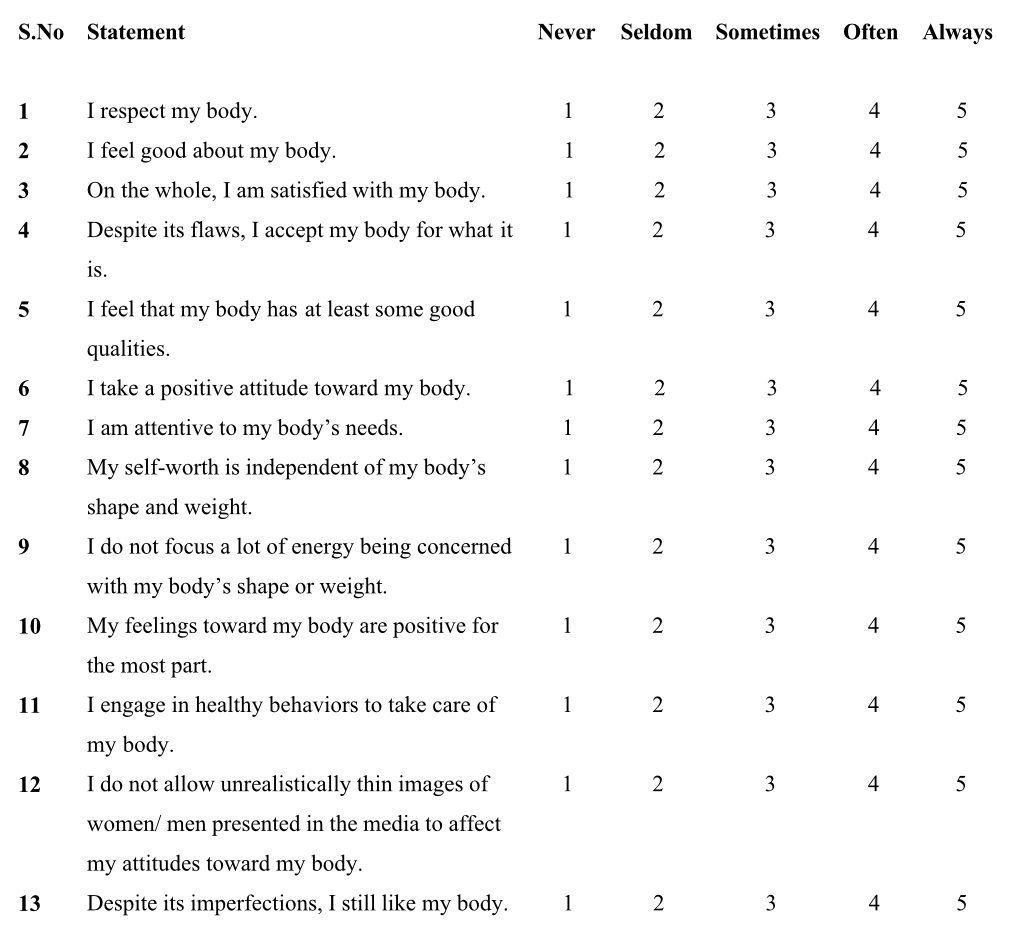 |

| **Health-related Quality of Life**  **Functional Assessment of Cancer Therapy – Breast (FACT-B Version 4): Breast Cancer Subscale (Brady et al., 1997)** |
| --- |
| 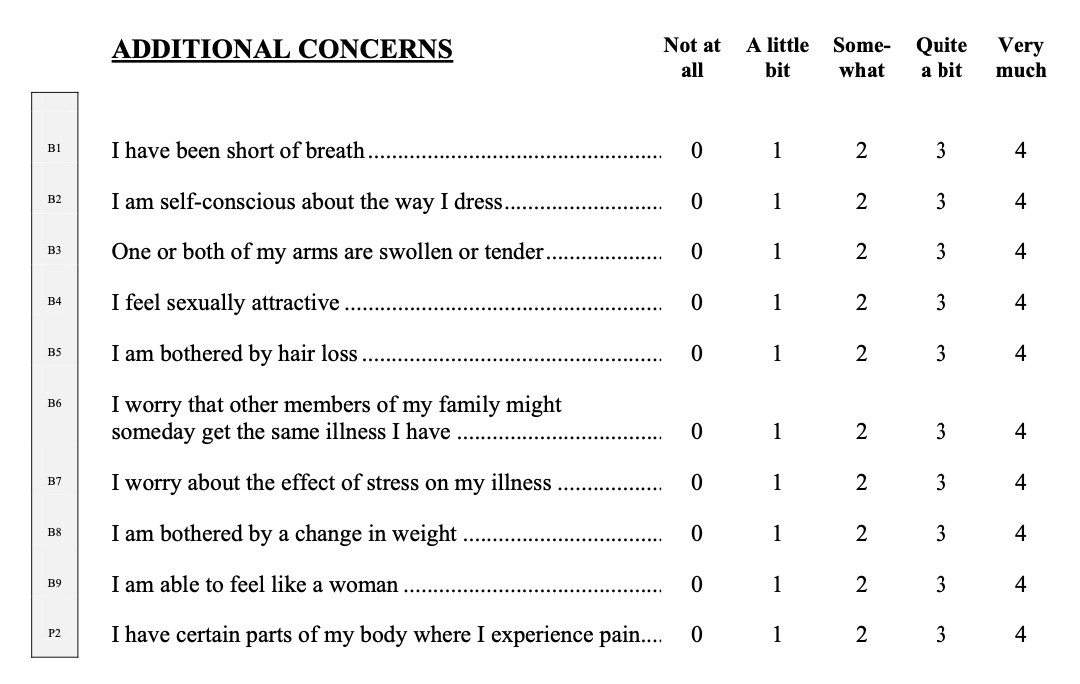**Please circle or mark one number per line to indicate your response as it applied to the past 7 days.**  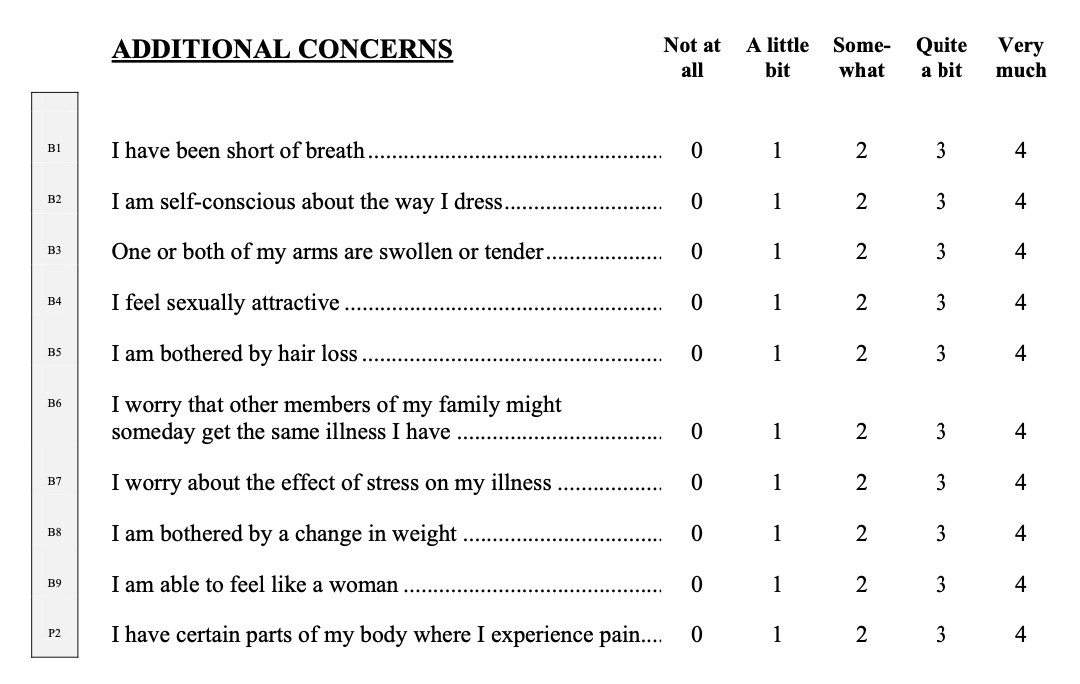 |

**12.20 Appendix 20: Functional Assessment of Cancer Therapy – Breast: Breast Cancer Subscale**

**12.21** **Appendix 21: Hopwood Body Image Scale**

| **Body Image**  **Hopwood Body Image Scale (Hopwood et al., 2001)** |
| --- |
| **Please answer the following questions about how you feel about your body.**   \| **Not at all** \| **A little** \| **Quite a bit** \| **Very much** \| \| --- \| --- \| --- \| --- \| \| 0 \| 1 \| 2 \| 3 \| \| 0 \| 1 \| 2 \| 3 \| \| 0 \| 1 \| 2 \| 3 \| \| 0 \| 1 \| 2 \| 3 \| \| 0 \| 1 \| 2 \| 3 \| \| 0 \| 1 \| 2 \| 3 \| \| 0 \| 1 \| 2 \| 3 \| \| 0 \| 1 \| 2 \| 3 \| \| 0 \| 1 \| 2 \| 3 \| \| 0 \| 1 \| 2 \| 3 \|  1. Have you been feeling self-conscious about your appearance? 2. Have you felt less physically attractive as a result of your disease or treatment? 3. Have you been dissatisfied with your appearance when dressed? 4. Have you been feeling less feminine as a result of your disease or treatment? 5. Did you find it difficult to look at yourself naked? 6. Have you been feeling less sexually attractive as a result of your disease or treatment? 7. Did you avoid people because of the way you felt about your appearance? 8. Have you been feeling the treatment has left your body less whole? 9. Have you felt dissatisfied with your body? 10. Have you been dissatisfied with the appearance of your scar? |

**12.22 Appendix 22: BREAST-Q: Sexual Well-Being Scale**

| **Sexual Wellbeing**  **BREAST-Q: Sexual Well-Being Scale (Pusic et al., 2017)** |
| --- |
| **Thinking of your sexuality, how often do you generally feel:**  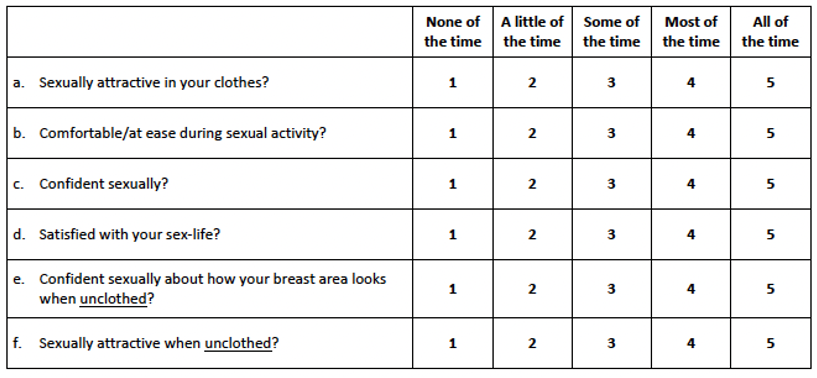 |

**12.23 Appendix 23: Modified version of the Adult Service Use Schedule: T1**

**Accepting your Body after Cancer (ABC)**

**USE OF HEALTH AND SOCIAL CARE SERVICES**

**INSTRUCTIONS**

It would be helpful for us to know what types of health and social care services you have used in the last 3 months. These can be services that you received in a hospital or in the community (for example, in a GP surgery, a community health centre or in your home).

Some of the services listed in this questionnaire may not be relevant to you, but it would still be helpful for us to know this, so **please select ‘No’ if you have not used any of the services listed**.

We understand that you may not remember the exact number of appointments you have had for the services you have used, but **please give us your best guess**.

1. **HOSPITAL SERVICES INVOLVING AN OVERNIGHT STAY**

We would like to know if you have stayed overnight in hospital in the last 3 months.

For each reason listed, please tell us if you have stayed overnight in hospital for this reason by selecting ‘Yes’ or ‘No’.

If the answer is ‘Yes’, please tell us how many nights in total you stayed overnight in hospital for this reason in the last 3 months.

If you had overnight stays in a hospital on more than one occasion for the same reason, please report the total number of nights for all occasions.

| **Reason** | **Yes** | **No** | **If yes, please enter the total number of nights you stayed in hospital for this reason in the last 3 months** |
| --- | --- | --- | --- |
| **Overnight stay in hospital for reasons related to cancer** |  |  |  |
| **Overnight stay in hospital for reasons related to your mental health** |  |  |  |
| **Overnight stay in hospital for any other reason** |  |  |  |
|  |  |  |  |

1. **HOSPITAL SERVICES THAT DID NOT INVOLVE AN OVERNIGHT STAY**

We would like to know if you have had any appointments in hospital in the last 3 months. These are hospital appointments where you DID NOT stay in hospital overnight and are commonly called outpatient or day-patient appointments.

For each reason listed, please tell us if you had any appointments in a hospital for this reason by selecting ‘Yes’ or ‘No’.

If the answer is ‘Yes’, please tell us how many appointments in total you had for each reason in the last 3 months.

| **Reason** | **Yes** | **No** | **If yes, please enter the total number of hospital appointments you have had for this reason in the last 3 months** |
| --- | --- | --- | --- |
| **Hospital appointment for reasons related to cancer** |  |  |  |
| **Hospital appointment for reasons related to your mental health** |  |  |  |
| **Hospital appointment for any other reason** |  |  |  |

1. **ACCIDENT AND EMERGENCY SERVICES**

We would like to know if you have been to an Accident and Emergency Department (A&E) in the last 3 months.

Please tell us if you have been to an A&E by selecting ‘Yes’ or ‘No’.

If the answer is ‘Yes’, please tell us the total number of times you have visited an A&E in the last 3 months.

|  | **Yes** | **No** | **If yes, please enter the total number of times you visited an A&E in the last 3 months** |
| --- | --- | --- | --- |
| **Accident and Emergency (A&E) contacts** |  |  |  |

1. **AMBULANCE**

We would like to know if you have received any treatment by an ambulance crew in the last 3 months. This may include treatment by an ambulance crew in the community and/or travel to hospital in an ambulance.

Please tell us if you have been treated by someone in an ambulance by selecting ‘Yes’ or ‘No’.

If the answer is ‘Yes’, please tell us the total number of times you have been treated by someone in an ambulance in the last 3 months.

|  | **Yes** | **No** | **If yes, please enter the total number of times you were treated by an ambulance crew in the last 3 months** |
| --- | --- | --- | --- |
| **Treatment by an ambulance crew (whether or not you were taken to hospital by the ambulance)** |  |  |  |

1. **COMMUNITY HEALTH AND SOCIAL CARE SERVICES**

We would like to know if you have had any contact with any health or social care professionals or services that took place in the community in the last 3 months.

For each professional or service listed, please tell us if you have had any contact by selecting ‘No’ or ‘Yes’.

If the answer is ‘Yes’, please tell us how many contacts in total you have had with each professional or service in the last 3 months.

**Please include all contacts, whether they took place in person, by telephone or online by video conference.**

|  | **Yes** | **No** | **If yes, please enter the total number of contacts you had in the last 3 months** |
| --- | --- | --- | --- |
| **General practitioner (GP)** |  |  |  |
| **Practice nurse (a nurse based in a GP surgery)** |  |  |  |
| **Cancer nurse/breast cancer nurse in the community (please exclude any contacts with a breast cancer nurse in hospital)** |  |  |  |
| **Any other nurse in the community, for example a district nurse, health visitor or midwife** |  |  |  |
| **Cancer support specialist/cancer support worker** |  |  |  |
| **Social worker, family support worker, or any other social services worker** |  |  |  |
| **Any therapist providing ‘talking therapy’, for example, cognitive behaviour therapy, counselling, mindfulness-based therapy, dialectical behaviour therapy, etc.** |  |  |  |
| **Excluding any contacts with therapists already reported in the previous question, any other mental health worker in the community, for example a psychiatrist, a Community Psychiatric Nurse (CPN), a Community Mental Health Team (CMHT) worker, etc.** |  |  |  |
| **Excluding any contacts already reported in previous questions, any other advice or support relating to cancer (either online, over the phone or in person) from a charitable organisation such as Macmillan Cancer Support, Maggie’s Centres, Penny Brohn, Breast Cancer Now, Keeping Abreast, Flat Friends, Look Good Feel Better, OUTpatients (formerly known as Live Through This), Switchboard Black Women Rising, etc.** |  |  |  |
| **Excluding any contacts already reported in previous questions, any other advice or support relating to mental health (either online, over the phone or in person) from a charitable organisation such as the Samaritans, MIND, Macmillan, etc.** |  |  |  |

| **Any other health or social care services in the community** | **Yes** | **No** | **If yes, please tell us what this service was** |
| --- | --- | --- | --- |
| **Have you used any other health or social care service in the community in the last 3 months?** |  |  |  |
| **Have you used any other health or social care service in the community in the last 3 months?** |  |  |  |
| **Have you used any other health or social care service in the community in the last 3 months?** |  |  |  |

1. **MEDICATION**

We would like to know if you have been prescribed any medication by a doctor or other health professional for mental health or cancer-related reasons in the last 3 months. This might include, for example, medication for depression, anxiety, sleep disorders, etc, or hormone blocking medications.

For each medication category listed below, please tell us if you have been prescribed medication for this reason in the last 3 months by selecting ‘Yes’ or ‘No’.

Please ignore any over-the-counter medications (medications you can buy directly from a chemist/pharmacy that do not require a prescription).

| **Medication for:** | **For example:** | **Yes** | **No** |
| --- | --- | --- | --- |
| **Depression or**  **Anxiety** | Fluoxetine, Prozac, Sertraline, Lustral, Escitalopram, Cipralex, Citalopram, Cimpramil, Fluvoxamine, Faverin, Mirtazapine, Zispin, Venlafaxine, Efexor, Atomoxetine |  |  |
| **Sleep disorders** | Dexamphetamine, Dexedrine, Melatonin, Diazepine, Valium |  |  |
| **Attention Deficit Hyperactivity Disorder (ADHD)** | Methylphenidate, Ritalin, Equasym XL, Concerta XL, Medikinet XL, Dexamphetamine, Dexedrine, Intuniv, Lisdexamfetamine, Elvanse, Atomoxetine, Strattera, Guanfacine XR |  |  |
| **Hormone blocking therapy** | Tamoxifen, letrozole, exemestane, anastrozole, goserelin, zoladex, leuproreline, lucrin |  |  |

| **Any other prescribed medications** | **Yes** | **No** | **If yes, please tell us the name of the medication. If you are not sure of the name, please describe what the medication was for.** |
| --- | --- | --- | --- |
| **Have you been prescribed any other medication in the last 3 months?** |  |  |  |
| **Have you been prescribed any other medication in the last 3 months?** |  |  |  |
| **Have you been prescribed any other medication in the last 3 months?** |  |  |  |

AD-SUS designed by Sarah Byford at King’s College London. For further information please contact: King’s Health Economics, Institute of Psychiatry, Psychology & Neuroscience, King’s College London, De Crespigny Park, London SE5 8AF. Email: [sarah.byford@kcl.ac.uk](mailto:sarah.byford@kcl.ac.uk)

**12.24 Appendix 24: Modified version of the Adult Service Use Schedule: T3**

**Accepting your Body after Cancer (ABC)**

**USE OF HEALTH AND SOCIAL CARE SERVICES**

**INSTRUCTIONS**

It would be helpful for us to know what types of health and social care services you have used since you entered the ABC study approximately 5 months ago. These can be services that you received in a hospital or in the community (for example, in a GP surgery, a community health centre or in your home).

If you are in the ABC therapy group, please **EXCLUDE YOUR USE OF THE ABC THERAPY**, as it is important that ABC research staff are unaware of who is receiving the ABC therapy.

Some of the services listed in this questionnaire may not be relevant to you, but it would still be helpful for us to know this, so **please select ‘No’ if you have not used any of the services listed**.

We understand that you may not remember the exact number of appointments you have had for the services you have used, but **please give us your best guess**.

1. **HOSPITAL SERVICES INVOLVING AN OVERNIGHT STAY**

We would like to know if you have stayed overnight in hospital since you entered the ABC study approximately 5 months ago.

For each reason listed, please tell us if you have stayed overnight in hospital for this reason by selecting ‘Yes’ or ‘No’.

If the answer is ‘Yes’, please tell us how many nights in total you stayed overnight in hospital for this reason since you entered the ABC study approximately 5 months ago.

If you had overnight stays in a hospital on more than one occasion for the same reason, please report the total number of nights for all occasions.

| **Reason** | **Yes** | **No** | **If yes, please enter the total number of nights you stayed in hospital for this reason since you entered the ABC study approximately 5 months ago** |
| --- | --- | --- | --- |
| **Overnight stay in hospital for reasons related to cancer** |  |  |  |
| **Overnight stay in hospital for reasons related to your mental health** |  |  |  |
| **Overnight stay in hospital for any other reason** |  |  |  |

1. **HOSPITAL SERVICES THAT DID NOT INVOLVE AN OVERNIGHT STAY**

We would like to know if you have had any appointments in hospital since you entered the ABC study approximately 5 months ago. These are hospital appointments where you DID NOT stay in hospital overnight and are commonly called outpatient or day-patient appointments.

For each reason listed, please tell us if you had any appointments in a hospital for this reason by selecting ‘Yes’ or ‘No’.

If the answer is ‘Yes’, please tell us how many appointments in total you had for each reason since you entered the ABC study approximately 5 months ago.

| **Reason** | **Yes** | **No** | **If yes, please enter the total number of hospital appointments you have had for this reason since you entered the ABC study approximately 5 months ago** |
| --- | --- | --- | --- |
| **Hospital appointment for reasons related to cancer** |  |  |  |
| **Hospital appointment for reasons related to your mental health** |  |  |  |
| **Hospital appointment for any other reason** |  |  |  |

1. **ACCIDENT AND EMERGENCY SERVICES**

We would like to know if you have been to an Accident and Emergency Department (A&E) since you entered the ABC study approximately 5 months ago.

Please tell us if you have been to an A&E by selecting ‘Yes’ or ‘No’.

If the answer is ‘Yes’, please tell us the total number of times you have visited A&E since you entered the ABC study approximately 5 months ago.

|  | **Yes** | **No** | **If yes, please enter the total number of times you visited an A&E department since you entered the ABC study approximately 5 months ago** |
| --- | --- | --- | --- |
| **Accident and Emergency (A&E) contacts** |  |  |  |

1. **AMBULANCE**

We would like to know if you have received any treatment by an ambulance crew since you entered the ABC study approximately 5 months ago. This may include treatment by an ambulance crew in the community and/or travel to hospital in an ambulance.

Please tell us if you have been treated by someone in an ambulance by selecting ‘Yes’ or ‘No’.

If the answer is ‘Yes’, please tell us the total number of times you have been treated by someone in an ambulance since you entered the ABC study approximately 5 months ago.

|  | **Yes** | **No** | **If yes, please enter the total number of times you were treated by an ambulance crew since you entered the ABC study approximately 5 months ago** |
| --- | --- | --- | --- |
| **Treatment by an ambulance crew (whether or not you were taken to hospital by the ambulance)** |  |  |  |

1. **COMMUNITY HEALTH AND SOCIAL CARE SERVICES**

We would like to know if you have had any contacts with any health or social care professionals or services that took place in the community since you entered the ABC study approximately 5 months ago.

For each professional or service listed, please tell us if you have had any contacts by selecting ‘No’ or ‘Yes’.

If the answer is ‘Yes’, please tell us how many contacts in total you have had with each professional or service since you entered the ABC study approximately 5 months ago.

**Please include all contacts, whether they took place in person, by telephone or online by video conference.**

|  | **Yes** | **No** | **If yes, please enter the total number of contacts you had since you entered the ABC study approximately 5 months ago** |
| --- | --- | --- | --- |
| **General practitioner (GP)** |  |  |  |
| **Practice nurse (a nurse based in a GP surgery)** |  |  |  |
| **Cancer nurse/breast cancer nurse in the community (please exclude any contacts with a breast cancer nurse in hospital)** |  |  |  |
| **Any other nurse in the community, for example a district nurse, health visitor or midwife** |  |  |  |
| **Cancer support specialist/cancer support worker** |  |  |  |
| **Social worker, family support worker or any other social services worker** |  |  |  |
| **Any therapist providing ‘talking therapy’, for example cognitive behaviour therapy, counselling, mindfulness-based therapy, dialectical behaviour therapy, etc.**  ****If you are in the ABC therapy group, please EXCLUDE your use of the ABC therapy**** |  |  |  |
| **Excluding any contacts with therapists already reported in the previous question, any other mental health worker in the community, for example a psychiatrist, a Community Psychiatric Nurse (CPN), a Community Mental Health Team (CMHT) worker, etc.** |  |  |  |
| **Excluding any contacts already reported in previous questions, any other advice or support relating to cancer (either online, over the phone or in person) from a charitable organisation such as Macmillan Cancer Support, Maggie’s Centres, Penny Brohn, Breast Cancer Now, Keeping Abreast, Flat Friends, Look Good Feel Better, OUTpatients (formerly known as Live Through This), Switchboard Black Women Rising, etc.** |  |  |  |
| **Excluding any contacts already reported in previous questions, any other advice or support relating to mental health (either online, over the phone or in person) from a charitable organisation such as the Samaritans, MIND, Macmillan, etc.** |  |  |  |

1. **MEDICATION**

We would like to know if you have been prescribed any medication by a doctor or other health professional for mental health or cancer-related reasons in the last 5 months. This might include, for example, medication for depression, anxiety, sleep disorders, etc, or hormone blocking medications.

For each medication category listed below, please tell us if you have been prescribed medication for this reason since you entered the ABC study approximately 5 months ago by selecting ‘Yes’ or ‘No’.

Please ignore any over-the-counter medications (medications you can buy directly from a chemist/pharmacy that do not require a prescription).

| **Medication for:** | **For example:** | **Yes** | **No** |
| --- | --- | --- | --- |
| **Depression or**  **Anxiety** | Fluoxetine, Prozac, Sertraline, Lustral, Escitalopram, Cipralex, Citalopram, Cimpramil, Fluvoxamine, Faverin, Mirtazapine, Zispin, Venlafaxine, Efexor, Atomoxetine |  |  |
| **Sleep disorders** | Dexamphetamine, Dexedrine, Melatonin, Diazepine, Valium |  |  |
| **Attention Deficit Hyperactivity Disorder (ADHD)** | Methylphenidate, Ritalin, Equasym XL, Concerta XL, Medikinet XL, Dexamphetamine, Dexedrine, Intuniv, Lisdexamfetamine, Elvanse, Atomoxetine, Strattera, Guanfacine XR, |  |  |
| **Hormone blocking therapy** | Tamoxifen, letrozole, exemestane, anastrozole, goserelin, zoladex, leuproreline, lucrin |  |  |

AD-SUS designed by Sarah Byford at King’s College London. For further information please contact: King’s Health Economics, Institute of Psychiatry, Psychology & Neuroscience, King’s College London, De Crespigny Park, London SE5 8AF. Email: [sarah.byford@kcl.ac.uk](mailto:sarah.byford@kcl.ac.uk)

**12.25 Appendix 25: Modified version of the Adult Service Use Schedule: T4**

**Accepting your Body after Cancer (ABC)**

**USE OF HEALTH AND SOCIAL CARE SERVICES**

**INSTRUCTIONS**

It would be helpful for us to know what types of health and social care services you have used since you last completed the ABC questionnaires approximately 3 months ago. These can be services that you received in a hospital or in the community (for example, in a GP surgery, a community health centre or in your home).

If you are in the ABC therapy group, please **EXCLUDE YOUR USE OF THE ABC THERAPY**, as it is important that ABC research staff are unaware of who is receiving the ABC therapy.

Some of the services listed in this questionnaire may not be relevant to you, but it would still be helpful for us to know this, so **please select ‘No’ if you have not used any of the services listed**.

We understand that you may not remember the exact number of appointments you have had for the services you have used, but **please give us your best guess**.

1. **HOSPITAL SERVICES INVOLVING AN OVERNIGHT STAY**

We would like to know if you have stayed overnight in hospital since you last completed the ABC questionnaires approximately 3 months ago.

For each reason listed, please tell us if you have stayed overnight in hospital for this reason by selecting ‘Yes’ or ‘No’.

If the answer is ‘Yes’, please tell us how many nights in total you stayed overnight in hospital for this reason since you last completed the ABC questionnaires approximately 3 months ago.

If you had overnight stays in a hospital on more than one occasion for the same reason, please report the total number of nights for all occasions.

| **Reason** | **Yes** | **No** | **If yes, please enter the total number of nights you stayed in hospital for this reason since you last completed the ABC questionnaires approximately 3 months ago** |
| --- | --- | --- | --- |
| **Overnight stay in hospital for reasons related to cancer** |  |  |  |
| **Overnight stay in hospital for reasons related to your mental health** |  |  |  |
| **Overnight stay in hospital for any other reason** |  |  |  |

1. **HOSPITAL SERVICES THAT DID NOT INVOLVE AN OVERNIGHT STAY**

We would like to know if you have had any appointments in hospital since you last completed the ABC questionnaires approximately 3 months ago. These are hospital appointments where you DID NOT stay in hospital overnight and are commonly called outpatient or day-patient appointments.

For each reason listed, please tell us if you had any appointments in a hospital for this reason by selecting ‘Yes’ or ‘No’.

If the answer is ‘Yes’, please tell us how many appointments in total you had for each reason since you last completed the ABC questionnaires approximately 3 months ago.

| **Reason** | **Yes** | **No** | **If yes, please enter the total number of hospital appointments you have had for this reason since you last completed the ABC questionnaires approximately 3 months ago** |
| --- | --- | --- | --- |
| **Hospital appointment for reasons related to cancer** |  |  |  |
| **Hospital appointment for reasons related to your mental health** |  |  |  |
| **Hospital appointment for any other reason** |  |  |  |

1. **ACCIDENT AND EMERGENCY SERVICES**

We would like to know if you have been to an Accident and Emergency Department (A&E) since you last completed the ABC questionnaires approximately 3 months ago.

Please tell us if you have been to an A&E by selecting ‘Yes’ or ‘No’.

If the answer is ‘Yes’, please tell us the total number of times you have visited A&E since you last completed the ABC questionnaires approximately 3 months ago.

|  | **Yes** | **No** | **If yes, please enter the total number of times you visited an A&E department since you last completed the ABC questionnaires approximately 3 months ago** |
| --- | --- | --- | --- |
| **Accident and Emergency (A&E) contacts** |  |  |  |

1. **AMBULANCE**

We would like to know if you have received any treatment by an ambulance crew since you last completed the ABC questionnaires approximately 3 months ago. This may include treatment by an ambulance crew in the community and/or travel to hospital in an ambulance.

Please tell us if you have been treated by someone in an ambulance by selecting ‘Yes’ or ‘No’.

If the answer is ‘Yes’, please tell us the total number of times you have been treated by someone in an ambulance since you last completed the ABC questionnaires approximately 3 months ago.

|  | **Yes** | **No** | **If yes, please enter the total number of times you were treated by an ambulance crew since you last completed the ABC questionnaires approximately 3 months ago** |
| --- | --- | --- | --- |
| **Treatment by an ambulance crew (whether or not you were taken to hospital by the ambulance)** |  |  |  |

1. **COMMUNITY HEALTH AND SOCIAL CARE SERVICES**

We would like to know if you have had any contacts with any health or social care professionals or services that took place in the community since you last completed the ABC questionnaires approximately 3 months ago.

For each professional or service listed, please tell us if you have had any contacts by selecting ‘No’ or ‘Yes’.

If the answer is ‘Yes’, please tell us how many contacts in total you have had with each professional or service since you last completed the ABC questionnaires approximately 3 months ago.

**Please include all contacts, whether they took place in person, by telephone or online by video conference.**

|  | **Yes** | **No** | **If yes, please enter the total number of contacts you had since you last completed the ABC questionnaires approximately 3 months ago** |
| --- | --- | --- | --- |
| **General practitioner (GP)** |  |  |  |
| **Practice nurse (a nurse based in a GP surgery)** |  |  |  |
| **Cancer nurse/breast cancer nurse in the community (please exclude any contacts with a breast cancer nurse in hospital)** |  |  |  |
| **Any other nurse in the community, for example a district nurse, health visitor or midwife** |  |  |  |
| **Cancer support specialist/cancer support worker** |  |  |  |
| **Social worker, family support worker, or any other social services worker** |  |  |  |
| **Any therapist providing ‘talking therapy’, for example cognitive behaviour therapy, counselling, mindfulness-based therapy, dialectical behaviour therapy, etc.**  ****If you are in the ABC therapy group, please EXCLUDE your use of this therapy**** |  |  |  |
| **Excluding any contacts with therapists already reported in the previous question, any other mental health worker in the community, for example a psychiatrist, a Community Psychiatric Nurse (CPN), a Community Mental Health Team (CMHT) worker, etc.** |  |  |  |
| **Excluding any contacts already reported in previous questions, any other advice or support relating to cancer (either online, over the phone or in person) from a charitable organisation such as Macmillan Cancer Support, Maggie’s Centres, Penny Brohn, Breast Cancer Now, Keeping Abreast, Flat Friends, Look Good Feel Better, OUTpatients (formerly known as Live Through This), Switchboard Black Women Rising, etc.** |  |  |  |
| **Excluding any contacts already reported in previous questions, any other advice or support relating to mental health (either online, over the phone or in person) from a charitable organisation such as the Samaritans, MIND, Macmillan, etc.** |  |  |  |

1. **MEDICATION**

We would like to know if you have been prescribed any medication by a doctor or other health professional for mental health reasons since you last completed the ABC questionnaire approximately 3 months ago. This might include, for example, medication for depression, anxiety, sleep disorders, etc.

For each medication category listed below, please tell us if you have been prescribed medication for this reason since you last completed the ABC study approximately 3 months ago by selecting ‘Yes’ or ‘No’.

Please ignore any over-the-counter medications (medications you can buy directly from a chemist/pharmacy that do not require a prescription).

| **Medication for:** | **For example:** | **Yes** | **No** |
| --- | --- | --- | --- |
| **Depression or**  **Anxiety** | Fluoxetine, Prozac, Sertraline, Lustral, Escitalopram, Cipralex, Citalopram, Cimpramil, Fluvoxamine, Faverin, Mirtazapine, Zispin, Venlafaxine, Efexor, Atomoxetine |  |  |
| **Sleep disorders** | Dexamphetamine, Dexedrine, Melatonin, Diazepine, Valium |  |  |
| **Attention Deficit Hyperactivity Disorder (ADHD)** | Methylphenidate, Ritalin, Equasym XL, Concerta XL, Medikinet XL, Dexamphetamine, Dexedrine, Intuniv, Lisdexamfetamine, Elvanse, Atomoxetine, Strattera, Guanfacine XR, |  |  |
| **Hormone blocking therapy** | Tamoxifen, letrozole, exemestane, anastrozole, goserelin, zoladex, leuproreline, lucrin |  |  |

AD-SUS designed by Sarah Byford at King’s College London. For further information please contact: King’s Health Economics, Institute of Psychiatry, Psychology & Neuroscience, King’s College London, De Crespigny Park, London SE5 8AF. Email: [sarah.byford@kcl.ac.uk](mailto:sarah.byford@kcl.ac.uk)

**12.26** **Appendix 26: EQ-5D-5L**

| **Health-related Quality of Life**  **EQ-5D-5L (Herman er al., 2011)** |
| --- |
| **Under each heading, please select the ONE answer that best describes your health TODAY.**  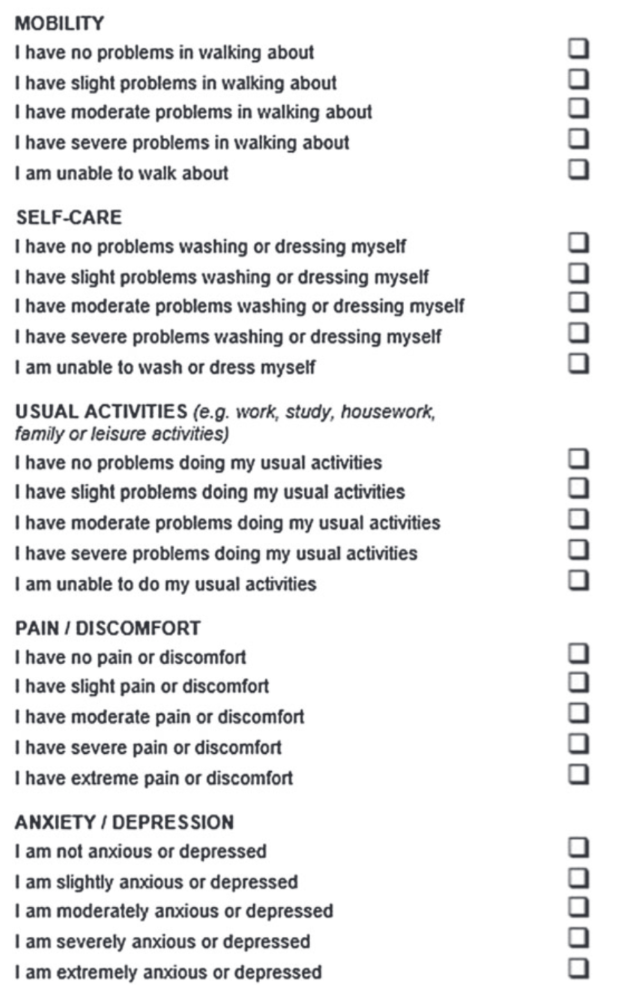  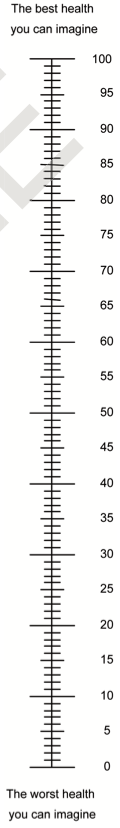We would like to know how good or bad your health is **TODAY**.  This scale is numbered from **0** to **100**.  **100** means the best health you can imagine. **0** means the worst health you can imagine.  Mark an **X** on the scale to indicate how your health is **TODAY**.  Now, please write the number you marked on the scale in the box below.  **YOUR HEALTH TODAY** = |

**12.27 Appendix 27: Recovering Quality of Life-Utility Index**

For each of the following statements, please tick one box that best describes your thoughts, feelings and activities **over the last week**.

| **Over the last week** | None  of the time | Only occasionally | Sometimes | Often | Most or all of the time |
| --- | --- | --- | --- | --- | --- |
| 1. I found it difficult to get started with everyday tasks | □_4_ | □_3_ | □_2_ | □_1_ | □_0_ |
| 2. I felt able to trust others | □_0_ | □_1_ | □_2_ | □_3_ | □_4_ |
| 3. I felt unable to cope | □_4_ | □_3_ | □_2_ | □_1_ | □_0_ |
| 4. I could do the things I wanted to do | □_0_ | □_1_ | □_2_ | □_3_ | □_4_ |
| 5. I felt happy | □_0_ | □_1_ | □_2_ | □_3_ | □_4_ |
| 6. I thought my life was not worth living | □_4_ | □_3_ | □_2_ | □_1_ | □_0_ |
| 7. I enjoyed what I did | □_0_ | □_1_ | □_2_ | □_3_ | □_4_ |
| 8. I felt hopeful about my future | □_0_ | □_1_ | □_2_ | □_3_ | □_4_ |
| 9. I felt lonely | □_4_ | □_3_ | □_2_ | □_1_ | □_0_ |
| 10. I felt confident in myself | □_0_ | □_1_ | □_2_ | □_3_ | □_4_ |

|  | No problems | Slight problems | Moderate problems | Severe problems | Very severe problems |
| --- | --- | --- | --- | --- | --- |
| Please describe your **physical** health (problems with pain, mobility, difficulties caring for yourself or feeling physically unwell) **over the last week** | □_4_ | □_3_ | □_2_ | □_1_ | □_0_ |

For official use

ReQoL-10 Score = ……………

**12.28 Appendix 28: Work and Social Adjustment Scale**

| **Work and Social Functioning**  **Work and Social Adjustment Scale (Mundt et al., 2002)** |
| --- |
| 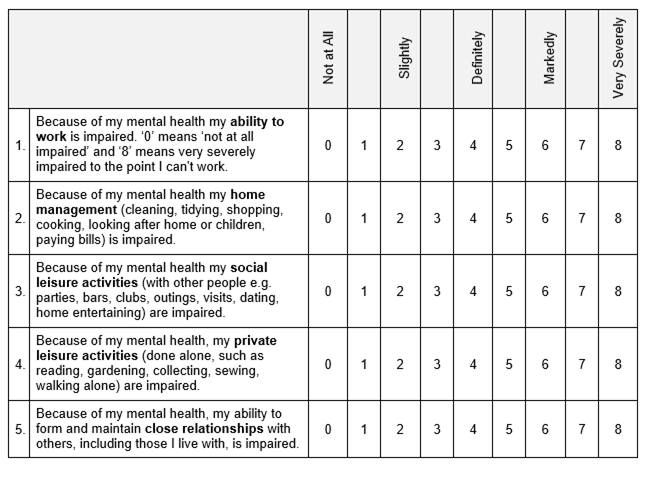**Mental health can affect one’s ability to do certain day-today- tasks in their lives. Please read each item below and respond based on how much your mental health impairs your ability to carry out the activity.** |

**12.29 Appendix 29: Data Management Plan**

| UWE Project manager name: | Dr Helena Lewis-Smith |
| --- | --- |
| Student name, where applicable: | n/a |
| Faculty: | SSS, CHS |
| Project Title: | A feasibility study to inform a Randomised Controlled Trial to evaluate an online-delivered group-based cognitive behavioural therapy (CBT) body image intervention for women who have received treatment for breast cancer |
|  | |

| Research Data Management Plan version number: | 1 |
| --- | --- |
| Date: | 16/04/2024 |
|  | |

| If you have the following reference numbers, please enter them below. | |
| --- | --- |
| PIMS REF number: | Click or tap here to enter text. |
| URESC / FREC / AWESC application numbers: | Click or tap here to enter text. |
| HTSC registration number: | Click or tap here to enter text. |
| GM registration number: | Click or tap here to enter text. |

| Q1. What data will you collect, create or use? Give a brief description. [See Note 1](https://www.uwe.ac.uk/study/library/research-support/manage-your-research-data/planning-your-project/create-a-data-management-plan#note1) |
| --- |
| Informed consent will be obtained from approximately 120 UK-based women (aged 18 years+) who have been treated for breast cancer. Personal information relating to contact details will be collected when participants provide informed consent.  Participants will complete several self-report quantitative and qualitative measures at different assessment time points throughout the study exploring their feedback on the whole research process (i.e., exploring feasibility of the study) and the body image programme being evaluated. They will also be asked to provide their own demographic and cancer related information and completed validated self-report measures relating to body image, intimacy, and quality of life.  Anonymised data related to the programme attendance and engagement, as well as Microsoft Teams recordings of the sessions, will be collected by programme facilitators, and shared with the research team.  Participants taking part in the recorded audio-recorded interviews will be given a pseudonym, and thus will not be identifiable. These interviews will take place via Microsoft Teams.  Other quantitative data collected throughout the trial (e.g., withdrawal rates) will be collected by the research team and noted on SPSS. |
| Q2. How will you collect, create or access the data? [See Note 2](https://www.uwe.ac.uk/study/library/research-support/manage-your-research-data/planning-your-project/create-a-data-management-plan#note2) |
| Data will not be collected until NHS IRAS, HRA, and UWE REC approvals have been received. All data will be handled and stored securely and in compliance with the Data Protection Act and GDPR.  Informed consent will be received from participants prior to any research data being collected. Participants will be fully informed about the study prior to providing consent, through reading of the participant information sheet and discussions with the research team. They will be given the option of completing this online via the survey software Qualtrics or via a paper version of the form. If they prefer the first option, they will be sent a weblink to the online consent form. Once completed, they will be emailed a signed copy for their records. If they prefer the second option, they will be mailed the consent form and a pre-addressed, stamped envelope to return the signed consent form via the post. Some of these participants will be purposefully sampled to take part in an interview to provide feedback about the research process or the body image programme. They will be required to read a separate participant information sheet and complete a separate consent form. The similar process as above will take place. The signed consent form will be held on UWE’s OneDrive for Business and only accessed by the research team at UWE (and paper versions will be scanned into an online file, and subsequently securely destroyed).  Participants will complete several self-report quantitative and qualitative measures at different assessment time points throughout the study exploring their feedback on the whole research process (i.e., exploring feasibility of the study) and the body image programme being evaluated. They will also be asked to provide their own demographic and cancer related information and completed validated self-report measures relating to body image, intimacy, and quality of life.  This data will be collected online via Qualtrics (whereby participants are sent the appropriate weblink), an online survey platform. Qualtrics uses Transport Layer Security (TLS) encryption (also known as HTTPS) for all transmitted data. This data will be downloaded into SPSS (a statistical software package) and NVivo (a qualitative data analysis software package) with files held on OneDrive for Business.  If participants to complete these measures by paper, they will be sent these in the post with a stamped pre-addressed envelope to return to the research team. Once returned, these will be stored in a locked filing cabinet in a locked office at the UWE Bristol, to which only the research team will have access. Once the data is inputted directly into SPSS and NVivo files on OneDrive for Business, the paper versions will be securely destroyed.  Participants will be identified by a unique participant ID (devised based on a combination of non-identifying but memorable data e.g., the number of siblings they have, the last three digits of their phone number) for the above data collection, and files linking participant ID to their contact details will be held on the UWE Bristol OneDrive for Business, with only the UWE research team (including the Statistician) having access to this. Anonymous raw data relating to the self-report quantitative and qualitative measures (including the validated self-report measures relating to body image, intimacy, and quality of life) will only be shared beyond UWE with Kings College London, so that the Health Economist can conduct their analyses. The data will be stored on their secure University OneDrive for Business. A collaboration contract has been signed between UWE and Kings College London, with includes expectations on how data will be processed and held.  Anonymised data related to the programme attendance and engagement, as well as Microsoft Teams recordings of the sessions, will be collected by programme facilitators, and shared with the research team, which will then be copied over onto UWE Bristol OneDrive for Business. No participants will be identifiable via these recordings, and only the UWE research team will access to these.  Participants taking part in the Microsoft Teams audio-recorded interviews will be given a pseudonym, and thus minimises the chances of being identifiable. The recordings via Teams will be saved into a file on the West of England OneDrive for Business, and once transcribed, they will be deleted. Only the UWE research team will have access to this data.  Other quantitative data collected throughout the trial (e.g., withdrawal rates) will be collected by the research team and saved on a file on the University of the West of England OneDrive for Business. Only the UWE research team will have access to this data. |
| Q3. Please classify your data here as public, restricted or confidential. [See Note 3](https://www.uwe.ac.uk/study/library/research-support/manage-your-research-data/planning-your-project/create-a-data-management-plan#note3) |
| Confidential |
| Q4. How will the data be stored and backed up at all stages during its life course? [See Note 4](https://www.uwe.ac.uk/study/library/research-support/manage-your-research-data/planning-your-project/create-a-data-management-plan#note4) |
| All data will be handled and stored securely and in compliance with the Data Protection Act, GDPR, and UWE’s Information Security Policy.  All electronic data will be stored on UWE Bristol OneDrive and backed up by the CI, Helena Lewis-Smith. Each back up name will include a version number, the date, and the initials of the person conducting the backup. It will be backed up at one-week intervals.  All paper data will be kept in a locked cabinet until it is converted to electronic data (consent forms will be scanned and self-report measure data entered manually). Once the paper data has been converted and securely stored electronically, the paper versions will be securely destroyed. |
| Q5. How will the data be documented, described and maintained? [See Note 5](https://www.uwe.ac.uk/study/library/research-support/manage-your-research-data/planning-your-project/create-a-data-management-plan#note5) |
| Document version management will be achieved with any edits to documents resulting in a new version with a version number and date clearly displayed on each page of the file name, as well as added at the end of the file name. Version control will be recorded in an appendix in the protocol.  All self-report measure data will be in CSV file format as produced by Qualtrics, which will then be transferred in .sav format in SPSS, where quantitative data analysis will take place. All qualitative data in .doc format (in Microsoft word) will be transferred into NVivo format, where qualitative data analysis will take place.  A datalog file providing details of the data (date, name and purpose of the data, the creator, details of how the data was created/analysed, explanations of any codes or abbreviations used) will be maintained. A ‘coding book’ for data files will also be created for the Health Economist and Statistician to ensure that they are aware of what different variables mean. |
| Q6. How will your data be processed? [See Note 6](https://www.uwe.ac.uk/study/library/research-support/manage-your-research-data/planning-your-project/create-a-data-management-plan#note6) |
| All personal data (i.e. names and contact details) will be stored separately from the anonymised research data (i.e. self-report measures). The personal data and participant ID will be linked in the same document, but this will only be accessible by the CI (Helena Lewis-Smith) and Research Fellow/Study Manager on the study. Personal data will only be accessed by these two individuals to enable them to contact participants at each respective assessment timepoint (i.e., to share the weblink or paper-based questionnaire with them) and to share the findings of the study (if participants indicated they would like to receive information about these).  Once this personal data is no longer needed (i.e. at the end of the research, or once the participant has been informed of the results of the study if they have requested this), it will be deleted. All research data will be anonymised (i.e., it will include no personal identifying information) and uploaded (along with the ‘coding book’ and ‘Datafile log’) to UWE Bristol OneDrive for Business. This will be shared with the Statistician based at UWE via UWE Bristol OneDrive for Business) and the Health Economist at Kings College London, who will store these files on their university’s OneDrive for Business. A collaboration contract has been signed between UWE Bristol and collaborating universities, with includes formal agreement how data will be processed and held.  No one else will have access to the quantitative data. |
| Q7. Does the Data Protection Act (2018) apply to your research? [See Note 7](https://www.uwe.ac.uk/study/library/research-support/manage-your-research-data/planning-your-project/create-a-data-management-plan#note7) |
| Yes, as some personal data will be collected. Such data will be collected and processed in line with GDPR. The minimum personal data required to take part in the study will be collected, e.g. name and contact details. This will only be access by the CI and Study Manager. All other data which could be considered personal data (e.g., age, sexuality, breast cancer diagnosis, time since treatment) will be anonymised. Personal data will only be accessed by Helena Lewis-Smith (CI) and the Research Fellow/Study Manager on the study to enable them to contact participants at each respective assessment timepoint (i.e., to share the weblink or paper-based questionnaire with them) and to share the findings of the study (if participants indicated they would like to receive information about these). This personal data will be securely deleted at the end of the study.  A DPIA is not required as the personal data collected will not be high risk. |
| Q8. Export controls and other legislation and regulation. [See Note 8](https://www.uwe.ac.uk/study/library/research-support/manage-your-research-data/planning-your-project/create-a-data-management-plan#note8) |
| None applicable |
| Q9. What Intellectual Property will be created or used in this research? [See Note 9](https://www.uwe.ac.uk/study/library/research-support/manage-your-research-data/planning-your-project/create-a-data-management-plan#note9) |
| A collaboration contract has been signed between UWE Bristol and the other collaborating universities, which sets out formal agreements relating to intellectual property. The IP created will be the research data and subsequent analyses, which will be owned by UWE Bristol. |
| Q10. What are your plans for long-term preservation and data sharing, where appropriate, and data disposal? [See Note 10](https://www.uwe.ac.uk/study/library/research-support/manage-your-research-data/planning-your-project/create-a-data-management-plan#note10) |
| Upon termination of data collection and sharing of findings with participants, all personal data relating to participants (e.g., names, contact details) will be securely deleted.  Once all reports, conference presentations, and publications are published and/or completed, fully anonymised data will be archived via UWE Bristol OneDrive for the purposes of further analysis within the UWE research team (e.g., answering other research questions of importance) and data sharing (as per the NIHR’s guidelines). When publishing the findings of the research, a data sharing statement will be shared, which will state that researchers can contact the UWE research team to enquire about accessing the underpinning research data. Data access requests will be managed following UWE Bristol policies and practices, and release of data will be subject to a data use agreement between UWE Bristol and the third party requesting the data.  Data will be stored at UWE Bristol for as long as it carries research value, up to a maximum of 10 years. |
| Q11. Who is responsible for enacting the different elements of the research data management plan? [See Note 11](https://www.uwe.ac.uk/study/library/research-support/manage-your-research-data/planning-your-project/create-a-data-management-plan#note11) |
| The CI (Helena Lewis-Smith) has overall responsibility for enacting the data management plan. Under her supervision, the Research Fellow/Study Manager on the project (Dr Abigail Jones) will conduct the majority of the day-to-day data management. In addition, other members of the research team may conduct some elements of data management (such as preparing data for analysis or storage). A contract has been signed between UWE Bristol and the funder (NIHR), which sets out formal agreements relating to the research. A contract has also been signed between UWE Bristol and collaborating universities, which sets out formal agreements relating to the research. |
| Q12. What resources are needed to deliver the plan, and are these available? [See Note 12](https://www.uwe.ac.uk/study/library/research-support/manage-your-research-data/planning-your-project/create-a-data-management-plan#note12) |
| No additional resources are needed to deliver the plan. The staff time required is covered as part of the funding for the project, and the IT required (laptops, OneDrive access etc) is available as standard employment equipment. |

**12.30 Appendix 30: Protocol Amendment History**

| **Protocol Version Number** | **Date** | **Summary of Amendments** |
| --- | --- | --- |
| 2 | 23.05.2024 | - Main Study PIS updated: - The ‘Participant Privacy Information’ section was replaced with the HRA's GDPR language. - The following text was added to Section 19: “If you stop being part of the study due to losing your capacity, we will keep the anonymous information but will not keep any identifiable information on you.” - The following text was added to Section 17: “If you experience harm or injury as a result of any study activities, you may be eligible to receive compensation.” - Section 12 has now been edited from “However, your name will not be used in any of these outputs.” to “Whilst anonymised quotes may be used in these outputs, your name will not be used.” - Interview Study PIS updated: - The ‘Participant Privacy Information’ section was replaced with the HRA's GDPR language. - The following text was added to Section 13: “If you experience harm or injury as a result of any study activities, you may be eligible to receive compensation." - Protocol updated: - The following text has been added to Section 9 (page 34): “For safety reporting and regulatory purposes, the end of the study will be when all data collection is complete, and data is finalised (e.g., transcripts complete). The REC will be informed about the end of the study within 90 days of the final follow-up visit taking place.” - Page 24: Corrected description of the BAS-2 from 10 to 13 items. - Page 25: Corrected description of the BREAST-Q from being rated from 0-100, to a 5-point scale. |
| 3 | 24/10/24 | - Follow-up questionnaires updated:   - Inclusion of additional demographics question at three-month follow-up assessment   - Inclusion of ABC acceptability questions at three- and six-month follow-up assessments - Protocol Updated:   - Pages 19 & 20: Added details of follow-up ABC acceptability questions   - Page 22: Updated Table 1 Schedule of Assessments   - Page 24: Added details of additional demographics question   - Page 119: Follow-up ABC acceptability questions added to appendix 31   - Page 120: Additional demographics question added to Appendix 32   - Pages 6 to 8: Contents table updated |

**12.31: Appendix 31: Follow-up ABC Acceptability Questions**

Your thoughts about the ABC programme and its impacts may have changed over the last few months. Therefore, reflecting on how you feel now, how **helpful** did you find the programme overall?

|  | Not at all helpful  1 | 2 | 3 | 4 | Very helpful  5 |
| --- | --- | --- | --- | --- | --- |
| Please select one option: |  |  |  |  |  |

Would you like to tell us more about this?

**12.32: Appendix 32: Additional Demographics Question**

How would you describe your sexual orientation? *For example: straight, lesbian, bisexual etc.*
