# Supplementary figures and images for: Accepting your Body after Cancer (ABC), a group-based online intervention for women treated for breast cancer: study protocol for a feasibility randomised controlled trial
Source: BMJ Open. 2025 Jan 22;15(1):e097817. doi: 10.1136/bmjopen-2024-097817 (PMC11784239; doi:10.1136/bmjopen-2024-097817)

**
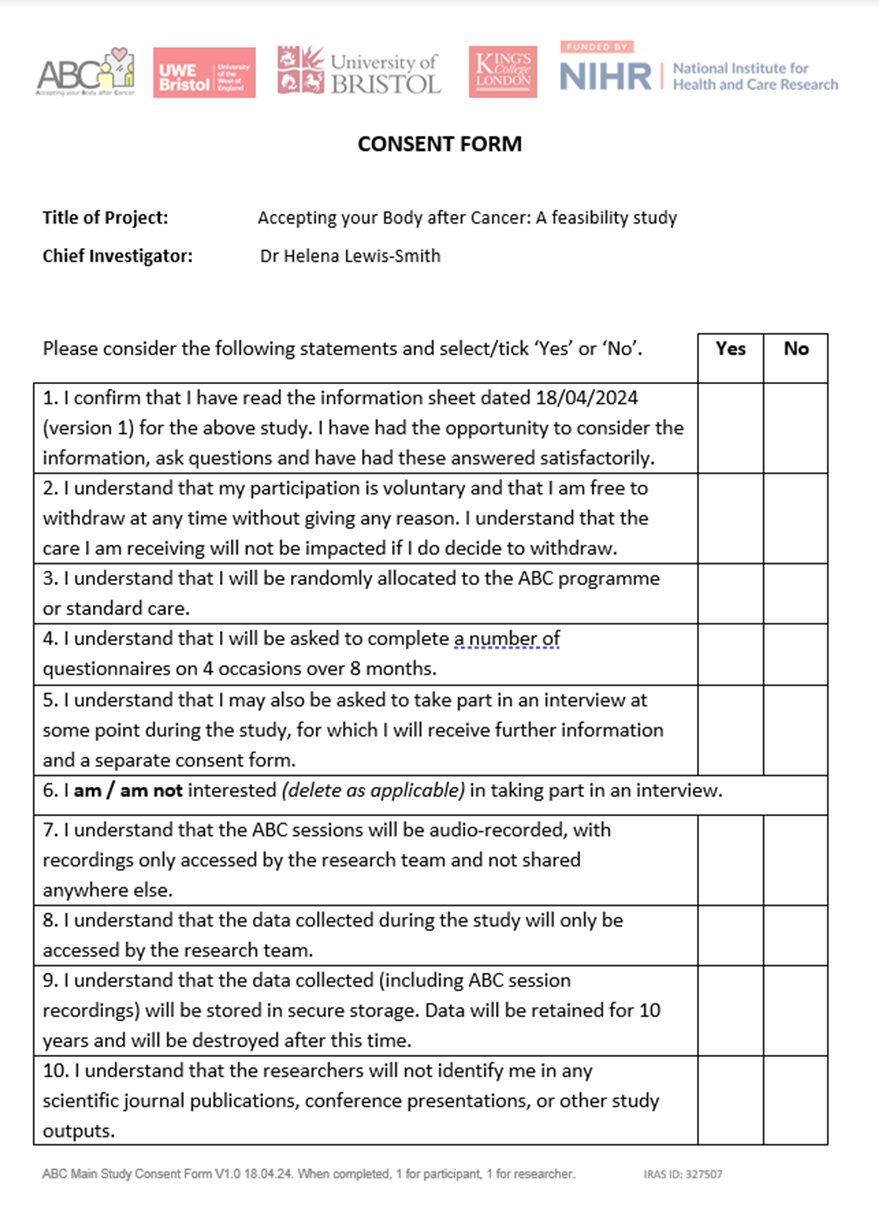
**

**
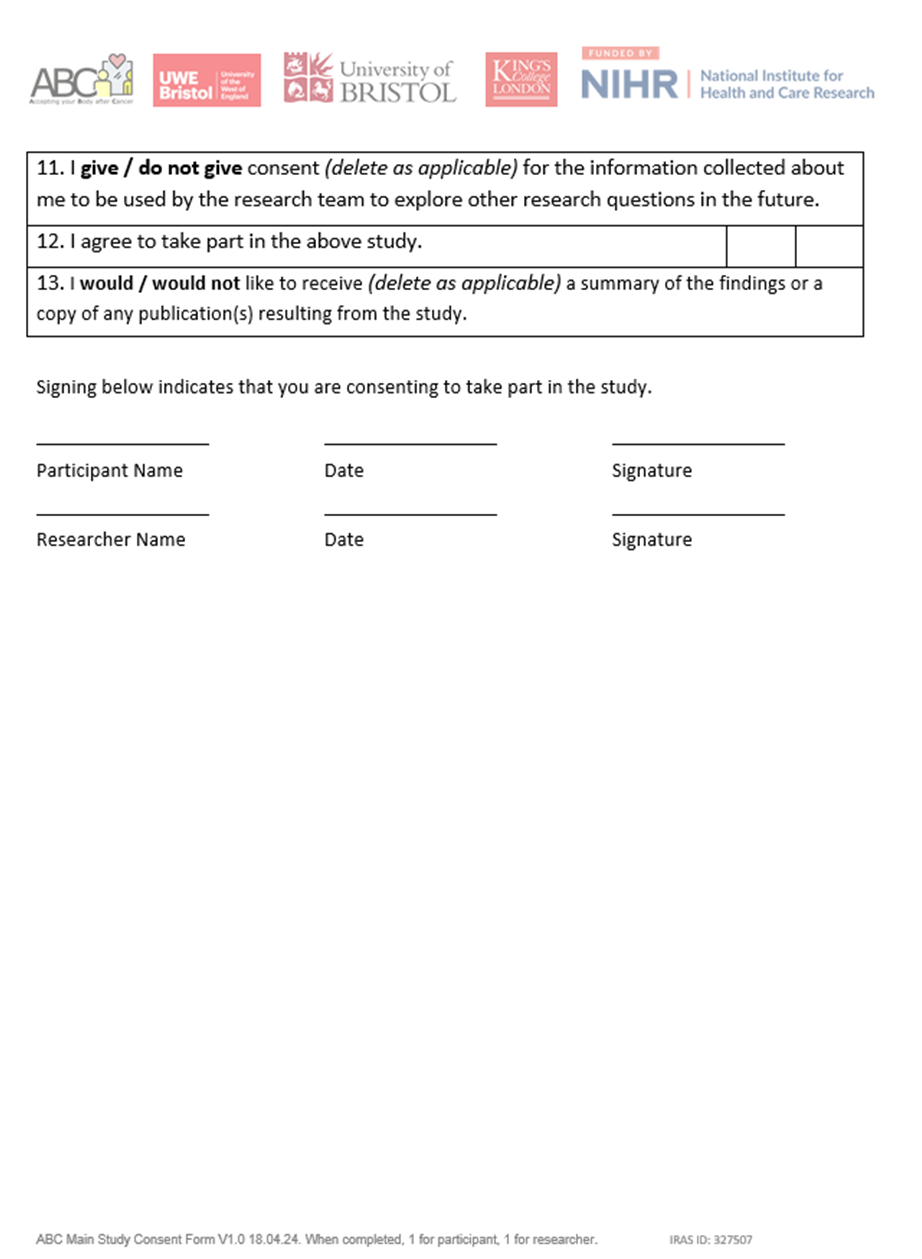
**

Supplement: online supplemental file 2 [file bmjopen-15-1-s002.docx]
